# Tracing the origin and evolution history of methylation-related genes in plants

**DOI:** 10.1186/s12870-019-1923-7

**Published:** 2019-07-12

**Authors:** Liuling Pei, Lin Zhang, Jianying Li, Chao Shen, Ping Qiu, Lili Tu, Xianlong Zhang, Maojun Wang

**Affiliations:** National Key Laboratory of Crop Genetic Improvement, Huazhong Agricultural University, Wuhan, 430070 Hubei China

**Keywords:** DNA methylation, Gene origin, Duplication, Gene structure, Phylogenetic analysis

## Abstract

**Background:**

DNA methylation is a crucial epigenetic modification, which is involved in many biological processes, including gene expression regulation, embryonic development, cell differentiation and genomic imprinting etc. And it also involves many key regulatory genes in eukaryotes. By tracing the evolutionary history of methylation-related genes, we can understand the origin and expansion time of these genes, which helps to understand the evolutionary history of plants, and we can also understand the changes of DNA methylation patterns in different species. However, most studies on the evolution of methylation-related genes failed to be carried out for the whole DNA methylation pathway.

**Results:**

In this study, we conducted a comprehensive identification of 33 methylation-related genes in 77 species, and investigated gene origin and evolution throughout the plant kingdom. We found that the origin of genes responsible for methylation maintenance and demethylation evolved early, while most de novo methylation-related genes appeared late. The methylation-related genes were expanded by whole genome duplication and tandem replication, but were also accompanied by a large number of gene absence events in different species. The gene length and intron length varied a lot in different species, but exon structure and functional domains were relatively conserved. The phylogenetic relationships of methylation-related genes were traced to reveal the evolution history of DNA methylation in different species. The expression patterns of methylation-related genes have changed during the evolution of species, and the expression patterns of these genes in different species can be clustered into four categories.

**Conclusions:**

The study describes a global characterization of DNA methylation-related genes in the plant kingdom. The similarities and differences in origin time, gene structure and phylogenetic relationship of these genes lead us to understand the evolutionary conservation and dynamics of DNA methylation in plants.

**Electronic supplementary material:**

The online version of this article (10.1186/s12870-019-1923-7) contains supplementary material, which is available to authorized users.

## Background

The classical DNA methylation process is that the methyl group of S-adenosylmethionine is transferred to cytosines by different DNA methyltransferases, forming C5-methylcytosine (5-mC) [1]. In plants, DNA methylation is found at cytosines (C) in three sequence contexts, including CG, CHG, and CHH (H = A, T, or C), which represents a difference from the predominant DNA methylation at the CG sequence context in animals [2–4]. DNA methylation is important for plant growth and development, and plays a role in genome imprinting, vernalization, response to biotic and abiotic stresses, and heterosis [1, 5–11].

In *Arabidopsis thaliana*, DNA methylation in all sequence contexts (CG, CHG, and CHH) is established de novo by RNA-directed DNA methylation (RdDM) [5, 12–16]. The maintenance of methylation at CG and CHG sites depends primarily on DNA METHYLTRANSFERASE 1 (MET1) [5] and CHROMOMETHYLASE3 (CMT3) [17]. The methylation at CHH sites can’t be maintained and must be re-established by both CHROMOMETHYLASE2 (CMT2) and RdDM [18]. In heterochromatin, de novo DNA methylation and maintenance require the action of DECREASE IN DNA METHYLATION1 (DDM1) [18]. DDM1 is one kind of nucleosome remodelers and can change the composition and position of the nucleosome, which makes chromatin more accessible to DNA methyltransferases [19]. Some active markers on histones are removed by JUMONJI 14 (JMJ14) [20], HISTONE DEACETYLASE 6 (HDA6) [21], UBIQUITIN-SPECIFIC PROTEASE 26 (UBP26) [22] and the repressed marker H3K9me2 is maintained by SUVH4 [23], which can maintain DNA methylation or the silencing state caused by DNA methylation.

The RdDM pathway relies on a specialized transcription machinery that is centered on two plant-specific homologs of RNA polymerase II (Pol II): Pol IV and Pol V [24–27]. In brief, the canonical RdDM process involves the following steps: Firstly, in the process of Pol IV-dependent small interfering RNAs (siRNAs) biogenesis, the single-stranded RNA is transcribed by Pol IV followed by transforming into double-stranded RNA under the action of RNA-DEPENDENT RNA POLYMERASE (RDR2) [28] with the assistance of chromatin remodeler CLASSY 1 (CLSY1) [29]; Then, double-stranded RNA is cut into 24-nt siRNAs under the action of DICER-LIKE 3 (DCL3) [30]; The 3′ end of the 24 nt siRNA is methylated by HUA ENHANCER 1 (HEN1) [31]; Finally, one strand of methylated 24-nt siRNA is loaded onto ARGONAUTE 4 (AGO4) [32]. Secondly, in the process of Pol V-mediated de novo methylation, the chromatin remodeler DEFECTIVE IN RNA-DIRECTED DNA METHYLATION 1 (DRD1) will open the DNA duplex and form a DDR complex with DEFECTIVE IN MERISTEM SILENCING 3 (DMS3) and RNA-DIRECTED DNA METHYLATION 1 (RDM1) to stabilize a loose chromatin state [33, 34], and MICRORCHIDIA 6 (MORC6) will assist in stabilizing the loose state [35, 36]; Pol V is recruited to the target site with the assistance of SU(VAR)3–9 HOMOLOG 2/9 (SUVH2/9) [37]; The AGO4 protein loaded with siRNAs is also recruited to the target site under the action of CARBOXY-TERMINAL DOMAIN (CTD) of Pol V and KOW DOMAIN-CONTAINING TRANSCRIPTION FACTOR 1 (KTF1), then siRNAs pair with the scaffold RNA transcribed by Pol V [38]; RDM1 can connect AGO4 protein with DOMAINS REARRANGED METHYLTRANSFERASE 1/2 (DRM1/2) to activate de novo establishment of DNA methylation. Thirdly, in the process of chromatin alteration, the INVOLVED IN DE NOVO 2 (IDN2) - IDN2 PARALOGUE (IDP) complex interacts with the SWI/SNF complex to change nucleosome positioning, thus making DNA more easily to be methylated [39].

Although DNA methylation is a relatively stable marker which can be inherited by the daughter cells after cell division, it’s still reversible and dynamically regulated. In eukaryotes, the level of DNA methylation is not only related to the establishment and maintenance processes, but also related to the process of demethylation. The DNA demethylation is divided into passive demethylation and active demethylation. Passive demethylation is that the level of DNA methylation is reduced due to the loss of methylation maintenance in the process of DNA replication. Active demethylation is the process in which the 5-methylcytosine bases are replaced with non-methylated cytosines by DNA glycosylases/lyases and other enzymes [40]. In *Arabidopsis*, the main DNA glycosylases include Repressor of Silencing 1 (ROS1), DEMETER (DME), DME-like 2 (DML2) and DME-like 3 (DML3) [5]. These four demethylases can excise 5-mC from all sequence contexts. Previous studies showed that *ROS1* could conduct active DNA demethylation in all tissues [41], and *DME* was preferentially expressed in central cell and synergid of the female gametophyte [40, 42]. In addition, an EFFECTOR OF TRANSCRIPTION (ET) factor was characterized with a potential role in demethylation [43].

In recent years, many studies explored the evolution of methylation-related genes in plants. In hexaploid wheat, all *MET1* genes were divided into three categories, and these genes were derived from whole genome duplication within the grass family and gene duplication which occurred specifically in the *Triticeae* tribe [44]. The discovery of DNA methyltransferases in different plants and fungi indicated that *MET1* was highly conserved [11]. The evolution analysis of RNA polymerase subunits showed that genes encoding Pol IV and Pol V evolved rapidly relative to Pol II. An analysis of genes in the RdDM pathway in 15 species showed that RdDM appeared in early land plants [45]. Recently, the origin of the CMT family was deduced in terrestrial plants by using data in 443 species [46].

Here, we explored phylogenetic relationships of methylation-related genes on a rather long evolutionary scale from algae to flowering plants. We identified 33 methylation-related genes in 77 species, and explored the origin and evolution of genes in de novo methylation, maintenance methylation and demethylation processes. The gene structural changes were investigated by an exon-intron structure analysis, and the functional changes were studied by a domain analysis. In addition, a phylogenetic analysis showed that these methylation-related genes were divided into three categories, i.e., early plants, monocotyledon plants and dicotyledon plants.

## Results

### The inconsistency in the origin of methylation-related genes

**Fig. 1:**
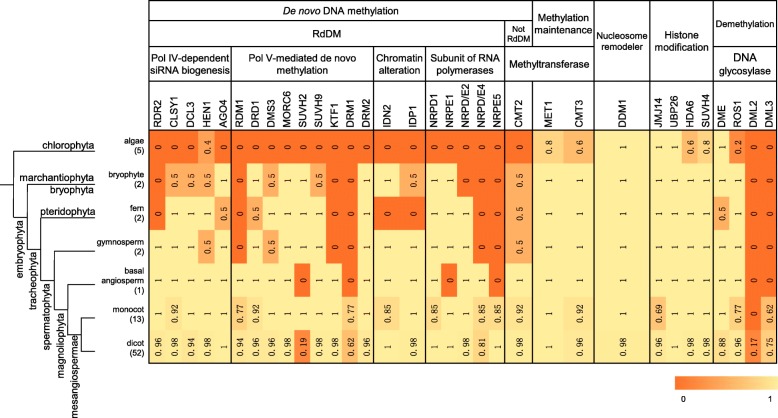
The origin of methylation-related genes. The numbers in brackets represent the species number in each category. The numbers in the table represent the ratio of the number of species containing methylation-related genes to the total number of species

The methylation-related genes have been comprehensively characterized in *A. thaliana*. In this study, all these genes were divided into five categories including eight groups. The five categories included genes primarily functioning in de novo DNA methylation, methylation maintenance, nucleosome remodeling, histone modification and demethylation (Fig. 1). The canonical RdDM process of de novo DNA methylation includes three steps: Pol IV-dependent siRNA biogenesis, Pol V-mediated de novo methylation and chromatin alteration. We identified methylation-related genes for all these categories in 77 species involving of algae and plants (Additional file 4: Table S1). These algae include five species, and plants include species from bryophyte, fern, gymnosperm, basal angiosperm, monocot and dicot. We summarized the content of methylation-related genes in each species (Additional file 5: Table S2).

In each of the five categories, we calculated the ratio of the number of species containing methylation-related genes to the total number of species, and explored the evolution of these genes from algae to higher flowering plants (Fig. 1). In the RdDM process, we investigated 5 genes required for Pol IV-dependent siRNA biogenesis. Of them, only *HEN1* is found in algae, and some genes in the bryophytes, ferns, monocots and dicots are missing. There are 9 genes involved in Pol V-mediated de novo methylation. *SUVH2* is absent in basal angiosperm, and is also absent in 71% of dicots, whereas it is found in all monocots. There are 2 genes involved in the chromatin alteration process, they are absent in the algae. For RNA polymerases, we explored the NUCLEAR RNA POLYMERASE D1 (*NRPD1*), *NRPE1*, *NRPD2/E2*, *NRPD4/E4*, and *NRPE5*. Interestingly, all the five subunits are not found in algae. The methylation of the CHH site is found to be re-established by the process of de novo DNA methylation during each cell cycle, while de novo DNA methylation of the CHH site includes RdDM and another RdDM-independent way [12]. The latter requires the participation of *CMT2* which is absent in algae. Collectively, we conclude that only a few genes involved in de novo DNA methylation appeared in algae, however, these genes became widespread from bryophytes to higher plants. Therefore, the de novo DNA methylation process may appear after divergence of chlorophyta and embryophyta.

In the process of maintenance of DNA methylation, different enzymes work in different ways. The methylation of CG requires the participation of *MET1* which is basically present in all these species, including four of the five lower algae species. The methylation of CHG requires the participation of *CMT3* which is also basically present in these studied species. Therefore, the maintenance of CG and CHG methylation may derive from the common ancestors of chlorophyta and embryophyta. The nucleosome remodeler *DDM1* which is essential for mediating methylation in the heterochromatin region, is basically present in all the studied species. The time of its occurrence is consistent with that of DNA methyltransferases (*MET1* and *CMT3*). There are 4 genes (*JMJ14*, *UBP26*, *HDA6* and *SUVH4*) involved in the histone modification process, they all are found in the algae. These genes are important components involved in histone modications, thereby they influence DNA methylation and can affect phenotypic traits such as flowering time and drought tolerance of plants [47–50].

For the DNA demethylation process, active demethylation is mediated by four types of DNA glycosylases (*DME*, *ROS1*, *DML2* and *DML3*). The previous studies showed that *DME* was found only in dicots [42], but our results indicated that *DME* and *ROS1* were present from algae to higher flowering plants. *DML2* can be seen only in dicots with a relatively small proportion (17%). An analysis of the distribution of *DML2* in different species showed that it was only present in cruciferous plants (Additional file 5: Table S2). *DML3* was present in dicots and monocots. These results showed that DNA demethylation might have originated from the common ancestor of chlorophyta and embryophyta, while *DML2* is limited in cruciferous plants and *DML3* is limited in dicots and monocots.

### The abundance and duplication of methylation-related genes

To explore how methylation-related genes evolved, we investigated the abundance and duplication patterns of methylation-related genes in different species. Firstly, we summarized the abundance of methylation-related genes in different species (Additional file 5: Table S2). A large number of gene expansion events were observed. In 28 diploid species, such as *Setaria italica*, *Brachypodium distachyon*, *Fraxinus excelsior*, *Malus domestica*, *Lotus japonicus*, *Glycine max*, *Populus trichocarpa* and *Citrus sinensis*, the number of genes containing multiple copies was significantly larger than that in the other 43 diploid species (ANOVA, *p* < 0.01) (Additional file 5: Table S2). Among the six polyploid species, including five tetraploids (*Panicum virgatum*, *Nicotiana tabacum*, *Gossypium hirsutum*, *Gossypium barbadense*, *Brassica napus*) and one hexaploid (*Triticum aestivum*), the number of methylation-related genes was normalized by dividing them at the diploid level. The result showed that the number of genes containing multiple copies was relatively larger in *P. virgatum*, *T. aestivum* and *B. napus* (ANOVA, p < 0.01) (Additional file 6: Table S3).

Gene absence is also observed for some methylation-related genes. In algae, there is a notable absence of methylation-related genes (Additional file 5: Table S2). There are 23 methylation-related genes that are not present in all five algae species. It is only found that 4 methylated-related genes, *JMJ14, UBP26, DDM1* and *DME*, were present in all 5 algae. The absence rate of methylation-related genes is much larger in advanced plants. In monocots, 1 to 10 genes were missing. In dicots, there are 48 species that had gene absence events. In the three *Arabidopsis* species, the methylation-related genes in *A. thaliana* and *A. lyrata* were conserved, while in the *A. halleri*, 4 genes (*RDM1*, *SUVH9*, *HDA6* and *NRPD/E2*) were absent. In *Solanum tuberosum*, the absence of genes was greatly pronounced, with 10 of the 33 genes were missing.

The expansion of methylation-related genes may be related to whole genome duplication (WGD) and tandem duplication. For example, the genome duplication event of *G. max* that occurred approximately 13–59 million years ago, contributed to the formation of multiple copies for nearly 75% of genes [51]. About 50 million years ago, there was a WGD event in *M. domestica*, which increased gene number significantly [52]. Two *IDN2* genes in *Gossypium raimondii*, *Gorai.010G201400* and *Gorai.010G201300*, were evolved from tandem duplication. Here, we analyzed the evolutionary patterns of methylation-related genes in *Oryza sativa*, *Solanum lycopersicum*, *Vitis vinifera*, *Amborella trichopoda*, *Cucumis sativus* and *Phaseolus vulgaris* (Additional file 7: Table S4). Through the analysis of WGD and tandem duplication information of these five species [53, 54], we found that both duplication patterns contributed to gene expansion in these species. Two copies of the *MET1* gene in *O. sativa* were evolved from WGD. In three copies of *AGO4* gene in *P. vulgaris*, *Phvul.008G206600* and *Phvul.006G021200* were evolved from WGD, *Phvul.008G206600* and *Phvul.008G206500* were evolved from tandem duplication. The similar phenomenon can also be found in *S. lycopersicum*, *V. vinifera*, *A. trichopoda* and *C. sativus*.

**Fig. 2:**
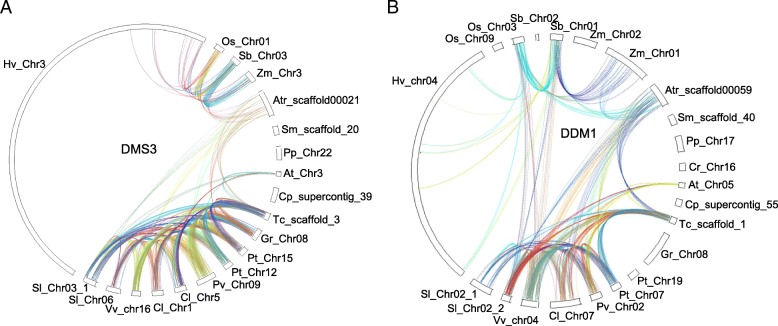
Collinear analysis of chromosome fragments containing 20 adjacent genes upstream and downstream of methylation-related genes. **a** The collinearity of DMS3 genes in different species. **b** The collinearity of DDM1 genes in different species. The species names are *Chlamydomonas reinhardtii* (Cr), *Physcomitrella patens* (Pp), *Selaginella moellendorffii* (Sm), *Amborella trichopoda* (Atr), *Zea mays* (Zm), *Sorghum bicolor* (Sb), *Oryza sativa* (Os), *Hordeum vulgare* (Hv), *Solanum lycopersicum* (Sl), *Vitis vinifera* (Vv), *Citrullus lanatus* (Cl), *Phaseolus vulgaris* (Pv), *Populus trichocarpa* (Pt), *Gossypium raimondii* (Gr), *Theobroma cacao* (Tc), *Carica papaya* (Cp), *Arabidopsis thaliana* (At)

We used 17 of 77 species for a gene collinearity analysis, including four early plants, four monocots and nine dicots. In each methylation process, we carried out a collinear analysis of some genes (Fig. 2 Additional file 1: Figure S1). A total of 10 methylation-related genes were analyzed. The collinearity of chromosomal regions which contained methylation-related genes indicated that these genes were the products of WGD or segment duplication. For *AGO4*, *DMS3* and other genes, some chromosomal segments had better collinearity in the same species. We also compared the collinearity between different species. There is no collinearity between the early plants and the monocots or dicots for most genes, and there is also no collinearity between monocots and dicots for most genes. In different early plant species, chromosomal collinearity is not observed. In monocots, the collinearity of *Zea mays*, *Sorghum bicolor*, and *Oryza sativa* is found for most genes. In dicots, the collinearity of most genes is observed, however, some genes in *A. thaliana*, *Citrullus lanatus*, *S. lycopersicum* and *Carica papaya* are in poor collinearity. These data suggest that genome evolution has an effect on disrupting chromosomal collinearity in different species in terms of methylation-related genes.

### The conservation and change of gene structures of methylation-related genes

To explore how gene structure has changed during evolution, we used 18 representative species for an exon-intron structure analysis. We started this analysis by examining exon-intron structure of these methylation-related genes at the species level (Additional file 8: Table S5). Generally, gene length and intron length varied largely (Additional file 8: Table S5A-B, S5G-H), but the exon number and exon length of most genes were conserved (Additional file 8: Table S5C-F). The correlation analysis indicated that the change of gene length was primarily due to the change of intron length (r = 0.79, *p* < 0.01). For example, in *A. trichopoda*, *Z. mays*, *A. thaliana* and *V. vinifera*, gene length and intron length of most methylation-related genes changed a lot, but the exon length varied little. Interestingly, in *C. reinhardtii*, *P. abies* and *C. papaya*, the gene length and intron length of most methylation-related genes varied a lot, but the exon length and exon number of some methylation-related genes also had some changes.

**Fig. 3:**
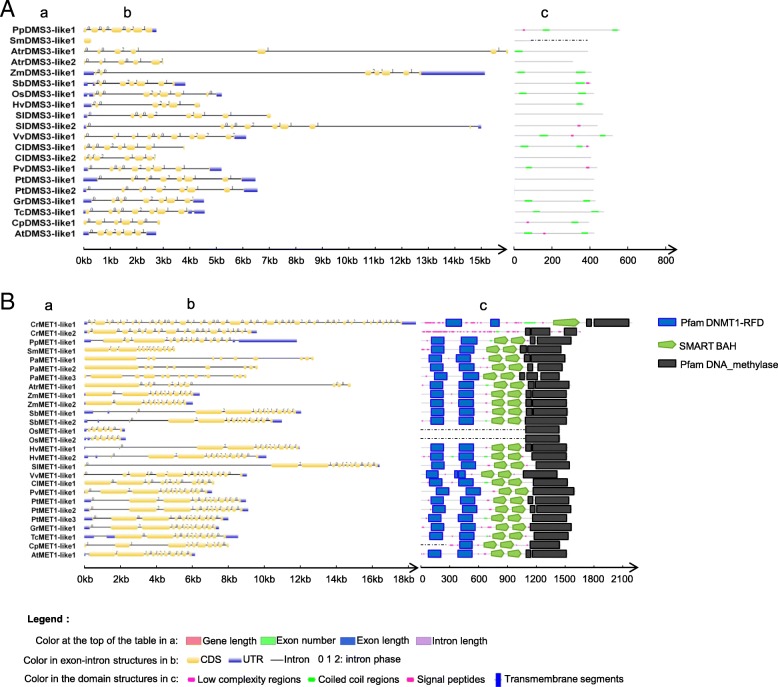
Exon-intron structures and domain structures. (A) Exon-intron structures and domain structures of DMS3. a: A summary of gene length, exon number, exon length and intron length of DMS3; b: The gene structure were shown by the online tool GSDS. c: The domain structures were shown by the online tool SMART. (B) Exon-intron structures and domain structures of DMS3. a: A summary of gene length, exon number, exon length and intron length of DMS3; b: The gene structure were shown by the online tool GSDS. c: The domain structures were shown by the online tool SMART. Color in the table shows: Yellow represents a large positive difference from the average trend; Green represents a large negative difference from the average trend. The species names are *Chlamydomonas reinhardtii* (Cr), *Physcomitrella patens* (Pp), *Selaginella moellendorffii* (Sm), *Amborella trichopoda* (Atr), *Zea mays* (Zm), *Sorghum bicolor* (Sb), *Oryza sativa* (Os), *Hordeum vulgare* (Hv), *Solanum lycopersicum* (Sl), *Vitis vinifera* (Vv), *Citrullus lanatus* (Cl), *Phaseolus vulgaris* (Pv), *Populus trichocarpa* (Pt), *Gossypium raimondii* (Gr), *Theobroma cacao* (Tc), *Carica papaya* (Cp), *Arabidopsis thaliana* (At)

Then, the exon-intron structure was studied at the functional domain level of protein sequences (Fig. 3 and Additional file 2: Figure S2). First, five genes were studied which were involved in de novo DNA methylation (Additional file 2: Figure S2A-E), including *RDR2, CLSY1, DCL3, HEN1* and *AGO4* in the process of Pol IV-dependent siRNA biogenesis. The exon-intron structure of *AGO4* was conserved, and the changes of exon-intron structure of the other four genes were large, mostly because of intron changes. The domain structure of these genes was basically conserved. In the Pol V-mediated de novo methylation (Fig. 3a, Additional file 2: Figure S2F-M), the changes in exon-intron structure of these genes were notable except for *KTF1*. The domain structure of *RDM1*, *DRD1*, *MORC6* and *KTF1* was highly conserved from early plants. It is found that *DMS3* only contains low complexity regions and coiled coil regions. *SUVH2/9* had SRA, PreSET and SET domains in spermatophyte, but both genes had only one of these three domains in early plants. In the chromatin alteration process (Additional file 2: Figure S2 N-O), the gene and intron length of *IDN2* and *IDP1* changed a lot, but their protein domains were highly conserved. In the subunits of RNA polymerases (Additional file 2: Figure S2 P-T), the changes of exon-intron structure for *NRPD1*, *NRPD/E4* and *NRPE5* were mainly because of intron changes. Their functional domains were conserved with key domains existing in the most ancestral species. We also found that in early plants (*P. abies* and *A. trichopoda*), the domain structure of some key genes changed greatly. A similar trend was observed in monocots *O. sativa* and *H. vulgare* and dicot *C. papaya*. In the process of de novo CHH methylation (Additional file 2: Figure S2U), the exon-intron structure of *CMT2* changed a lot. The *CMT2* in *C. papaya* was short and contained only a partial domain, and *CMT2* in *P. abies and H. vulgare* were short and did not contain domains that existed in other species.

Second, we investigated genes responsible for maintenance of DNA methylation (Fig. 3b, Additional file 2: Figure S2V). The exon-intron structure and domains of two genes (*MET1* and *CMT3*) were generally conserved. However, for two duplicated *MET1* genes in rice, only the DNA_methylase domain was found in each copy, and the protein sequences were short. In *C. reinhardtii*, *CMT3* contained some peptide segments with low-complexity, but lacked the BAH and CHROMO domains. In *P. abies*, these domains of *CMT3* were absent. The domains of *CMT3* in rice were also different from that in *Arabidopsis*. As for nucleosome remodeling factor *DDM1* (Additional file 2: Figure S2W), the exon-intron and domain structures were highly conserved in higher plants. In the histone modification process (Additional file 2: Figure S2X-Z, S2AA), the exon-intron structure of genes changed a lot, but their protein domains were highly conserved from early plants.

Finally, we investigated genes responsible for DNA demethylation (Additional file 2: Figure S2BB-DD). The exon-intron and domain structures of *DME*, *ROS1* and *DML3* were conserved. There were only a few exceptions. The exon length of *DME* in *C. reinhardtii* was long. For domain structures, *DME* in *C. reinhardtii* contained a large number of low-complexity regions in addition to the partial domain of *A. thaliana.* The protein sequences of *DME* and *ROS1* in *P. abies* were short and contained only a partial domain. *ROS1* in *C. papaya* was short and did not contain any domains.

In summary, the exon-intron structure and functional domains varied greatly in the early plants, but they were conserved for most genes in monocots and dicots. Of note is the observation that a few domains of some genes in monocots *H. vulgare* and *O. sativa* varied greatly, and the dicot species *C. papaya* had considerable changes in the domains of most genes. Genes with domain changes had notable changes in exons, which is consistent with the overall changes of the exon-intron structure at the species level (Additional file 8: Table S5).

### The phylogenetic relationship and purifying selection of methylation-related genes

**Fig. 4:**
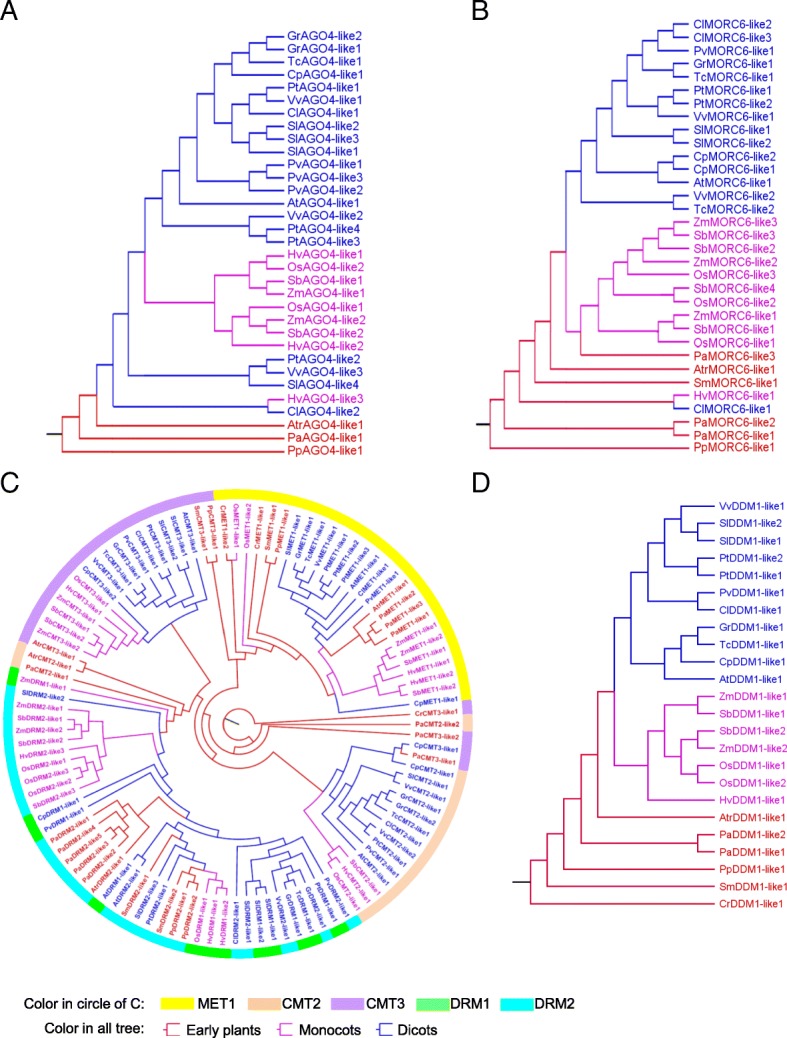
The evolutionary tree of methylation-related genes constructed by neighbor joining method in 18 species. **a** The evolutionary tree of AGO4 (ARGONAUTE 4) gene. **b** The evolutionary tree of MORC6 (MICRORCHIDIA 6) gene. **c** The evolutionary tree of methyltransferases. **d** The evolutionary tree of DDM1 (DECREASE IN DNA METHYLATION1) gene

The identification of genes in more than 77 species has led to investigate the origin of methylation pathways. Through exon-intron and domain structure analysis, we have known that although the length of genes varied greatly, but the functional structures were relatively conserved. Next, we sought to explore the phylogenetic relationship of methylation-related genes (Fig. 4 and Additional file 3: Figure S3).

At first, we studied de novo DNA methylation-related genes (Fig. 4a-b, Additional file 3: Figure S3A-O). The de novo DNA methylation process involves many genes, and a phylogenetic analysis categorized them into three groups, including early plants, dicots and monocots. A detailed analysis of these data showed that most methylation-related genes with multiple homologs tended to cluster in different branches in monocots, however most of these genes tended to gather in a single branch in dicots. We hypothesize that methylation-related genes in monocots may have been duplicated prior to the divergence of monocots and dicots, and possibly most of the genes in dicots tend to duplicate after this divergence event. The *MORC6* genes in monocots are clustered into three branches, while the *MORC6* genes in dicots *C. lanatus*, *P. trichocarpa*, *C. papaya* and *S. lycopersicum* were clustered in one branch. A similar observation was obtained for *AGO4*, *DCL3*, *DRD1* and *IDP1*. Because both *NRPD1* and *NRPE1* came from *NRPB1* and the sequence similarity between *NRPD1* and *NRPE1* was very high, we combined *NRPD1* and *NRPE1* subunits together to explore their evolutionary relationship. In early plants, *NRPD1* and *NRPE1* were clustered together, but they were clustered into two branches in angiosperms, which suggested that *NRPD1* and *NRPE1* might be divergent from the ancestors of monocots and dicots.

We then studied those genes responsible for maintenance of DNA methylation (Fig. 4c). In order to explore a full phylogenetic relationship of these methyltransferases, we constructed an evolutionary tree by combining them together with those responsible for de novo methylation. It can be seen that *MET1*, *CMT2* and *CMT3* were well clustered together, and the *DRM1* and *DRM2* were clustered into two branches in monocots but clustered together in dicots and early plants. As for the nucleosome remodeling factor DDM1, we found that all the *DDM1* genes in different species were clustered into three branches (Fig. 4d). In the histone modification process (Additional file 3: Figure S3P-S), with some exceptions, *JMJ14*, *UBP26*, *HDA6* and *SUVH4* are basically clustered into three branches.

Finally, we analyzed demethylation-related genes. In the monocots and dicots, most DNA glycosylases were clustered separately into the *DME* branch, the *ROS1* branch or the *DML3* branch, but they were clustered together in the early plants (Additional file 3: Figure S3T). This result suggests that the ancestral demethylation-related genes might be differentiated into different demethylation-related genes after the divergence of monocots and dicots.

**Fig. 5:**
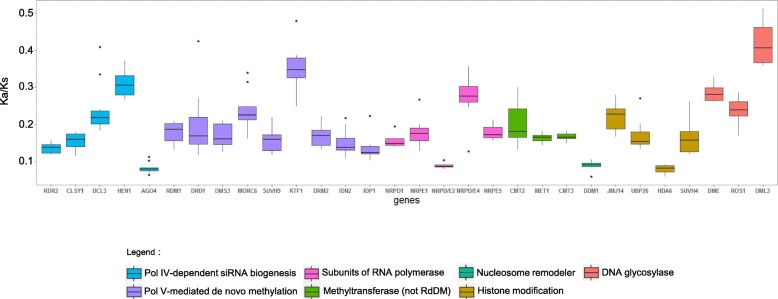
The distribution of Ka/Ks values of methylation-related genes. The Ka and Ks values were computed using the PAML program. The Ka/Ks value less than 1 indicates that the gene is under negative selection

In addition, we investigated the selection pressure of methylation-related genes by calculating the ratio of nonsynonymous substitutions per nonsynonymous site (Ka) to synonymous substitutions per synonymous site (Ks) (omega) for each homologous gene pair in nine dicots, with reference to genes in *A. thaliana*. We found that all genes were under negative selection (Fig. 5). This indicated that these genes were highly conserved during the evolution of dicotyledonous plants, which also reflected the importance of DNA methylation for dicotyledonous plants.

### Divergent expression patterns of methylation-related genes in different species

**Fig. 6:**
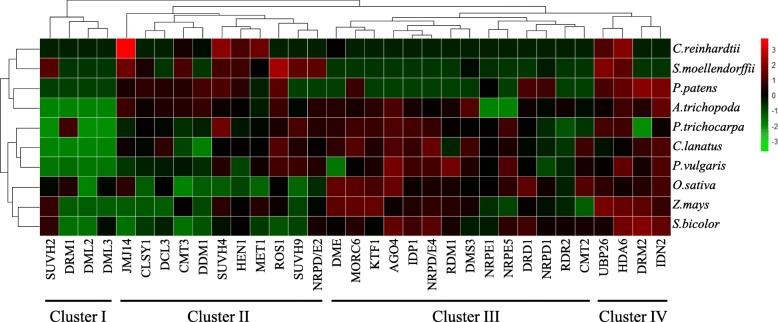
Heatmap of expression of methylation-related genes. Heatmap of expression of 33 methylation-related genes in 10 species. In three early plants - *C. reinhardtii*, *P. patens* and *S. moellendorffii*, the whole plant was used; in basal angiosperm *A.trichopoda*, 3 dicots and 3 monots, the leaf tissue was used. The heatmap was presented using the R package pheatmap. 3 and − 3 represents the maximum and minimum values of normalized gene expression level, respectively. The red color in the heatmap shows high expression and the green color in the heatmap shows low expression

On the basis of tracing evolution history of methylation-related genes from early plants to higher plants, we next aim to explore how the methylation-related genes have evolved at the expression level in different species. We selected 10 species for analysis of expression patterns, including algae, bryophyte, fern, basal angiosperm, dicot and monocot. In three early plants - *C. reinhardtii*, *P. patens* and *S. moellendorffii*, samples from the whole plant were used; in basal angiosperm *A.trichopoda*, 3 dicots and 3 monots, data from the leaf tissue were used. The normalized expression level of gene (TPM) was shown in the form of heatmap (Fig. 6).

First of all, we compared the expression patterns of methylation-related genes at the species level. We can see that the early plants, monocots and dicots were clustered into one branch, respectively. This indicated that the expression patterns of methylation-related genes might have changed during the evolution from early plants to higher plants, as well as during the differentiation of monocots and dicots. In the evolution of these genes, there were changes not only in duplication pattern and gene structure, but also in gene expression patterns, which provides an idea for us to study the origin and evolution of other genes. From the gene level, we can see that the expression patterns of these genes can be clustered into four categories. Here, we mainly analyzed the expression pattern of angiosperms. In cluster III and IV, the expression levels were high, which may reflect the conservation of gene expression in species evolution, and also explain the importance of these genes in plant growth and development. *UBP26* and *HDA6* are important components involved in histone modications, thereby influence DNA methylation and affect phenotypic traits [47–50]. *DRM2* is the key gene in RdDM pathway [5, 12–16]. In cluster I and cluster II, the expression levels of these genes were low.

## Discussion

DNA methylation is common in the entire eukaryotic species and is essential for plant growth and development, which can cause gene and transposon silencing and genomic imprinting, and regulate other biological processes [1, 5–10]. The methylation process has been systematically studied in *A. thaliana*. In this study, we focus on the origin and evolution of methylation-related genes in the whole plant kingdom.

### The origin of methylation-related genes

The de novo DNA methylation process may be established from the common ancestor of bryophyte and tracheophyta after divergence of chlorophyta and embryophyta (Fig. 1). The maintenance of CG and CHG methylation can be derived from the common ancestor of chlorophyta and embrypphyta. Hypothetically, DNA methylation may be established firstly, and then maintenance of methylation starts to work on the basis of the established methylation, i.e., de novo methylation may appear earlier than maintenance. But, in our study, genes associated with the maintenance of CG and CHG methylation appeared earlier than most genes for de novo methylation. DNA demethylation may have originated from the common ancestor of chlorophyta and embryophyta, which is similar to the maintenance of CG and CHG methylation. But *DML2* was identified in cruciferous plants, which may come from the evolution of the *DME* after crucifer differentiation, and *DML3* was limited in mesangiospermae, which may come from *DME* after divergence of mesangiospermae and basal angiosperm. In other words, the mesangiospermae and the cruciferous plants may have also evolved new demethylation processes which depends on *DML3* and *DML2*. It requires further investigation to know whether the presence of these two enzymes means that the demethylation process appears in cruciferae superior to other species. The symmetric methylation (CG and CHG) can be maintained by MET1 and CMT2 which appeared earlier, and most de novo methylation-related genes appeared late. And there is no systematic comparison of the conservation of symmetric methylation with other methylation-related genes. In the following studies, we can explore whether symmetric methylation can be maintained easier and is therefore evolutionarily conserved.

Histone modification interacts with DNA methylation in plants. H3K9me2 is a silent mark, and 70% of RdDM targets are modified by H3K9me2 [55]. SUVH4 maintains H3K9me2 and thereby contributes to DNA methylation. Acetylation and ubiquization of H2B and H3K4me are active markers. The active marks of RdDM targets need to be removed in order to maintain DNA methylation and the silencing state caused by DNA methylation [20–23]. These four histone modifications evolved earlier than genes for de novo methylation, indicating that histone modification might affect the growth and development of plants in some other ways before de novo methylation.

### The absence of methylation-related genes

The gene absence was common in plants (Additional file 5: Table S2). Of the 77 species, 73 species had gene absence. The CMT3 genes were reported to have been lost in two angiosperms (*Eutrema salsugineum* and *Conringia planisiliqua*) [56]. In algae, the widespread gene absence may be due to their later evolution; in higher plants, such as monocots and dicots, these common absence events pointed to the possibility that epigenetic genes might be lost or gained in individual lineages of angiosperms. The effects of the absence of these genes on the apparent modification of plants need to be further explored.

### The exon-intron structure and the methylation level of species

In this study, we have found that most species exhibited conserved exon length and number for methylation-related genes. Simultaneously, the domain sequences which were closely related to the function of genes were also highly conserved. In *C. papaya*, the exon-intron structure and domain changed greatly. One reason for this may be the relatively poor quality of the genome assembly, and the other is that these genes indeed have evolved dramatically during evolution. The exon-intron structure and domains of a number of genes changed greatly in *O. sativa*, such as *MET1* (Fig. 3). MET1 in rice had only the DNA methylase domain and had no BAH and DNMT1-RFD domains, but MET1 in the other species contained all three types of domains. Based on the whole genome methylation data in *O. sativa* and other species, we could see that CG methylation content in *O. sativa* was higher than *A. thaliana*, *T. cacao* and *V. vinifera* [57], which indicated that the change in MET1 did not result in the overall decrease of CG methylation level in *O. sativa*. The reason why MET1 lacked the key domain and the CG methylation content remained at a high level requires further exploration.

## Conclusions

DNA methylation is one of the most common modifications in plants, which involves the dynamic balance of de novo methylation, maintenance methylation and demethylation. The origin time of different methylation-related genes is not consistent, which reflects the evolutionary complexity of DNA methylation in plants. The conservation in domain structure of gene sequences and negative selection of these genes reflect the conservation of the methylation process, which points to the functional importance of DNA methylation to plants.

## Methods

### Identification of methylation-related genes

The methylation-related genes in *A. thaliana* were retrieved from the TAIR (www.arabidopsis.org/) database. The protein sequences and coding sequences (CDS) of the other species, as well as the General Feature Format 3 (GFF3) and annotation files were downloaded from the websites shown in Additional file 9: Table S6. The protein sequence of methylation-related genes in *A. thaliana* were used as queries to identify homologous genes in the other 76 species using blastp program (sequence coverage > 30%, identity > 30% and E-value cutoff 1e-3, other parameters are default). At the same time, each candidate homologous gene was manually checked in combination with annotation file of each species. The partial genes that might result from un-complete genome assembly were excluded manually after sequence alignment. The species taxonomy tree was modified according to the information in the Taxonomy database of NCBI (https://www.ncbi.nlm.nih.gov/taxonomy/).

### Collinearity analysis

The chromosome location of methylation-related genes in each genome was obtained according to the GFF3 file. When homologous methylation-related genes were located in the same chromosome with no more than one intervening gene, these genes were defined as tandem duplication genes. We used the method as described in Maher et al. to identify large-scale duplication events [58]. If two genes were located in the same duplication block, the protein sequences on their flanks were highly similar at the amino acid level. Therefore, we located the methylation-related genes on the genome and used this location as the initial anchor site to obtain 20 protein-coding genes upstream and downstream of each site respectively [58]. This chromosomal regions containing 41 protein-coding genes were selected for collinearity analysis using MCScanX (blastp E-value cutoff 1e-10) [59].

### The exon-intron and domain structure analysis

The exon-intron structure information of methylation-related genes in every species was extracted from the GFF3 file using an in-house Perl script. Gene names used in gene structure analysis and phylogenetic analysis were shown in Additional file 10: Table S7. When multiple homologous genes in other species corresponded to one methylation-related gene in *A. thaliana*, the gene with the longest length was used to calculate the gene length, exon number, exon length and intron length. The diagrams of the exon-intron structure were drawn using the online tool GSDS (http://gsds.cbi.pku.edu.cn/). The protein domain structures were drawn using the online tool SMART (http://smart.embl-heidelberg.de/). The SMART and Pfam databases were used for domain prediction. In the exon-intron structure, the gene length values were averaged after removing two extreme values. When the gene length is more than 1.5-fold of the average value, the length is judged to be long. When the gene length is less than half of the average value, the length is judged to be short. These two values are called extreme values. When the value is between 0.5 and 1.5-fold of the average value, the length is judged to be moderate. Exon number, exon length and intron length are treated similarly.

### Phylogenetic analysis

The protein sequences of all methylation-related genes were aligned using Clustal X (version 2.0) [60]. Phylogenetic analysis was conducted using PHYLIP software with the neighbor-joining (NJ) method (bootstrap value 1000) [61]. The Figtree (version 1.4.3) software was used to display the phylogenetic tree.

### The estimation of selection pressure of methylation-related genes

Clustal X (v2.0) was used to conduct multiple alignment of protein sequences of methylation-related genes, which was guided by PAL2NAL software with the nogap parameter [62]. The yn00 procedure in PAML package was used to calculate the ratio of nonsynonymous substitutions per nonsynonymous site (Ka) to synonymous substitutions per synonymous site (Ks) for each homologous gene pair [63]. According to the definition of Ka/Ks, values less than one represent negative or purifying selection, while values greater than one represent positive selection. The saturation effect was ruled out by removing gene pairs with Ks > 2.5.

### The expression analysis of methylation-related genes

The RNA-Seq data in different species were collected from the NCBI Sequence Read Archive (SRA) database (Additional file 11: Table S8). In three early plants - *C. reinhardtii*, *P. patens* and *S. moellendorffii*, the whole plant was used; in basal angiosperm *A.trichopoda*, 3 dicots and 3 monots, the leaf tissue was used. Firstly, these raw reads were trimmed using trimmomatic-0.32 software [64]. Then, the clean data were mapped against the genomes using HISAT2 software (version 2.1.0) [65]. Finally, the StringTie software (version 1.3.4) was used to calculate the expression level for each gene normalized as ​Transcripts Per Million (TPM) [66]. When a methylation-related gene was absent in a species, the gene can not be transcribed, we recorded the expression of the gene as 0; When there are multiple homologous copies in a species, the sum of the expression of homologous copies is recorded as the expression level in a species. The normalized expression level of gene (TPM) was shown in the form of heatmap using the R package pheatmap.

## Additional files

Additional file 1: **Figure S1.** Collinear analysis of chromosome fragments containing 20 adjacent genes upstream and downstream of methylation-related genes. (A) The collinearity of *RDR2* genes in different species. (B) The collinearity of *AGO4* genes in different species. (C) The collinearity of *MORC6* genes in different species. (D) The collinearity of *DRM2* genes in different species. (E) The collinearity of *UBP26* genes in different species. (F) The collinearity of *NRPD1* genes in different species. (G) The collinearity of *NRPE5* genes in different species. (H) The collinearity of *MET1* genes in different species. The species name are *Chlamydomonas reinhardtii* (Cr), *Physcomitrella patens* (Pp), *Selaginella moellendorffii* (Sm), *Amborella trichopoda* (Atr), *Zea mays* (Zm), *Sorghum bicolor* (Sb), *Oryza sativa* (Os), *Hordeum vulgare* (Hv), *Solanum lycopersicum* (Sl), *Vitis vinifera* (Vv), *Citrullus lanatus* (Cl), *Phaseolus vulgaris* (Pv), *Populus trichocarpa* (Pt), *Gossypium raimondii* (Gr), *Theobroma cacao* (Tc), *Carica papaya* (Cp), *Arabidopsis thaliana* (At). (PDF 584 kb)

Additional file 2: **Figure S2.** Exon-intron structures and domain structures. (A-DD) Exon-intron structures and domain structures of *RDR2, CLSY1, DCL3, HEN1, AGO4, RDM1, DRD1, MORC6, SUVH2, SUVH9, KTF1, DRM1, DRM2, JMJ14, UBP26, HDA6, SUVH4, IDN2, IDP1, NRPD1, NRPE1, NRPD/E2, NRPD/E4, NRPE5, CMT2, CMT3, DDM,* DME, ROS1 and *DML3*. a: A summary of gene length, exon number, exon length and intron length of gene. The green and yellow in the table represent genes with extreme values. b: The gene structure was shown by the online tool GSDS. c: The domain structures were shown by the online tool SMART. The species name are *Chlamydomonas reinhardtii* (Cr), *Physcomitrella patens* (Pp), *Selaginella moellendorffii* (Sm), *Amborella trichopoda* (Atr), *Zea mays* (Zm), *Sorghum bicolor* (Sb), *Oryza sativa* (Os), *Hordeum vulgare* (Hv), *Solanum lycopersicum* (Sl), *Vitis vinifera* (Vv), *Citrullus lanatus* (Cl), *Phaseolus vulgaris* (Pv), *Populus trichocarpa* (Pt), *Gossypium raimondii* (Gr), *Theobroma cacao* (Tc), *Carica papaya* (Cp), *Arabidopsis thaliana* (At). (PDF 4727 kb)

Additional file 3: **Figure S3.** The phylogenetic tree of methylation-related genes constructed by a neighbor-joining method in 18 species. (A-S) The evolutionary tree of RDR2, DCL3, CLSY1, HEN1, RDM1, DRD1, DMS3, SUVH2/9, KTF1, JMJ14, UBP26, HDA6, SUVH4, IDN2, IDP1, NRPD1_NRPE1, NRPD/E2, NRPD/E4, NRPE5,, and DME_ROS1_DML3 genes. (PDF 824 kb)

Additional file 4: **Table S1.** The gene ID of methylation-related genes in 77 species. (XLSX 13399 kb)

Additional file 5: **Table S2.** In 77 species, the number of methylation-related genes, the number of single-copy genes, the number of multi-copy genes, the number of missing genes, and the chromosome ploidy of the sequenced species. (XLSX 58 kb)

Additional file 6: **Table S3.** In six polyploid plants, the normalized number of methylation-related genes, the normalized number of single-copy genes, the normalized number of multi-copy genes, the normalized number of missing genes, and the chromosome ploidy of the sequenced species. (XLSX 15 kb)

Additional file 7: **Table S4.** A summary of the duplication models of some methylation-related genes in five species. (XLSX 44 kb)

Additional file 8: **Table S5.** The gene length, exon number, exon length, intron length of methylation-related genes in 18 species. A: Gene length of methylation-related genes in 18 species. B: The number of genes with extreme values in gene length. C: Exon number of methylation-related genes in 18 species. D: The number of genes with extreme values in exon number. E: Gene length of methylation-related genes in 18 species. F: The number of genes with extreme values in exon length. G: Intron length of methylation-related genes in 18 species. H: The number of genes with extreme values in intron length. (XLSX 70 kb)

Additional file 9: **Table S6.** Summary of 77 sequenced genomes used for identification of methylation-related genes. (XLSX 15 kb)

Additional file 10: **Table S7.** Gene names used in gene structure analysis and phylogenetic analysis. (XLSX 72 kb)

Additional file 11: **Table S8.** The SRA accession number of RNA-Seq data used in this study. (XLSX 31 kb)

## Data Availability

All data analysed during this study are included in this published article and its supplementary information files.

## Abbreviations

CDS: Coding sequences
GFF3: General Feature Format 3
Ka: nonsynonymous substitutions per nonsynonymous site
Ks: synonymous substitutions per synonymous site
RdDM: RNA-directed DNA methylation
siRNAs: small interfering RNAs
WGD: Whole genome duplication
