# Supplementary figures and images for: Tracing the origin and evolution history of methylation-related genes in plants
Source: BMC Plant Biol. 2019 Jul 12;19:307. doi: 10.1186/s12870-019-1923-7 (PMC6624907; doi:10.1186/s12870-019-1923-7)

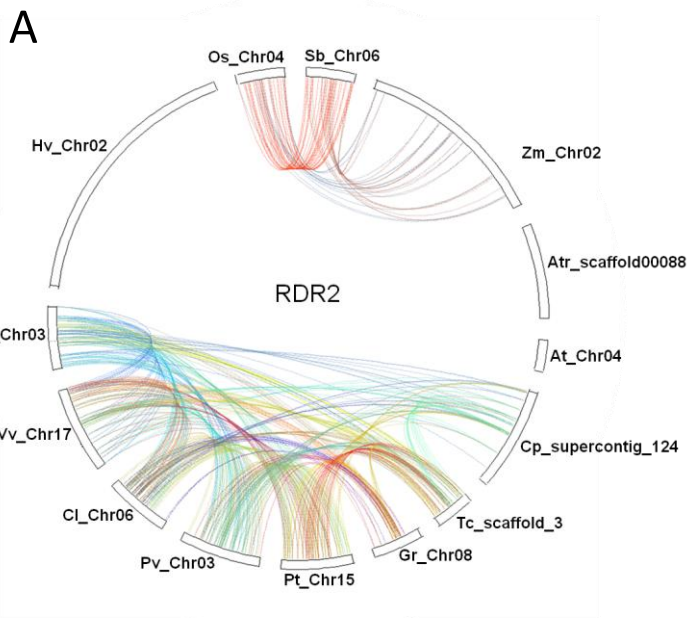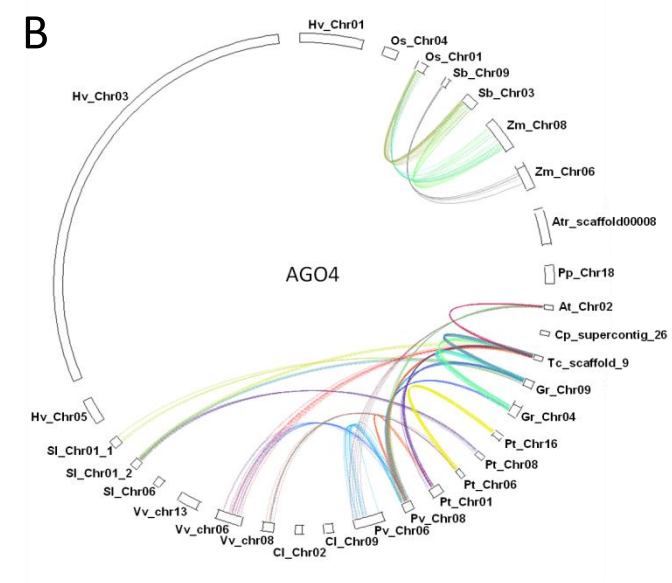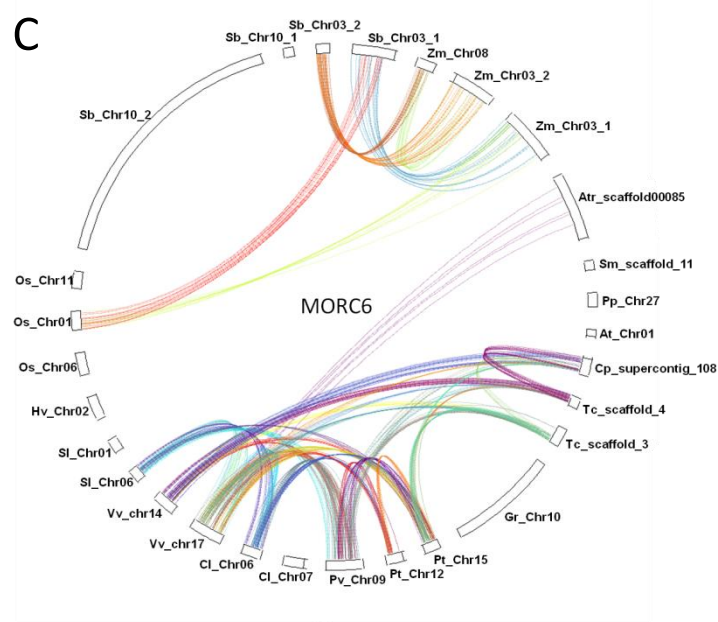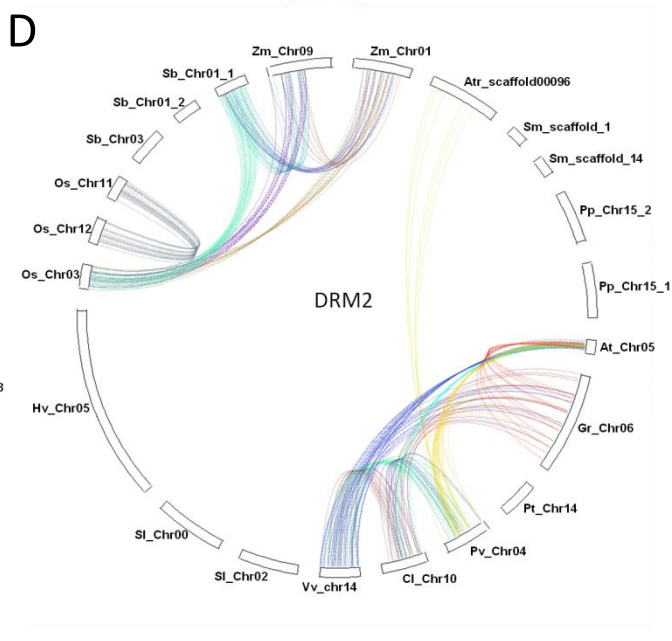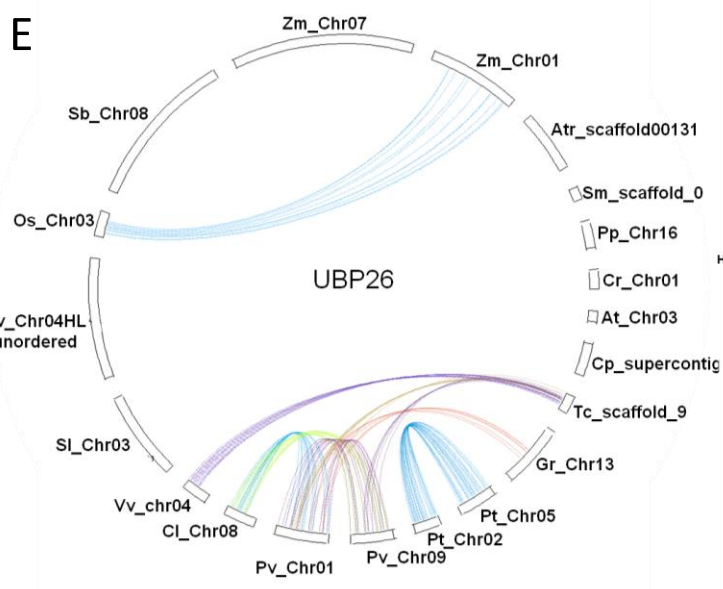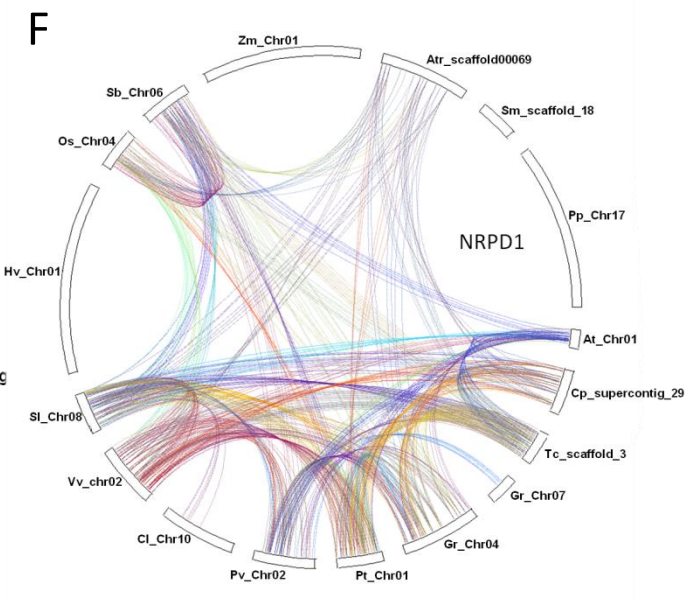

G

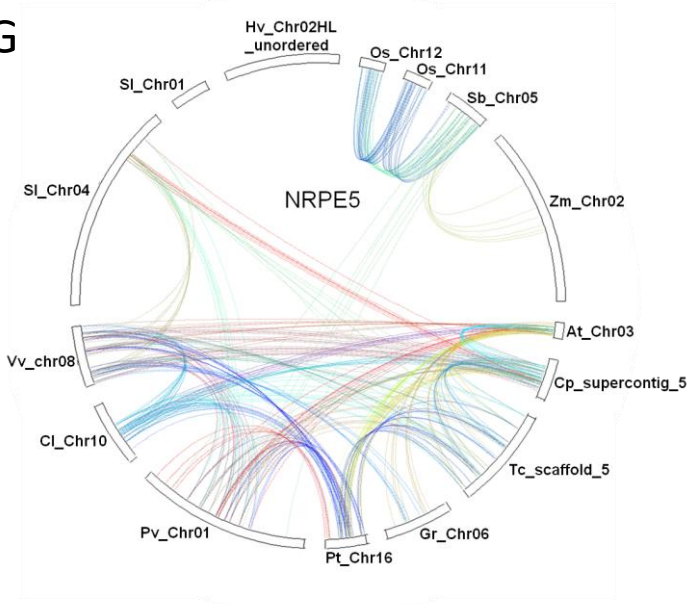

H

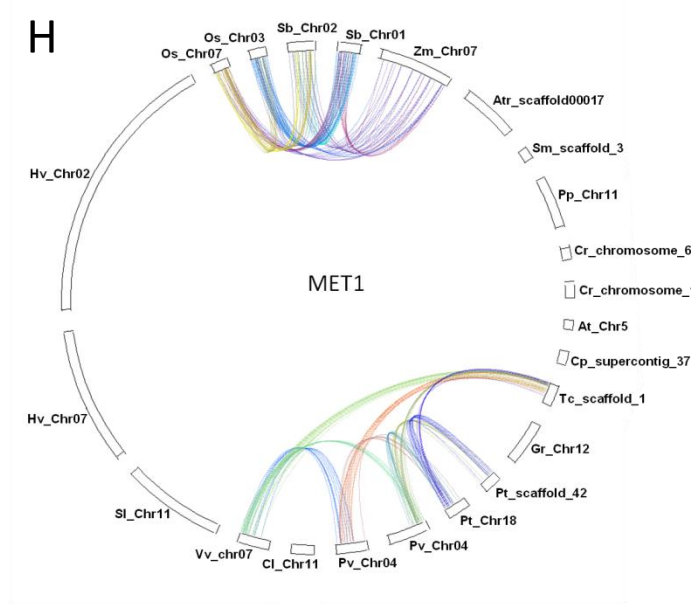

Supplement: Supplementary file 1 — Figure S1. Collinear analysis of chromosome fragments containing 20 adjacent genes upstream and downstream of methylation-related genes. (A) The collinearity of RDR2 genes in different species. (B) The collinearity of AGO4 genes in different species. (C) The collinearity of MORC6 genes in different species. (D) The collinearity of DRM2 genes in different species. (E) The collinearity of UBP26 genes in different species. (F) The collinearity of NRPD1 genes in different species. (G) The collinearity of NRPE5 genes in different species. (H) The collinearity of MET1 genes in different species. The species name are Chlamydomonas reinhardtii (Cr), Physcomitrella patens (Pp), Selaginella moellendorffii (Sm), Amborella trichopoda (Atr), Zea mays (Zm), Sorghum bicolor (Sb), Oryza sativa (Os), Hordeum vulgare (Hv), Solanum lycopersicum (Sl), Vitis vinifera (Vv), Citrullus lanatus (Cl), Phaseolus vulgaris (Pv), Populus trichocarpa (Pt), Gossypium raimondii (Gr), Theobroma cacao (Tc), Carica papaya (Cp), Arabidopsis thaliana (At). (PDF 584 kb) [file 12870_2019_1923_MOESM1_ESM.pdf]

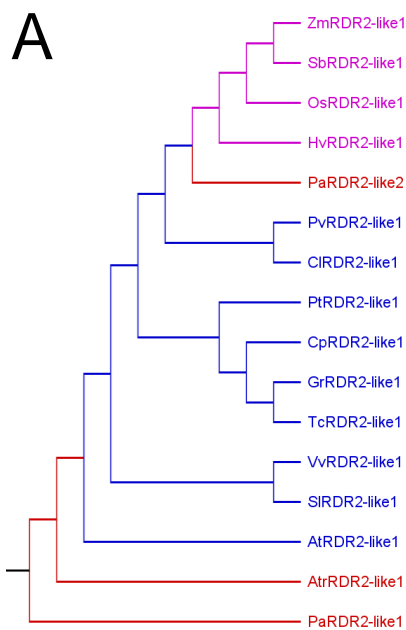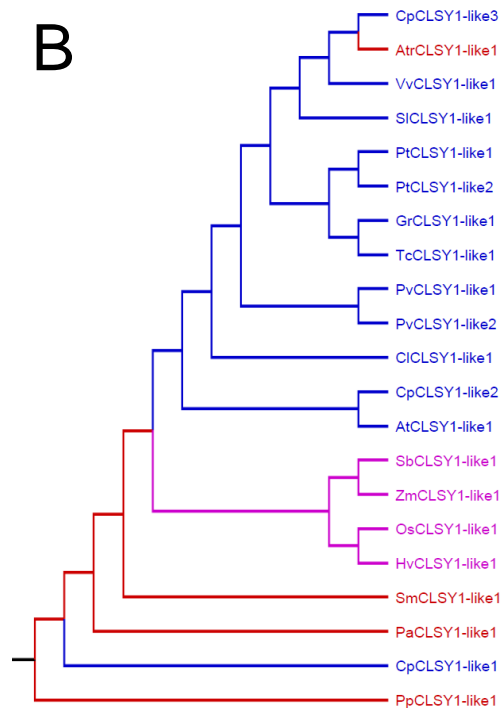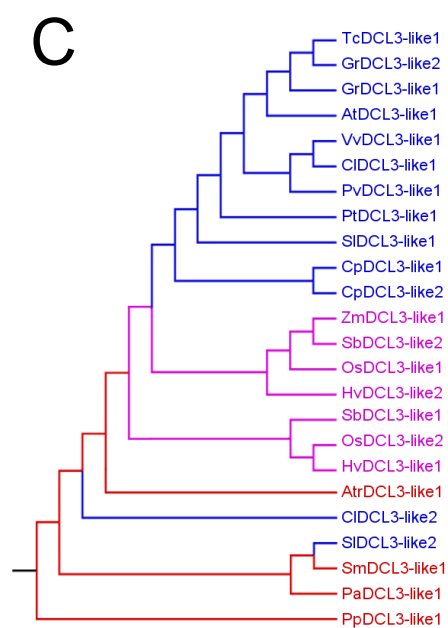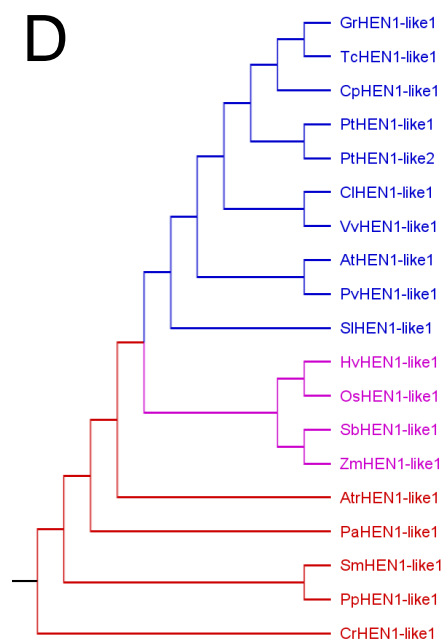

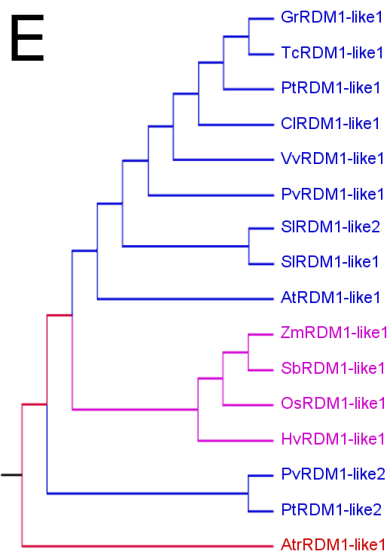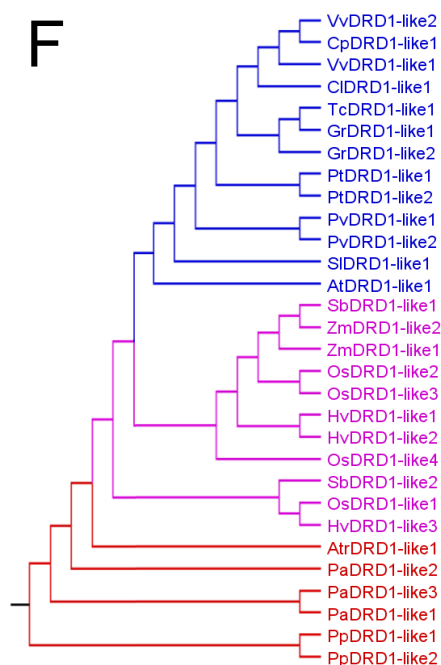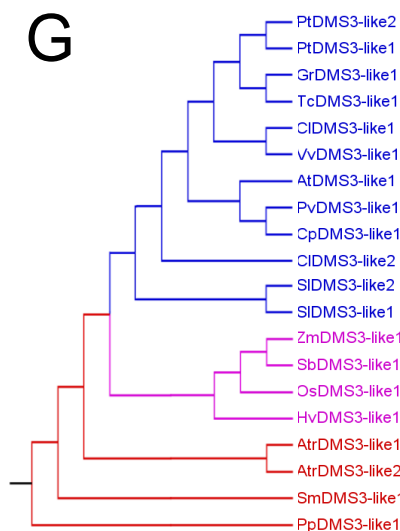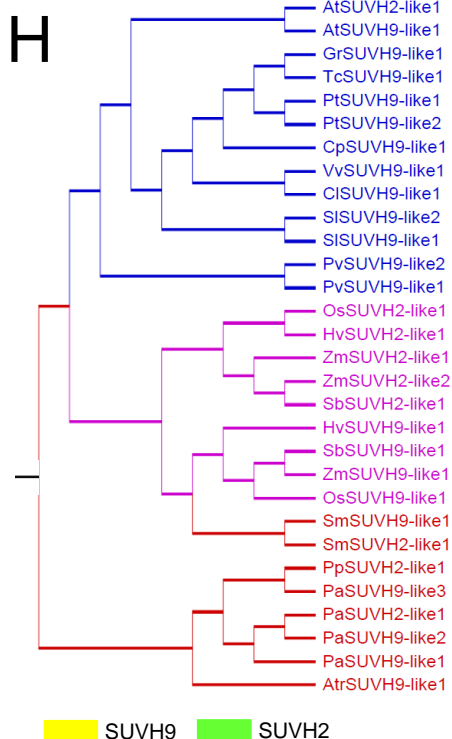

I

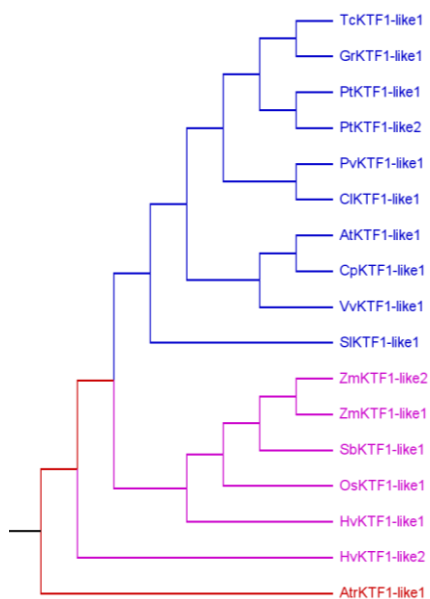

J

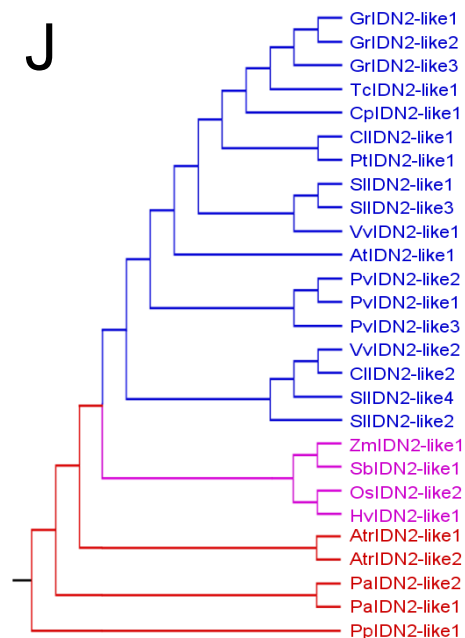

K

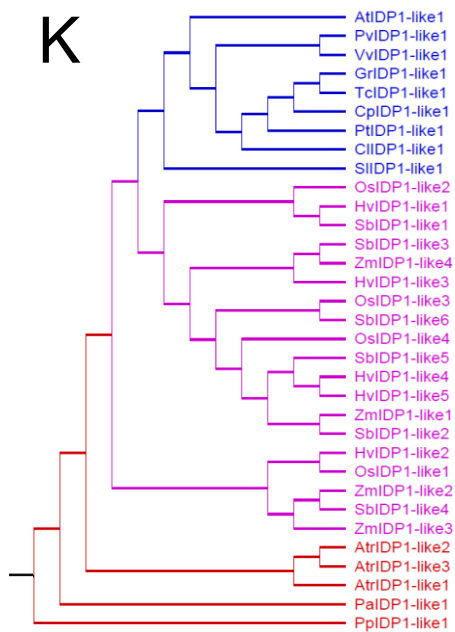

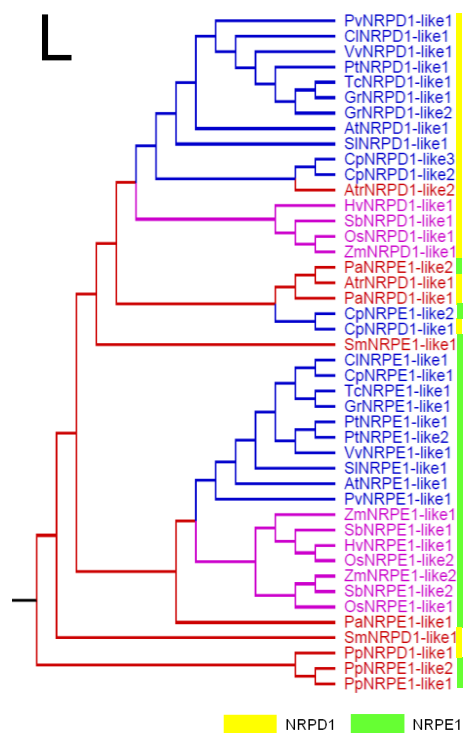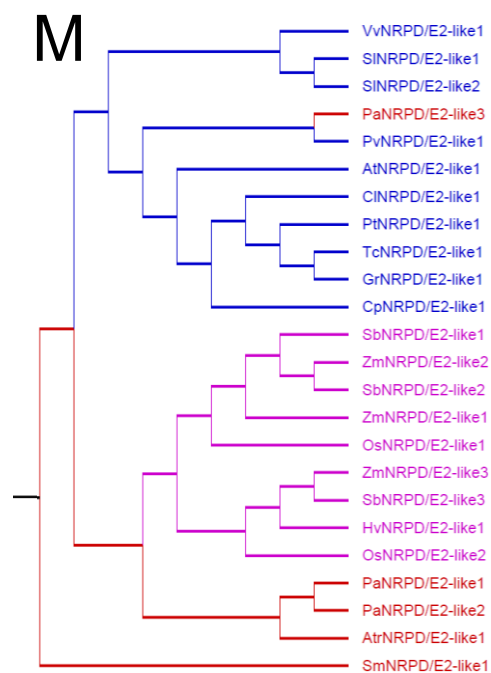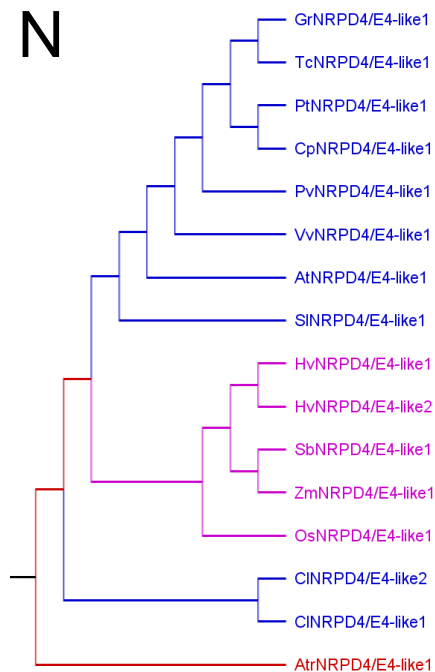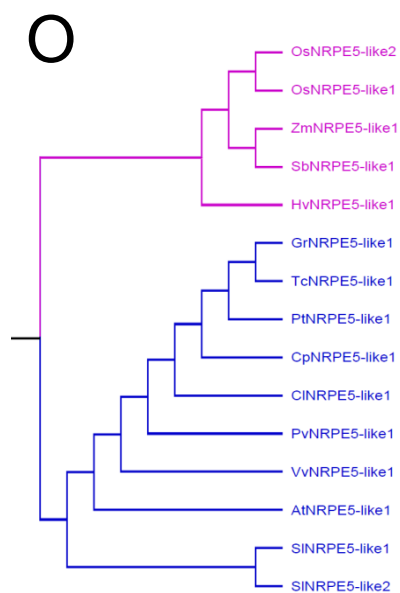

P

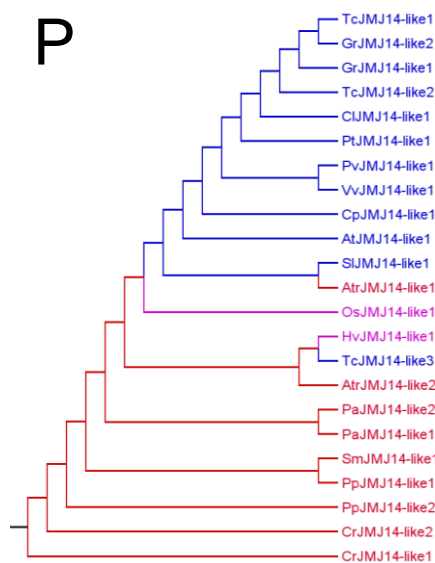

Q

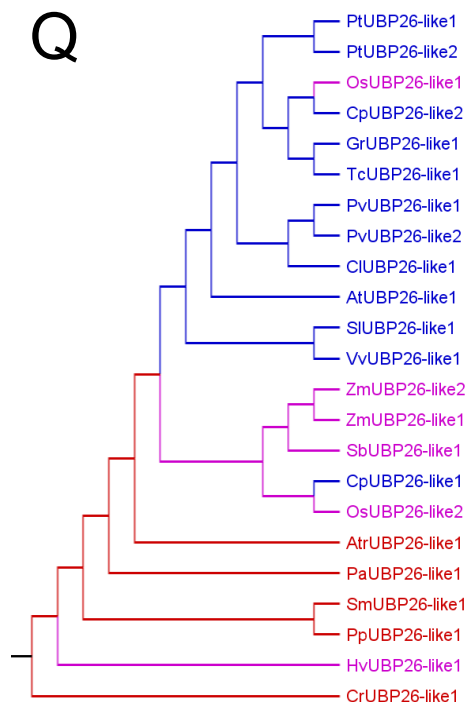

R

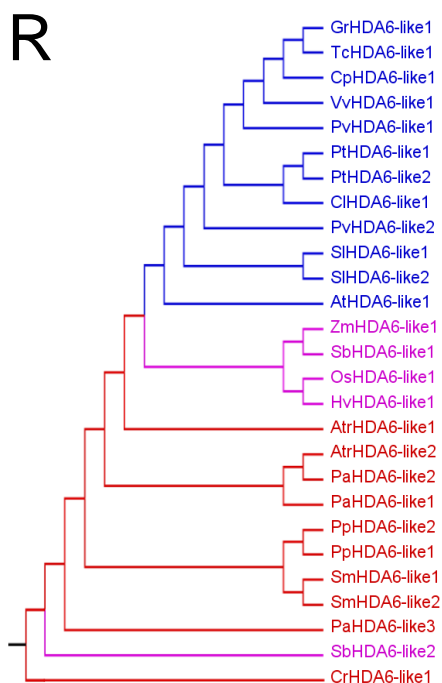

S

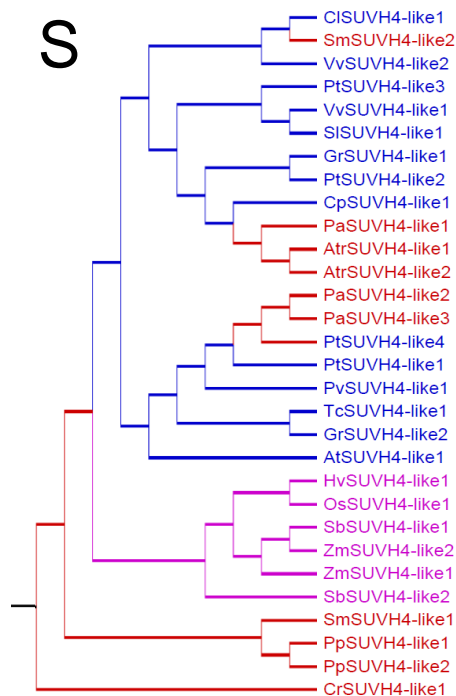

T

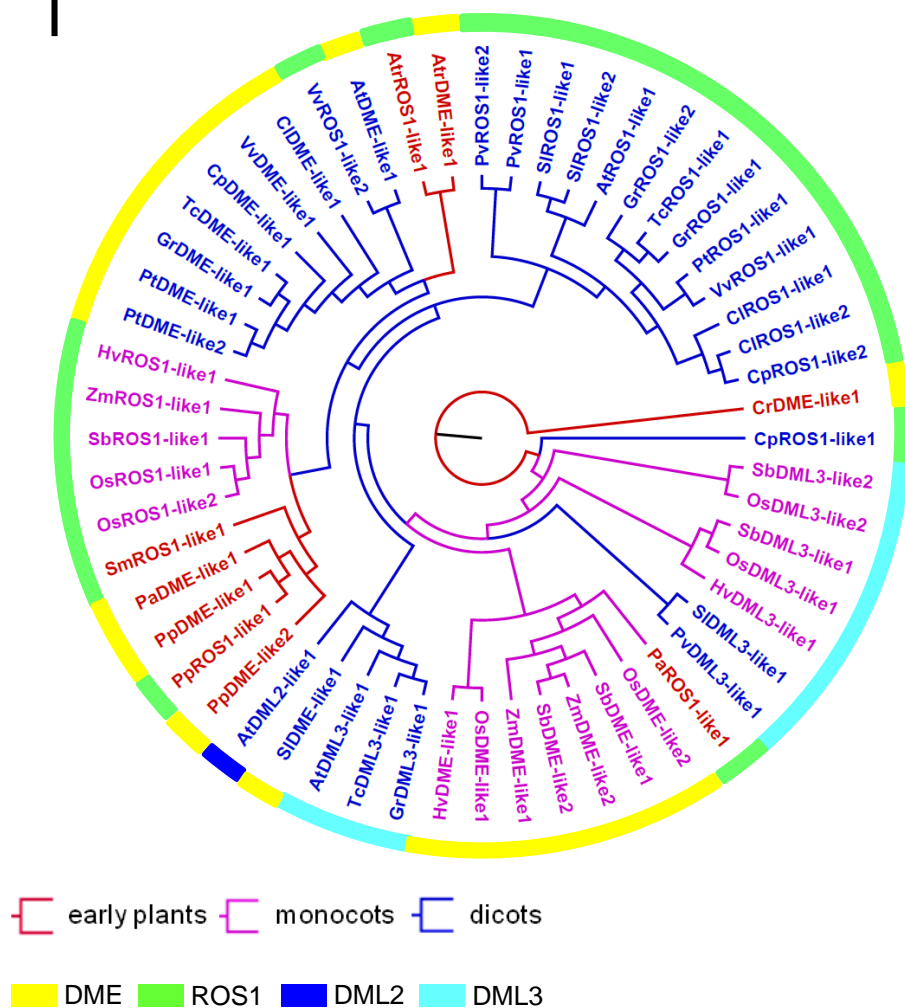

Supplement: Supplementary file 3 — Figure S3. The phylogenetic tree of methylation-related genes constructed by a neighbor-joining method in 18 species. (A-S) The evolutionary tree of RDR2, DCL3, CLSY1, HEN1, RDM1, DRD1, DMS3, SUVH2/9, KTF1, JMJ14, UBP26, HDA6, SUVH4, IDN2, IDP1, NRPD1_NRPE1, NRPD/E2, NRPD/E4, NRPE5,, and DME_ROS1_DML3 genes. (PDF 824 kb) [file 12870_2019_1923_MOESM3_ESM.pdf]
