# Supplementary material for: Tracing the origin and evolution history of methylation-related genes in plants
Source: BMC Plant Biol. 2019 Jul 12;19:307. doi: 10.1186/s12870-019-1923-7 (PMC6624907; doi:10.1186/s12870-019-1923-7)

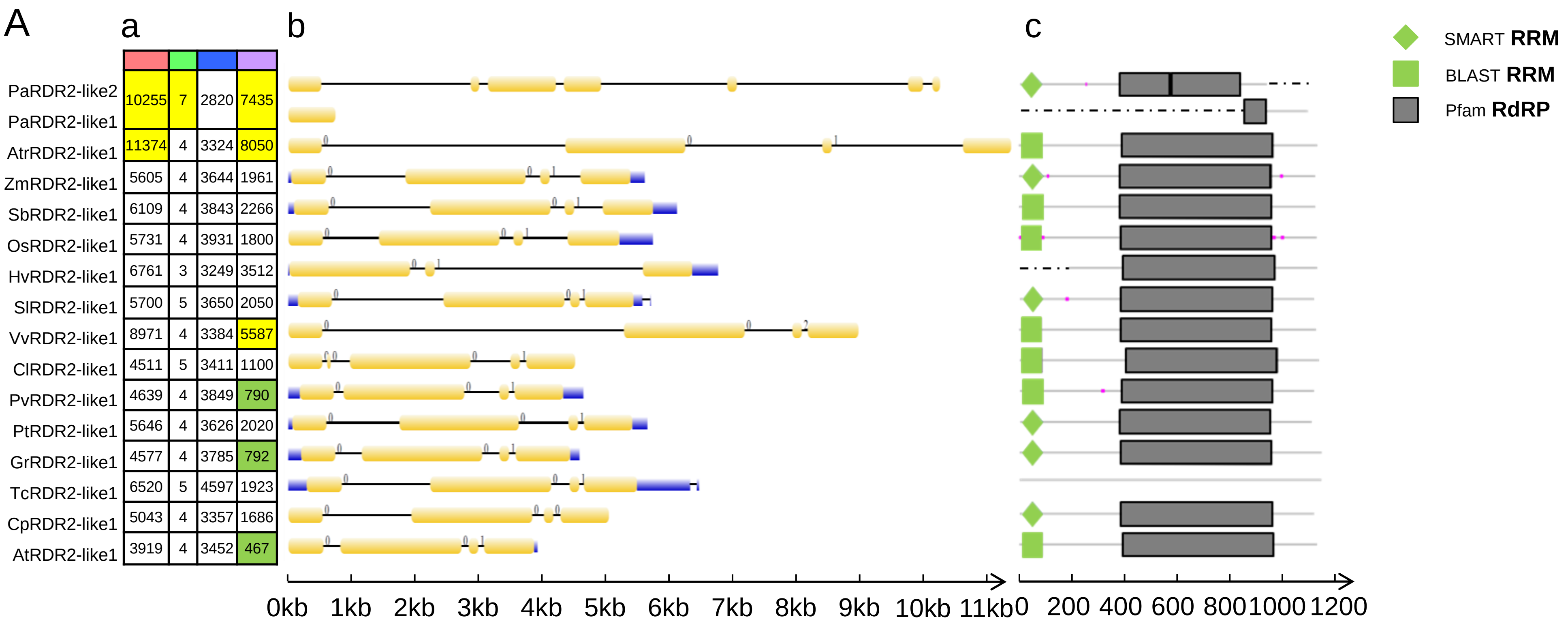

Legend:

Color at the top of the table in a: Gene length Exon number Exon length Intron length

Color in exons-introns structures in b: CDS UTR — Intron 0 1 2: intron phase

Color in the domain structures in c: Low complexity region Coiled coil regions Signal peptides Transmembrane segments

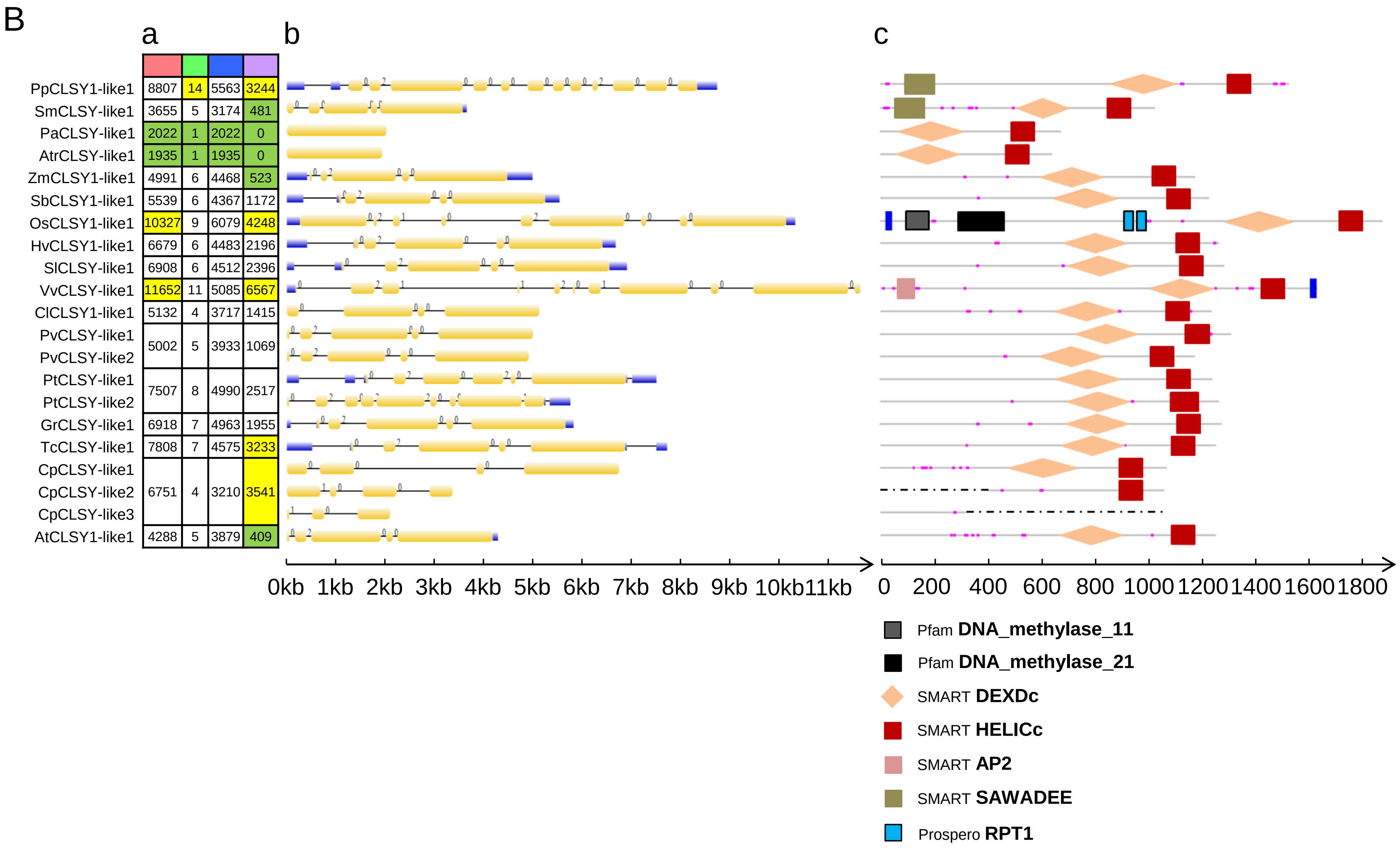
B
a
b
c
PpCLSY1-like1 8807 14 5563 3244
SmCLSY-like1 3655 5 3174 481
PaCLSY-like1 2022 1 2022 0
AtrCLSY-like1 1935 1 1935 0
ZmCLSY1-like1 4991 6 4468 523
SbCLSY1-like1 5539 6 4367 1172
OsCLSY1-like1 10327 9 6079 4248
HvCLSY1-like1 6679 6 4483 2196
SlCLSY-like1 6908 6 4512 2396
VvCLSY1-like1 11652 11 5085 6567
ClCLSY-like1 5132 4 3717 1415
PvCLSY-like1
PvCLSY-like2 5002 5 3933 1069
PtCLSY-like1
PtCLSY-like2 7507 8 4990 2517
GrCLSY-like1 6918 7 4963 1955
TcCLSY-like1 7808 7 4575 3233
CpCLSY-like1
CpCLSY-like2 6751 4 3210 3541
CpCLSY-like3
AtCLSY1-like1 4288 5 3879 409
0kb 1kb 2kb 3kb 4kb 5kb 6kb 7kb 8kb 9kb 10kb 11kb
0 200 400 600 800 1000 1200 1400 1600 1800
Pfam DNA_methylase_11
Pfam DNA_methylase_21
SMART DEXDc
SMART HELICc
SMART AP2
SMART SAWADEE
Prospero RPT1

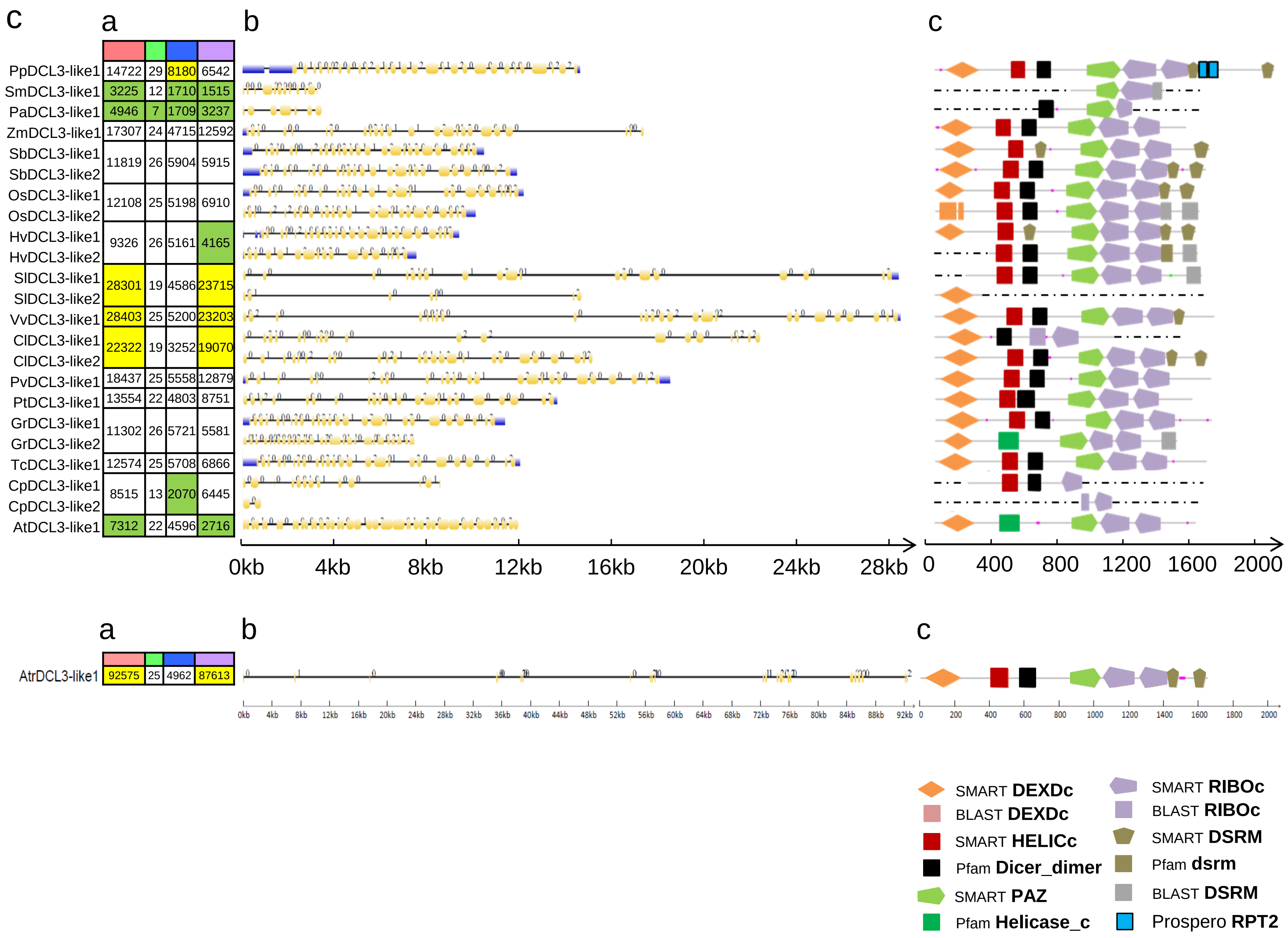

c
a
b
c
PpDCL3-like1 14722 29 8180 6542
SmDCL3-like1 3225 12 1710 1515
PaDCL3-like1 4946 7 1709 3237
ZmDCL3-like1 17307 24 4715 12592
SbDCL3-like1
SbDCL3-like2 11819 26 5904 5915
OsDCL3-like1
OsDCL3-like2 12108 25 5198 6910
HvDCL3-like1
HvDCL3-like2 9326 26 5161 4165
SlDCL3-like1
SlDCL3-like2 28301 19 4586 23715
VvDCL3-like1 28403 25 5200 23203
ClDCL3-like1
ClDCL3-like2 22322 19 3252 19070
PvDCL3-like1 18437 25 5558 12879
PtDCL3-like1 13554 22 4803 8751
GrDCL3-like1
GrDCL3-like2 11302 26 5721 5581
TcDCL3-like1 12574 25 5708 6866
CpDCL3-like1
CpDCL3-like2 8515 13 2070 6445
AtDCL3-like1 7312 22 4596 2716
0kb 4kb 8kb 12kb 16kb 20kb 24kb 28kb
0 400 800 1200 1600 2000
a
b
c
AtrDCL3-like1 92575 25 4962 87613
0kb 4kb 8kb 12kb 16kb 20kb 24kb 28kb 32kb 36kb 40kb 44kb 48kb 52kb 56kb 60kb 64kb 68kb 72kb 76kb 80kb 84kb 88kb 92kb
0 200 400 600 800 1000 1200 1400 1600 1800 2000
SMART DEXDc
BLAST DEXDc
SMART HELICc
Pfam Dicer_dimer
SMART PAZ
Pfam Helicase_c
SMART RIBOc
BLAST RIBOc
SMART DSRM
Pfam dsrm
BLAST DSRM
Prospero RPT2

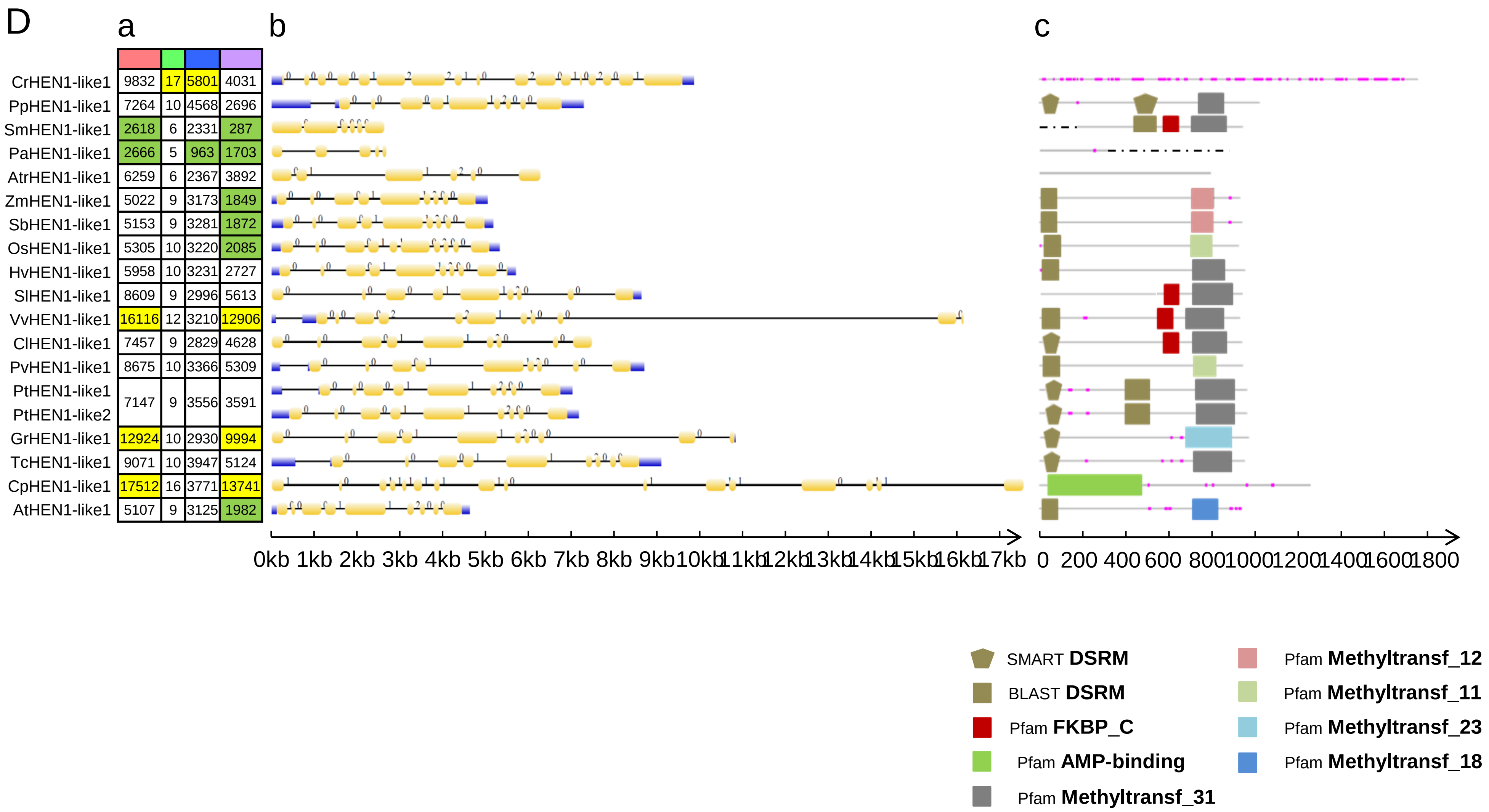
D
a
b
c
CrHEN1-like1 9832 17 5801 4031
PpHEN1-like1 7264 10 4568 2696
SmHEN1-like1 2618 6 2331 287
PaHEN1-like1 2666 5 963 1703
AtrHEN1-like1 6259 6 2367 3892
ZmHEN1-like1 5022 9 3173 1849
SbHEN1-like1 5153 9 3281 1872
OsHEN1-like1 5305 10 3220 2085
HvHEN1-like1 5958 10 3231 2727
SlHEN1-like1 8609 9 2996 5613
VvHEN1-like1 16116 12 3210 12906
ClHEN1-like1 7457 9 2829 4628
PvHEN1-like1 8675 10 3366 5309
PtHEN1-like1 7147 9 3556 3591
PtHEN1-like2
GrHEN1-like1 12924 10 2930 9994
TcHEN1-like1 9071 10 3947 5124
CpHEN1-like1 17512 16 3771 13741
AtHEN1-like1 5107 9 3125 1982
0kb 1kb 2kb 3kb 4kb 5kb 6kb 7kb 8kb 9kb 10kb 11kb 12kb 13kb 14kb 15kb 16kb 17kb
0 200 400 600 800 1000 1200 1400 1600 1800
SMART DSRM
BLAST DSRM
Pfam FKBP_C
Pfam AMP-binding
Pfam Methyltransf_31
Pfam Methyltransf_12
Pfam Methyltransf_11
Pfam Methyltransf_23
Pfam Methyltransf_18

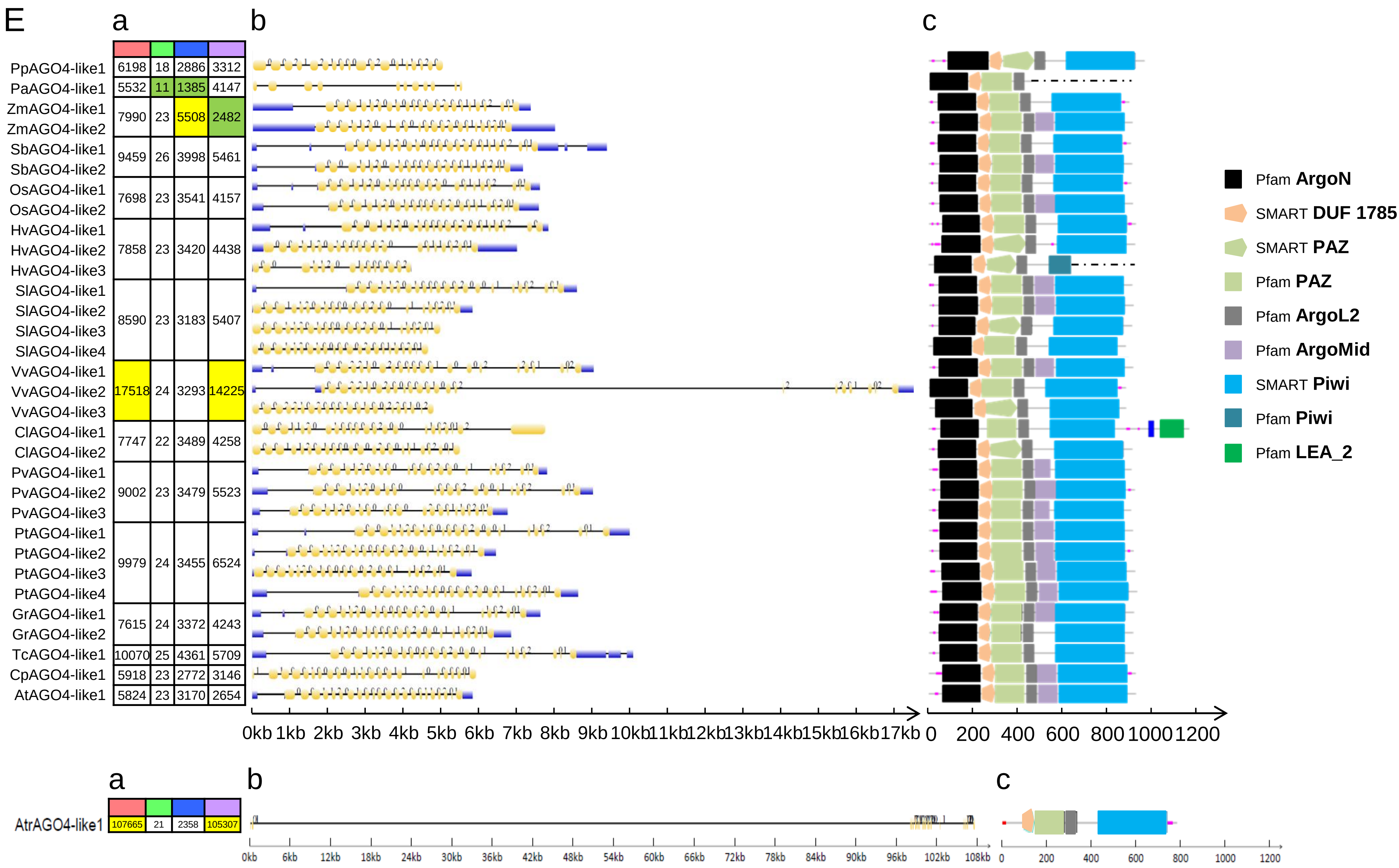
E
a
b
c
PpAGO4-like1 6198 18 2886 3312
PaAGO4-like1 5532 11 1385 4147
ZmAGO4-like1
ZmAGO4-like2
7990 23 5508 2482
SbAGO4-like1
SbAGO4-like2
9459 26 3998 5461
OsAGO4-like1
OsAGO4-like2
7698 23 3541 4157
HvAGO4-like1
HvAGO4-like2
HvAGO4-like3
7858 23 3420 4438
SlAGO4-like1
SlAGO4-like2
SlAGO4-like3
SlAGO4-like4
8590 23 3183 5407
VvAGO4-like1
VvAGO4-like2
VvAGO4-like3
17518 24 3293 14225
ClAGO4-like1
ClAGO4-like2
7747 22 3489 4258
PvAGO4-like1
PvAGO4-like2
PvAGO4-like3
9002 23 3479 5523
PtAGO4-like1
PtAGO4-like2
PtAGO4-like3
PtAGO4-like4
9979 24 3455 6524
GrAGO4-like1
GrAGO4-like2
7615 24 3372 4243
TcAGO4-like1 10070 25 4361 5709
CpAGO4-like1 5918 23 2772 3146
AtAGO4-like1 5824 23 3170 2654
0kb 1kb 2kb 3kb 4kb 5kb 6kb 7kb 8kb 9kb 10kb 11kb 12kb 13kb 14kb 15kb 16kb 17kb
0 200 400 600 800 1000 1200
Pfam ArgoN
SMART DUF 1785
SMART PAZ
Pfam PAZ
Pfam ArgoL2
Pfam ArgoMid
SMART Piwi
Pfam Piwi
Pfam LEA_2
a
b
c
AtrAGO4-like1 107665 21 2358 105307
0kb 6kb 12kb 18kb 24kb 30kb 36kb 42kb 48kb 54kb 60kb 66kb 72kb 78kb 84kb 90kb 96kb 102kb 108kb
0 200 400 600 800 1000 1200

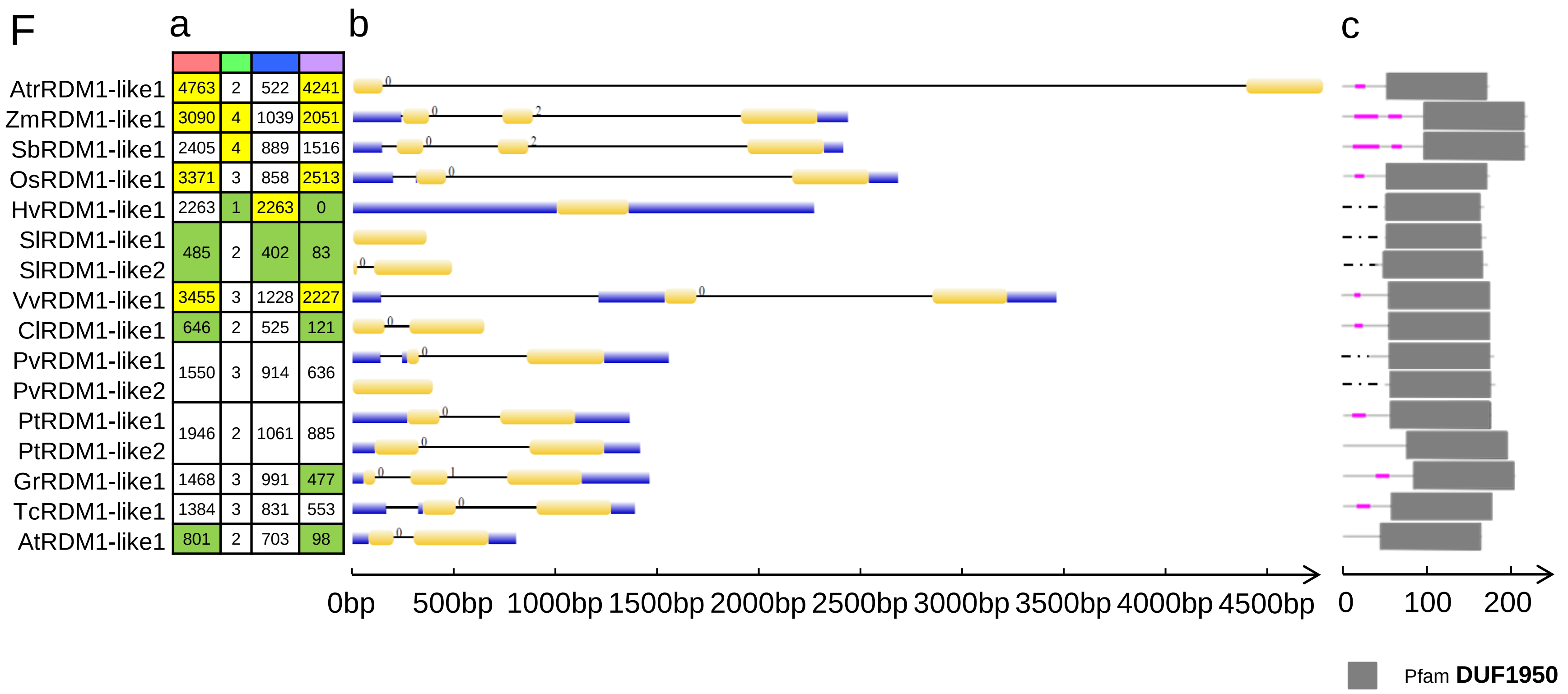
F
a
b
c
AtrRDM1-like1 4763 2 522 4241
ZmRDM1-like1 3090 4 1039 2051
SbRDM1-like1 2405 4 889 1516
OsRDM1-like1 3371 3 858 2513
HvRDM1-like1 2263 1 2263 0
SlRDM1-like1
SlRDM1-like2
485 2 402 83
VvRDM1-like1 3455 3 1228 2227
ClRDM1-like1 646 2 525 121
PvRDM1-like1
PvRDM1-like2
1550 3 914 636
PtRDM1-like1
PtRDM1-like2
1946 2 1061 885
GrRDM1-like1 1468 3 991 477
TcRDM1-like1 1384 3 831 553
AtRDM1-like1 801 2 703 98
0bp 500bp 1000bp 1500bp 2000bp 2500bp 3000bp 3500bp 4000bp 4500bp
0 100 200
Pfam DUF1950

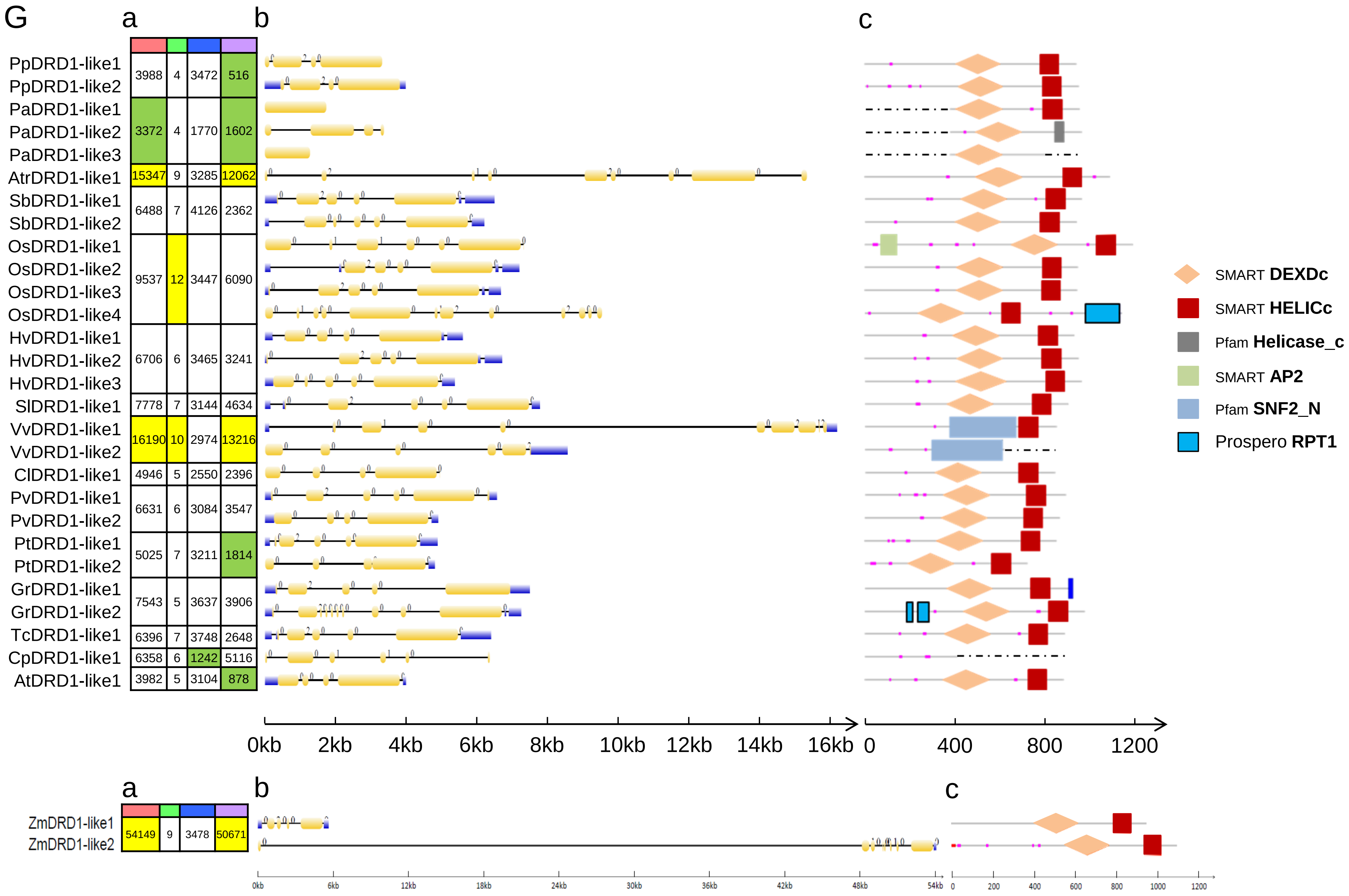
G
a
b
c
PpDRD1-like1
PpDRD1-like2
PaDRD1-like1
PaDRD1-like2
PaDRD1-like3
AtrDRD1-like1
SbDRD1-like1
SbDRD1-like2
OsDRD1-like1
OsDRD1-like2
OsDRD1-like3
OsDRD1-like4
HvDRD1-like1
HvDRD1-like2
HvDRD1-like3
SlDRD1-like1
VvDRD1-like1
VvDRD1-like2
ClDRD1-like1
PvDRD1-like1
PvDRD1-like2
PtDRD1-like1
PtDRD1-like2
GrDRD1-like1
GrDRD1-like2
TcDRD1-like1
CpDRD1-like1
AtDRD1-like1
3988 4 3472 516
3372 4 1770 1602
15347 9 3285 12062
6488 7 4126 2362
9537 12 3447 6090
6706 6 3465 3241
7778 7 3144 4634
16190 10 2974 13216
4946 5 2550 2396
6631 6 3084 3547
5025 7 3211 1814
7543 5 3637 3906
6396 7 3748 2648
6358 6 1242 5116
3982 5 3104 878
SMART DEXDc
SMART HELICc
Pfam Helicase_c
SMART AP2
Pfam SNF2_N
Prospero RPT1
0kb 2kb 4kb 6kb 8kb 10kb 12kb 14kb 16kb
0 400 800 1200
a
b
c
ZmDRD1-like1
ZmDRD1-like2
54149 9 3478 50671
0kb 6kb 12kb 18kb 24kb 30kb 36kb 42kb 48kb 54kb
0 200 400 600 800 1000 1200

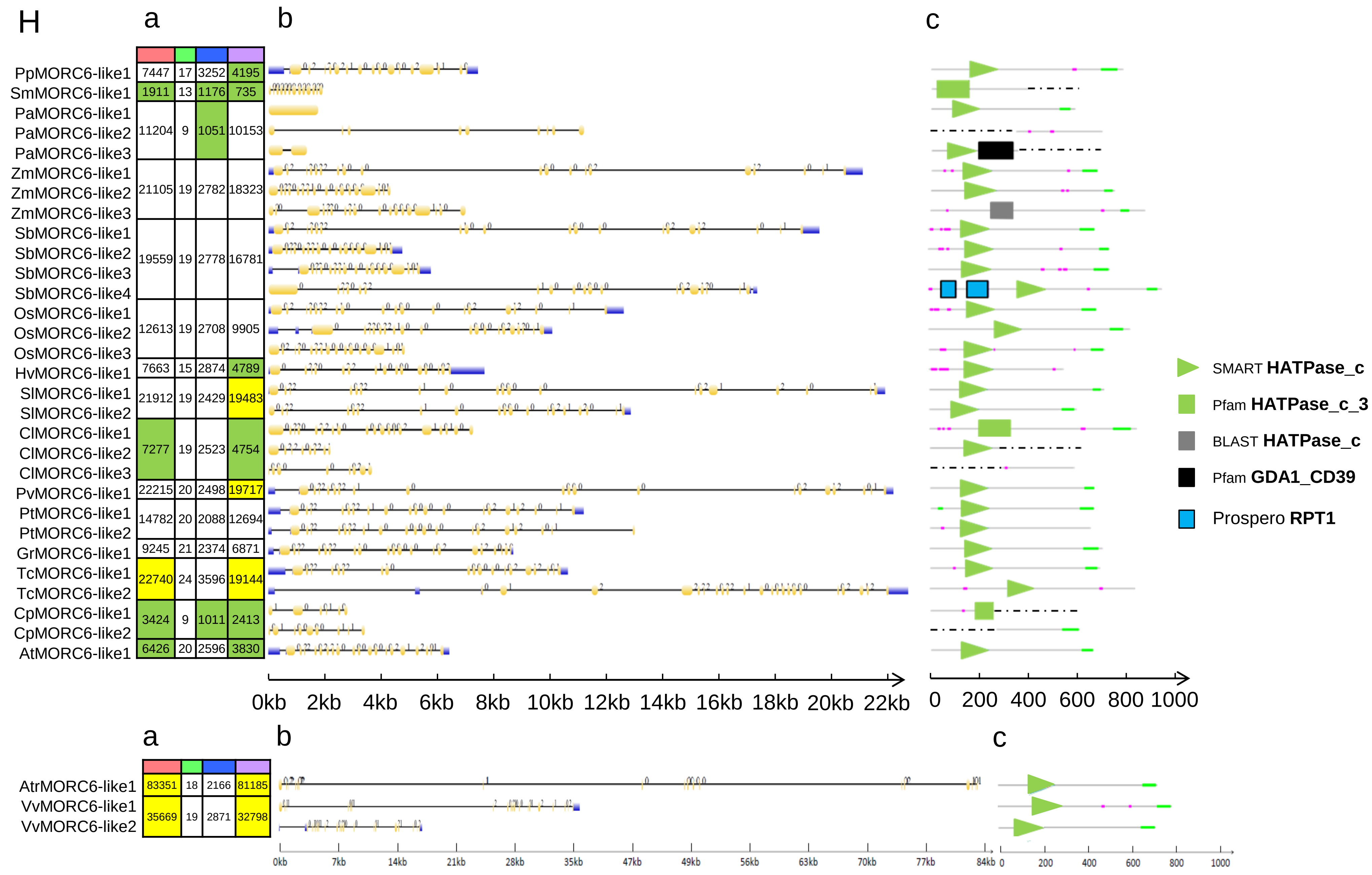

H
a
b
c
PpMORC6-like1
SmMORC6-like1
PaMORC6-like1
PaMORC6-like2
PaMORC6-like3
ZmMORC6-like1
ZmMORC6-like2
ZmMORC6-like3
SbMORC6-like1
SbMORC6-like2
SbMORC6-like3
SbMORC6-like4
OsMORC6-like1
OsMORC6-like2
OsMORC6-like3
HvMORC6-like1
SlMORC6-like1
SlMORC6-like2
ClMORC6-like1
ClMORC6-like2
ClMORC6-like3
PvMORC6-like1
PtMORC6-like1
PtMORC6-like2
GrMORC6-like1
TcMORC6-like1
TcMORC6-like2
CpMORC6-like1
CpMORC6-like2
AtMORC6-like1
7447 17 3252 4195
1911 13 1176 735
11204 9 1051 10153
21105 19 2782 18323
19559 19 2778 16781
12613 19 2708 9905
7663 15 2874 4789
21912 19 2429 19483
7277 19 2523 4754
22215 20 2498 19717
14782 20 2088 12694
9245 21 2374 6871
22740 24 3596 19144
3424 9 1011 2413
6426 20 2596 3830
0kb 2kb 4kb 6kb 8kb 10kb 12kb 14kb 16kb 18kb 20kb 22kb
0 200 400 600 800 1000
SMART HATPase_c
Pfam HATPase_c_3
BLAST HATPase_c
Pfam GDA1_CD39
Prospero RPT1
a
b
c
AtrMORC6-like1
VvMORC6-like1
VvMORC6-like2
83351 18 2166 81185
35669 19 2871 32798
0kb 7kb 14kb 21kb 28kb 35kb 47kb 49kb 56kb 63kb 70kb 77kb 84kb
0 200 400 600 800 1000

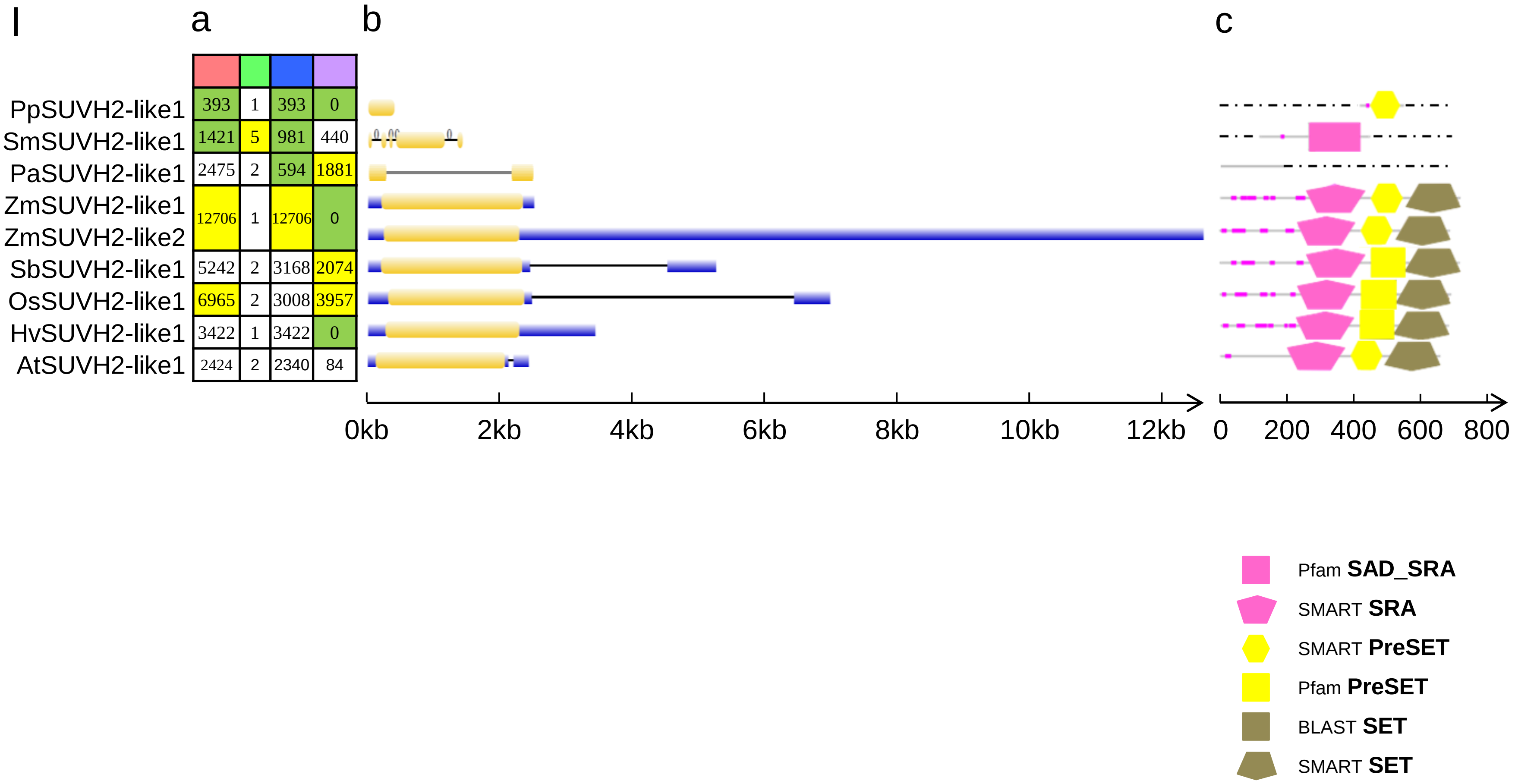
I
a
b
c
PpSUVH2-like1 393 1 393 0
SmSUVH2-like1 1421 5 981 440
PaSUVH2-like1 2475 2 594 1881
ZmSUVH2-like1
ZmSUVH2-like2 12706 1 12706 0
SbSUVH2-like1 5242 2 3168 2074
OsSUVH2-like1 6965 2 3008 3957
HvSUVH2-like1 3422 1 3422 0
AtSUVH2-like1 2424 2 2340 84
0kb
2kb
4kb
6kb
8kb
10kb
12kb
0
200
400
600
800
Pfam SAD_SRA
SMART SRA
SMART PreSET
Pfam PreSET
BLAST SET
SMART SET

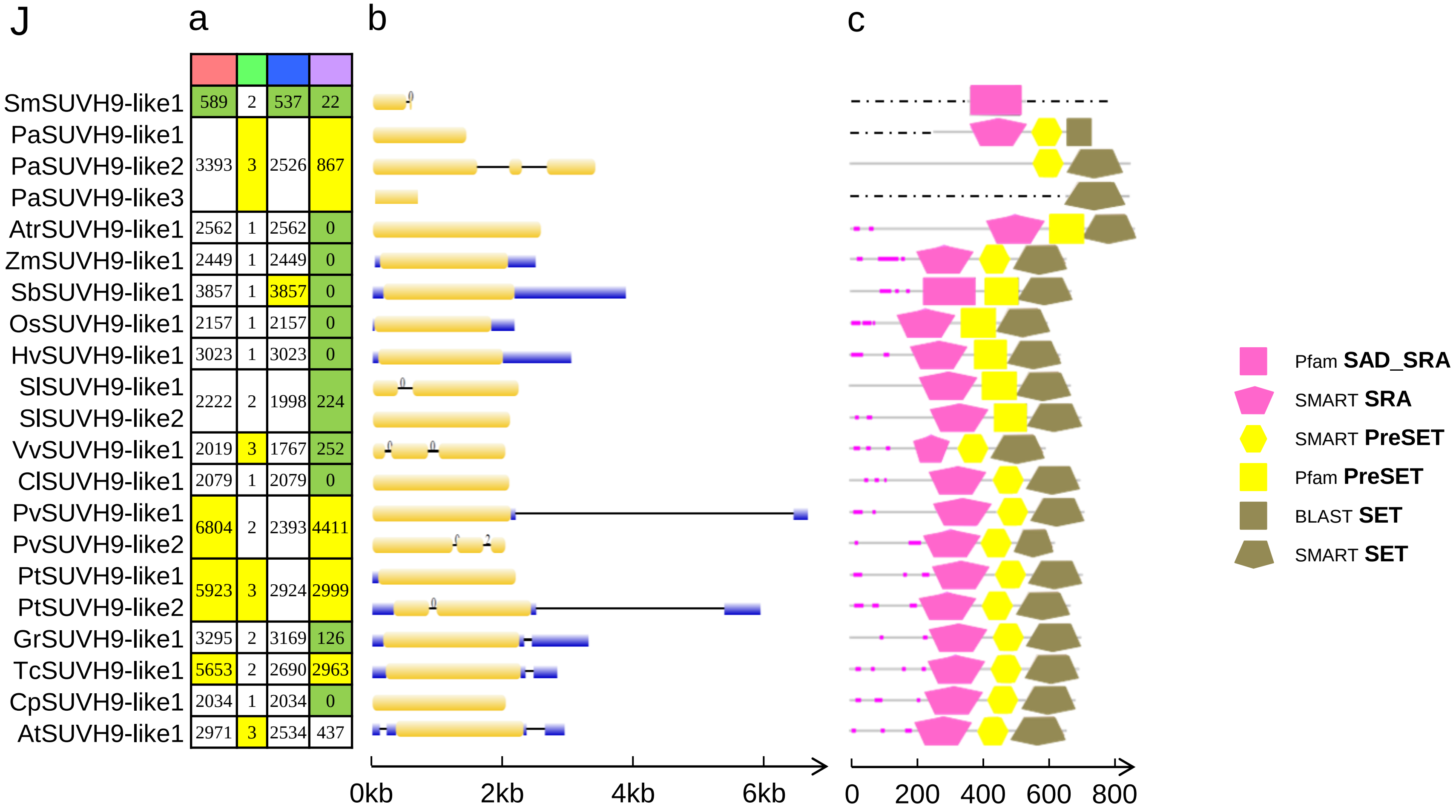

J
a
b
c
SmSUVH9-like1 | 589 | 2 | 537 | 22
PaSUVH9-like1
PaSUVH9-like2 | 3393 | 3 | 2526 | 867
PaSUVH9-like3
AtrSUVH9-like1 | 2562 | 1 | 2562 | 0
ZmSUVH9-like1 | 2449 | 1 | 2449 | 0
SbSUVH9-like1 | 3857 | 1 | 3857 | 0
OsSUVH9-like1 | 2157 | 1 | 2157 | 0
HvSUVH9-like1 | 3023 | 1 | 3023 | 0
SlSUVH9-like1
SlSUVH9-like2 | 2222 | 2 | 1998 | 224
VvSUVH9-like1 | 2019 | 3 | 1767 | 252
ClSUVH9-like1 | 2079 | 1 | 2079 | 0
PvSUVH9-like1
PvSUVH9-like2 | 6804 | 2 | 2393 | 4411
PtSUVH9-like1
PtSUVH9-like2 | 5923 | 3 | 2924 | 2999
GrSUVH9-like1 | 3295 | 2 | 3169 | 126
TcSUVH9-like1 | 5653 | 2 | 2690 | 2963
CpSUVH9-like1 | 2034 | 1 | 2034 | 0
AtSUVH9-like1 | 2971 | 3 | 2534 | 437
0kb
2kb
4kb
6kb
0
200
400
600
800
Pfam SAD_SRA
SMART SRA
SMART PreSET
Pfam PreSET
BLAST SET
SMART SET

K

a

AtrKTF1-like1	13967	18	5115	8852
ZmKTF1-like1<br>ZmKTF1-like2	12611	20	5429	7182
SbKTF1-like1	12916	22	5732	7184
OsKTF1-like1	11931	20	5324	6607
HvKTF1-like1<br>HvKTF1-like2	7198	21	3189	4009
SlKTF1-like1	10468	16	4530	5938
VvKTF1-like1	18109	15	2028	16081
ClKTF1-like1	8948	17	5289	3659
PvKTF1-like1	8353	19	4617	3736
PtKTF1-like1<br>PtKTF1-like2	8981	19	5610	3371
GrKTF1-like1	9659	19	5235	4424
TcKTF1-like1	14343	20	6067	8276
CpKTF1-like1	12355	25	5406	6949
AtKTF1-like1	6585	19	4482	2103

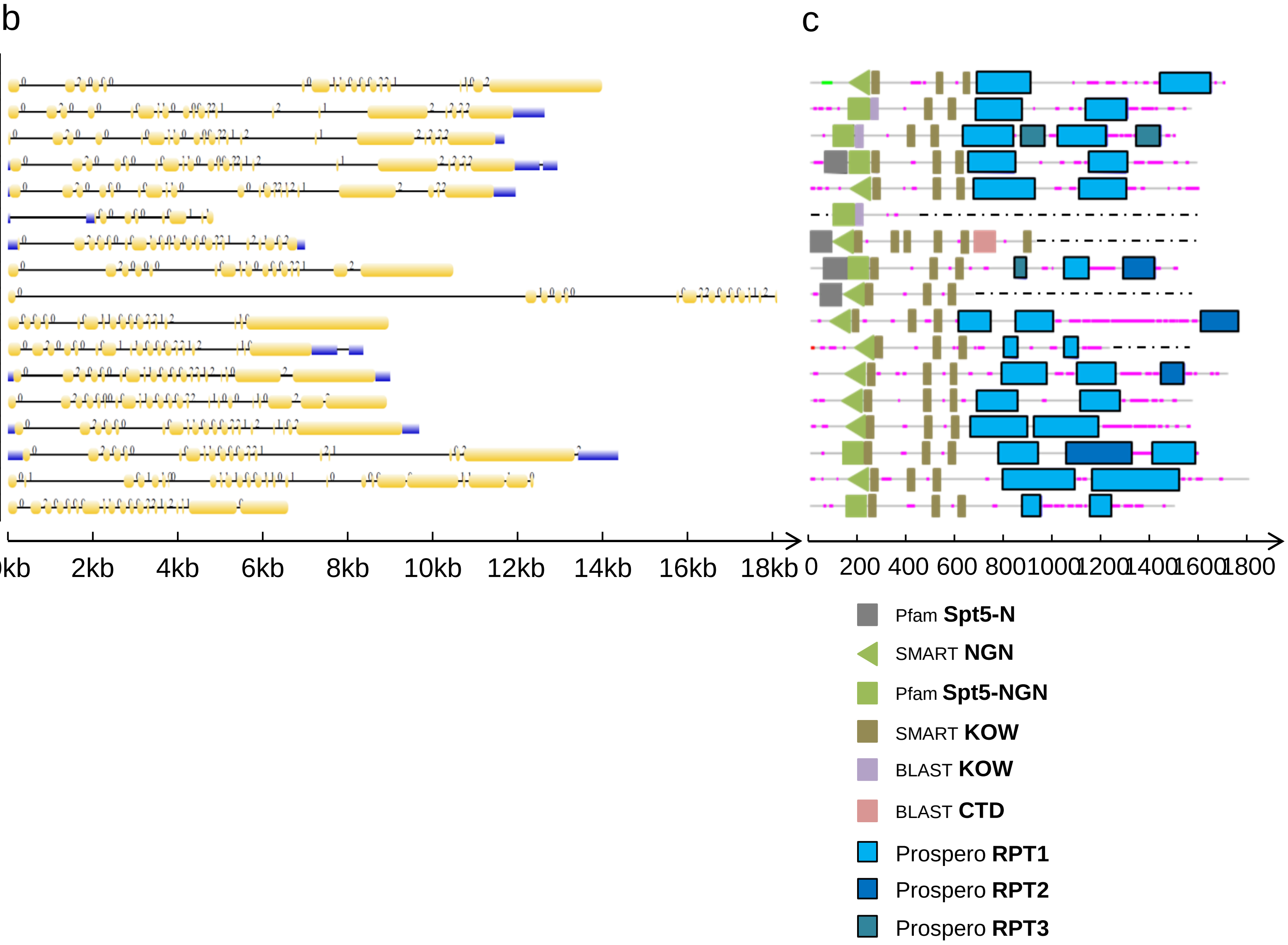

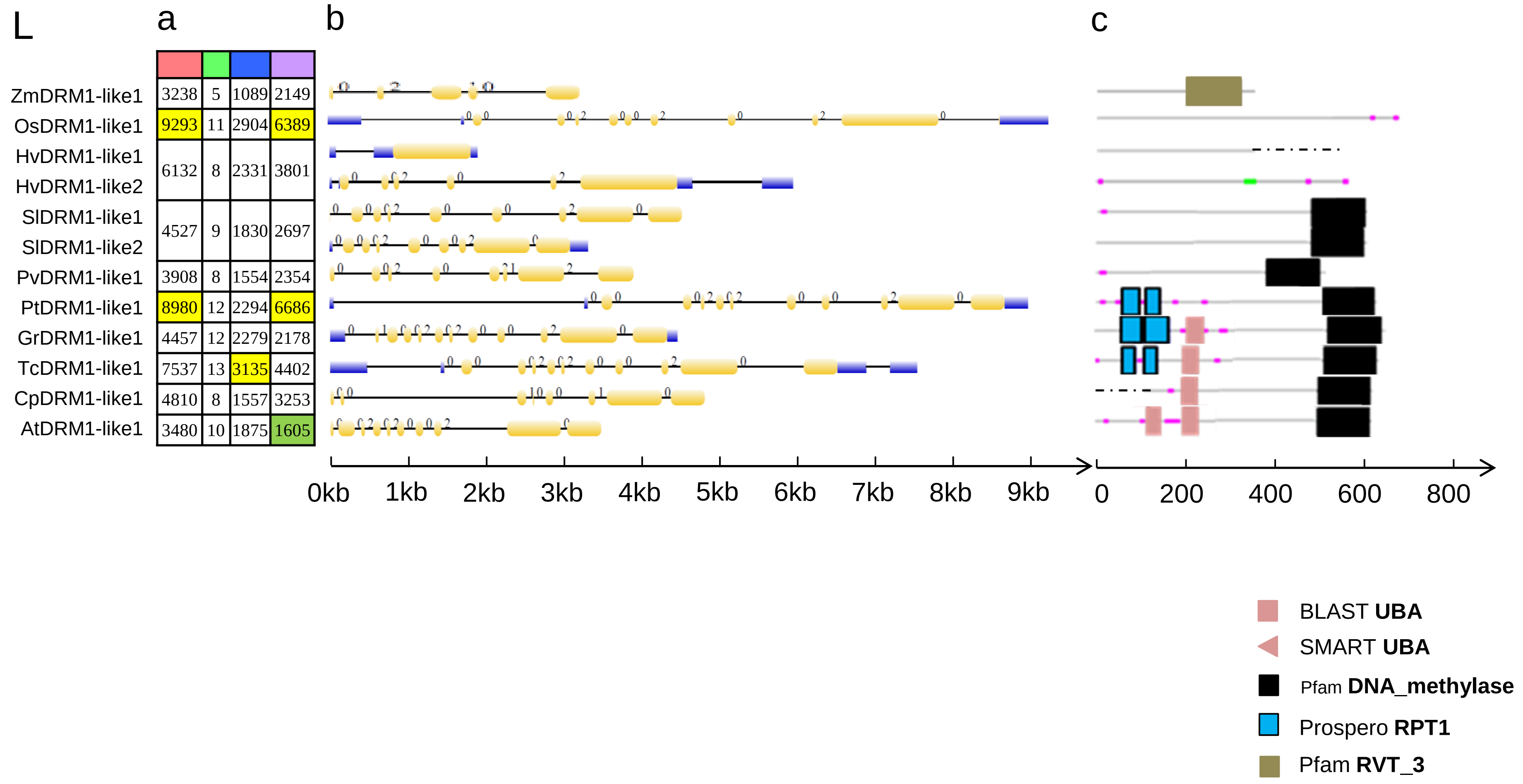
L
a
b
c
ZmDRM1-like1
OsDRM1-like1
HvDRM1-like1
HvDRM1-like2
SlDRM1-like1
SlDRM1-like2
PvDRM1-like1
PtDRM1-like1
GrDRM1-like1
TcDRM1-like1
CpDRM1-like1
AtDRM1-like1
3238 5 1089 2149
9293 11 2904 6389
6132 8 2331 3801
4527 9 1830 2697
3908 8 1554 2354
8980 12 2294 6686
4457 12 2279 2178
7537 13 3135 4402
4810 8 1557 3253
3480 10 1875 1605
0kb 1kb 2kb 3kb 4kb 5kb 6kb 7kb 8kb 9kb
0 200 400 600 800
BLAST UBA
SMART UBA
Pfam DNA_methylase
Prospero RPT1
Pfam RVT_3

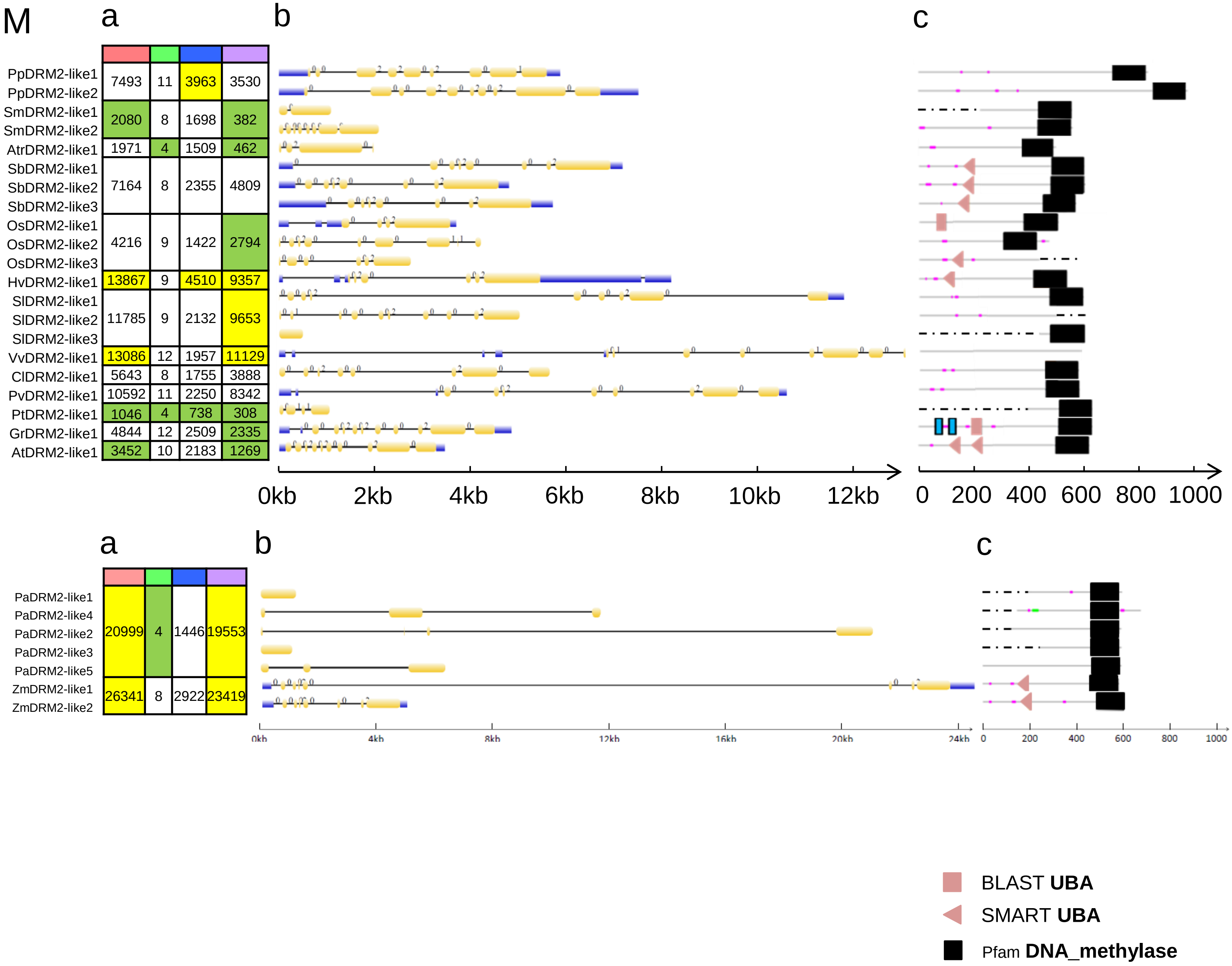

M
a
b
c
PpDRM2-like1
PpDRM2-like2
7493 11 3963 3530
SmDRM2-like1
SmDRM2-like2
2080 8 1698 382
AtrDRM2-like1 1971 4 1509 462
SbDRM2-like1
SbDRM2-like2
SbDRM2-like3
7164 8 2355 4809
OsDRM2-like1
OsDRM2-like2
OsDRM2-like3
4216 9 1422 2794
HvDRM2-like1 13867 9 4510 9357
SlDRM2-like1
SlDRM2-like2
SlDRM2-like3
11785 9 2132 9653
VvDRM2-like1 13086 12 1957 11129
ClDRM2-like1 5643 8 1755 3888
PvDRM2-like1 10592 11 2250 8342
PtDRM2-like1 1046 4 738 308
GrDRM2-like1 4844 12 2509 2335
AtDRM2-like1 3452 10 2183 1269
0kb 2kb 4kb 6kb 8kb 10kb 12kb
0 200 400 600 800 1000
a
b
c
PaDRM2-like1
PaDRM2-like4
PaDRM2-like2
PaDRM2-like3
PaDRM2-like5
20999 4 1446 19553
ZmDRM2-like1
ZmDRM2-like2
26341 8 2922 23419
0kb 4kb 8kb 12kb 16kb 20kb 24kb
0 200 400 600 800 1000
BLAST UBA
SMART UBA
Pfam DNA_methylase

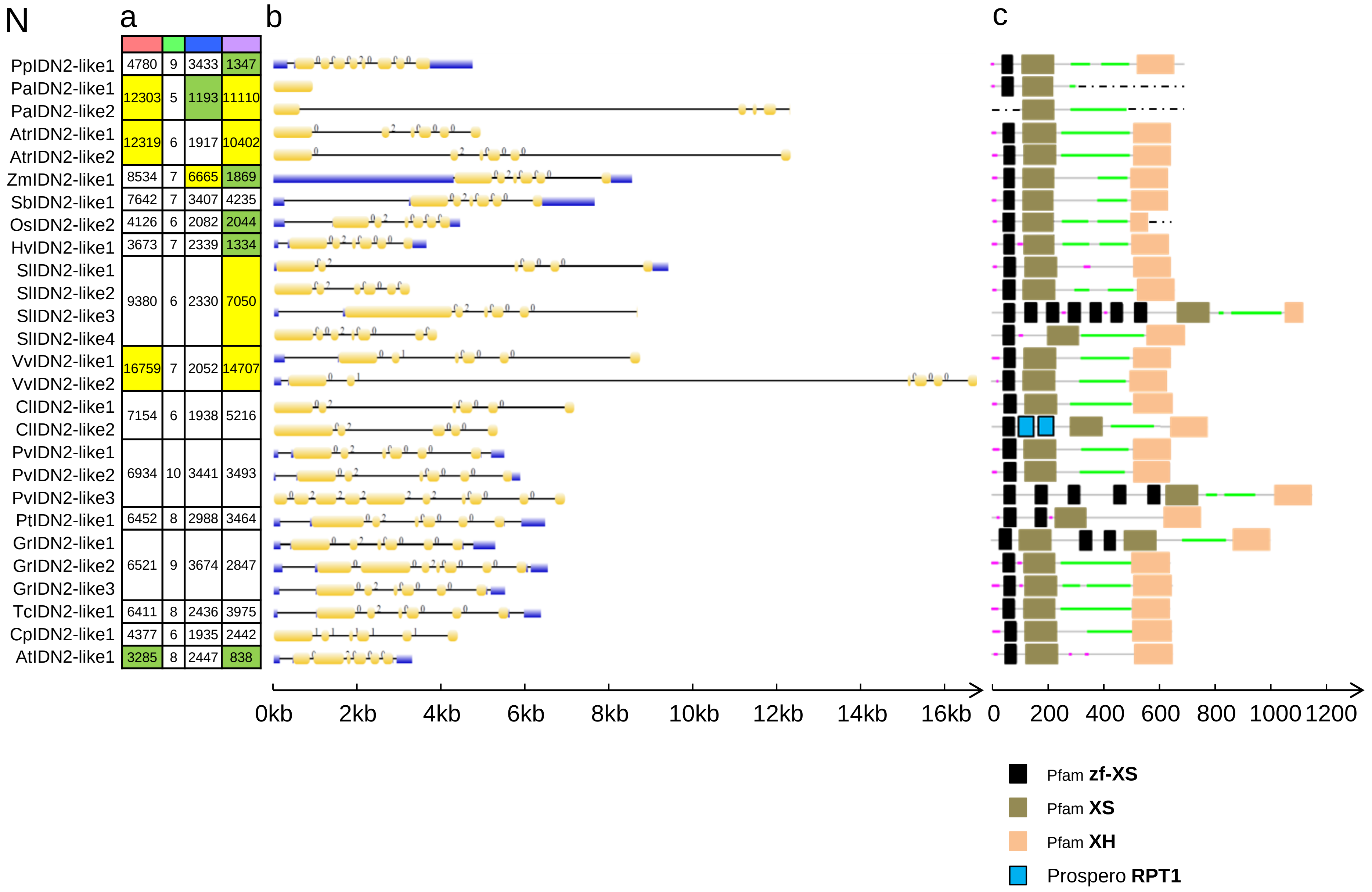

N
a
b
c
PpIDN2-like1 4780 9 3433 1347
PaIDN2-like1
PaIDN2-like2
12303 5 1193 11110
AtrIDN2-like1
AtrIDN2-like2
12319 6 1917 10402
ZmIDN2-like1 8534 7 6665 1869
SbIDN2-like1 7642 7 3407 4235
OsIDN2-like2 4126 6 2082 2044
HvIDN2-like1 3673 7 2339 1334
SlIDN2-like1
SlIDN2-like2
SlIDN2-like3
SlIDN2-like4
9380 6 2330 7050
VvIDN2-like1
VvIDN2-like2
16759 7 2052 14707
ClIDN2-like1
ClIDN2-like2
7154 6 1938 5216
PvIDN2-like1
PvIDN2-like2
PvIDN2-like3
6934 10 3441 3493
PtIDN2-like1 6452 8 2988 3464
GrIDN2-like1
GrIDN2-like2
GrIDN2-like3
6521 9 3674 2847
TcIDN2-like1 6411 8 2436 3975
CpIDN2-like1 4377 6 1935 2442
AtIDN2-like1 3285 8 2447 838
0kb 2kb 4kb 6kb 8kb 10kb 12kb 14kb 16kb
0 200 400 600 800 1000 1200
Pfam zf-XS
Pfam XS
Pfam XH
Prospero RPT1

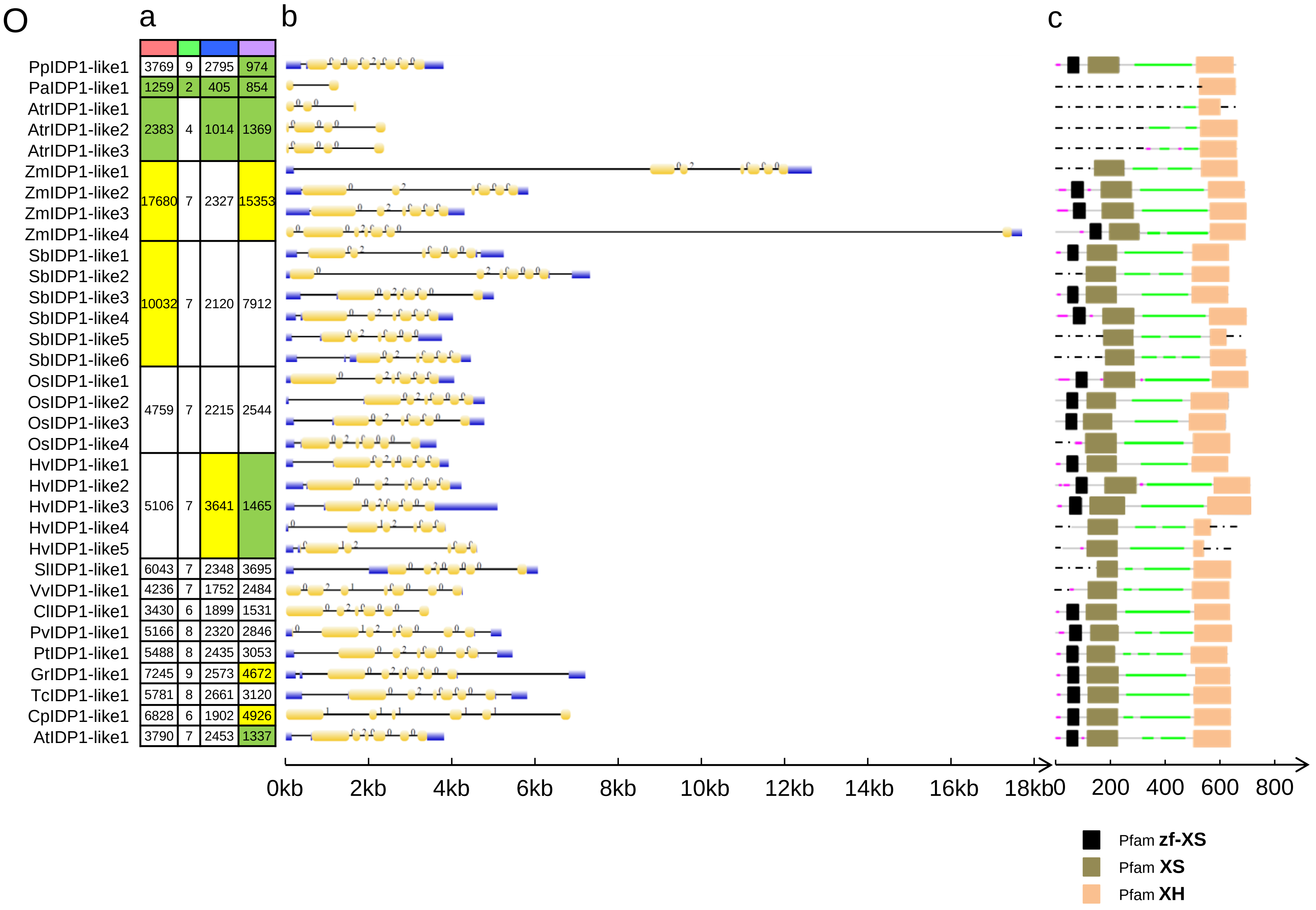
O
a
b
c
PpIDP1-like1 3769 9 2795 974
PaIDP1-like1 1259 2 405 854
AtrIDP1-like1
AtrIDP1-like2 2383 4 1014 1369
AtrIDP1-like3
ZmIDP1-like1
ZmIDP1-like2 17680 7 2327 15353
ZmIDP1-like3
ZmIDP1-like4
SbIDP1-like1
SbIDP1-like2
SbIDP1-like3 10032 7 2120 7912
SbIDP1-like4
SbIDP1-like5
SbIDP1-like6
OsIDP1-like1
OsIDP1-like2 4759 7 2215 2544
OsIDP1-like3
OsIDP1-like4
HvIDP1-like1
HvIDP1-like2
HvIDP1-like3 5106 7 3641 1465
HvIDP1-like4
HvIDP1-like5
SlIDP1-like1 6043 7 2348 3695
VvIDP1-like1 4236 7 1752 2484
ClIDP1-like1 3430 6 1899 1531
PvIDP1-like1 5166 8 2320 2846
PtIDP1-like1 5488 8 2435 3053
GrIDP1-like1 7245 9 2573 4672
TcIDP1-like1 5781 8 2661 3120
CpIDP1-like1 6828 6 1902 4926
AtIDP1-like1 3790 7 2453 1337
0kb 2kb 4kb 6kb 8kb 10kb 12kb 14kb 16kb 18kb
0 200 400 600 800
Pfam zf-XS
Pfam XS
Pfam XH

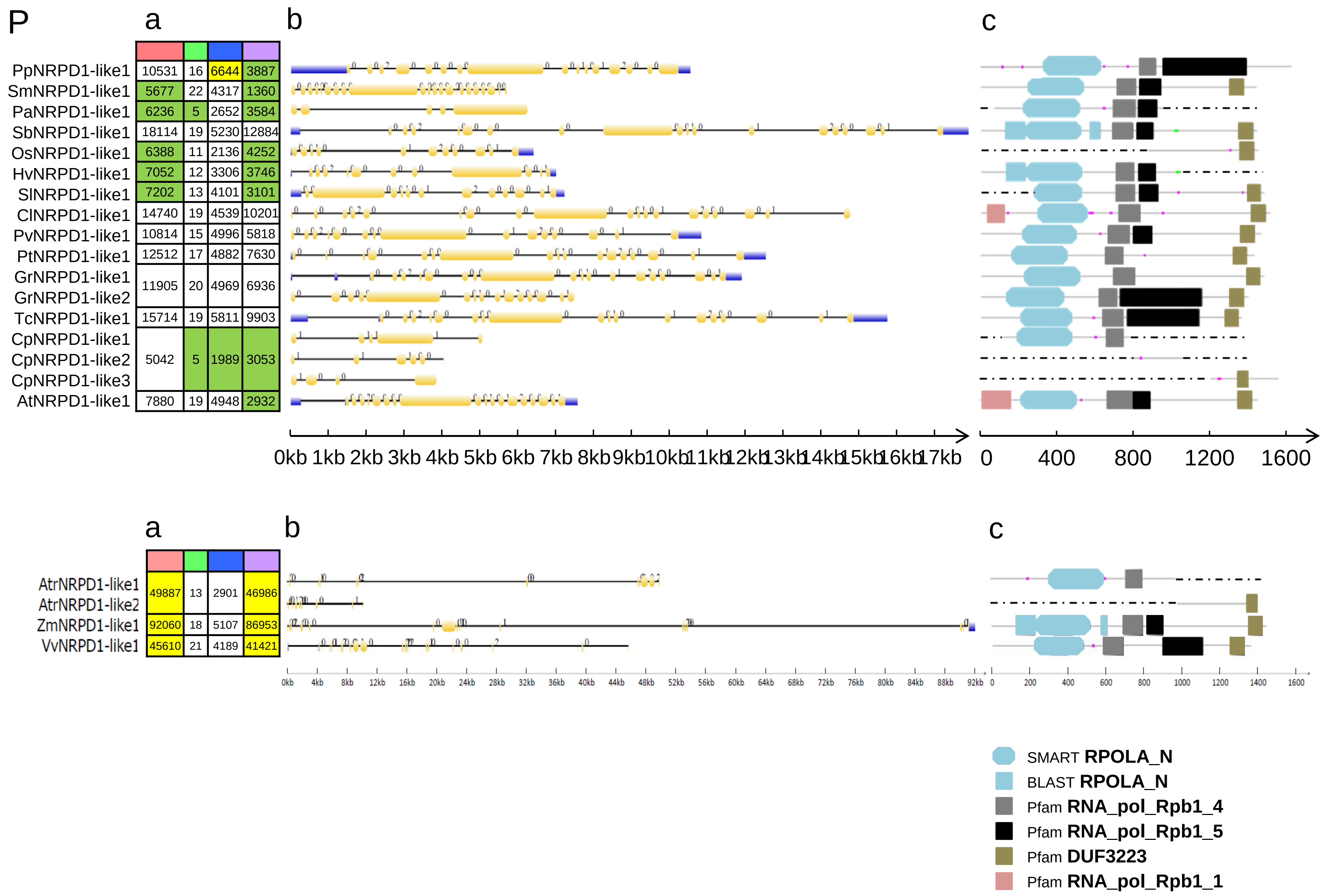

P
a
b
c
PpNRPD1-like1 10531 16 6644 3887
SmNRPD1-like1 5677 22 4317 1360
PaNRPD1-like1 6236 5 2652 3584
SbNRPD1-like1 18114 19 5230 12884
OsNRPD1-like1 6388 11 2136 4252
HvNRPD1-like1 7052 12 3306 3746
SlNRPD1-like1 7202 13 4101 3101
ClNRPD1-like1 14740 19 4539 10201
PvNRPD1-like1 10814 15 4996 5818
PtNRPD1-like1 12512 17 4882 7630
GrNRPD1-like1
GrNRPD1-like2
11905 20 4969 6936
TcNRPD1-like1 15714 19 5811 9903
CpNRPD1-like1
CpNRPD1-like2
CpNRPD1-like3
5042 5 1989 3053
AtNRPD1-like1 7880 19 4948 2932
0kb 1kb 2kb 3kb 4kb 5kb 6kb 7kb 8kb 9kb 10kb 11kb 12kb 13kb 14kb 15kb 16kb 17kb
0 400 800 1200 1600
a
b
c
AtrNRPD1-like1
AtrNRPD1-like2
49887 13 2901 46986
ZmNRPD1-like1 92060 18 5107 86953
VvNRPD1-like1 45610 21 4189 41421
0kb 4kb 8kb 12kb 16kb 20kb 24kb 28kb 32kb 36kb 40kb 44kb 48kb 52kb 56kb 60kb 64kb 68kb 72kb 76kb 80kb 84kb 88kb 92kb
0 200 400 600 800 1000 1200 1400 1600
SMART RPOLA_N
BLAST RPOLA_N
Pfam RNA_pol_Rpb1_4
Pfam RNA_pol_Rpb1_5
Pfam DUF3223
Pfam RNA_pol_Rpb1_1

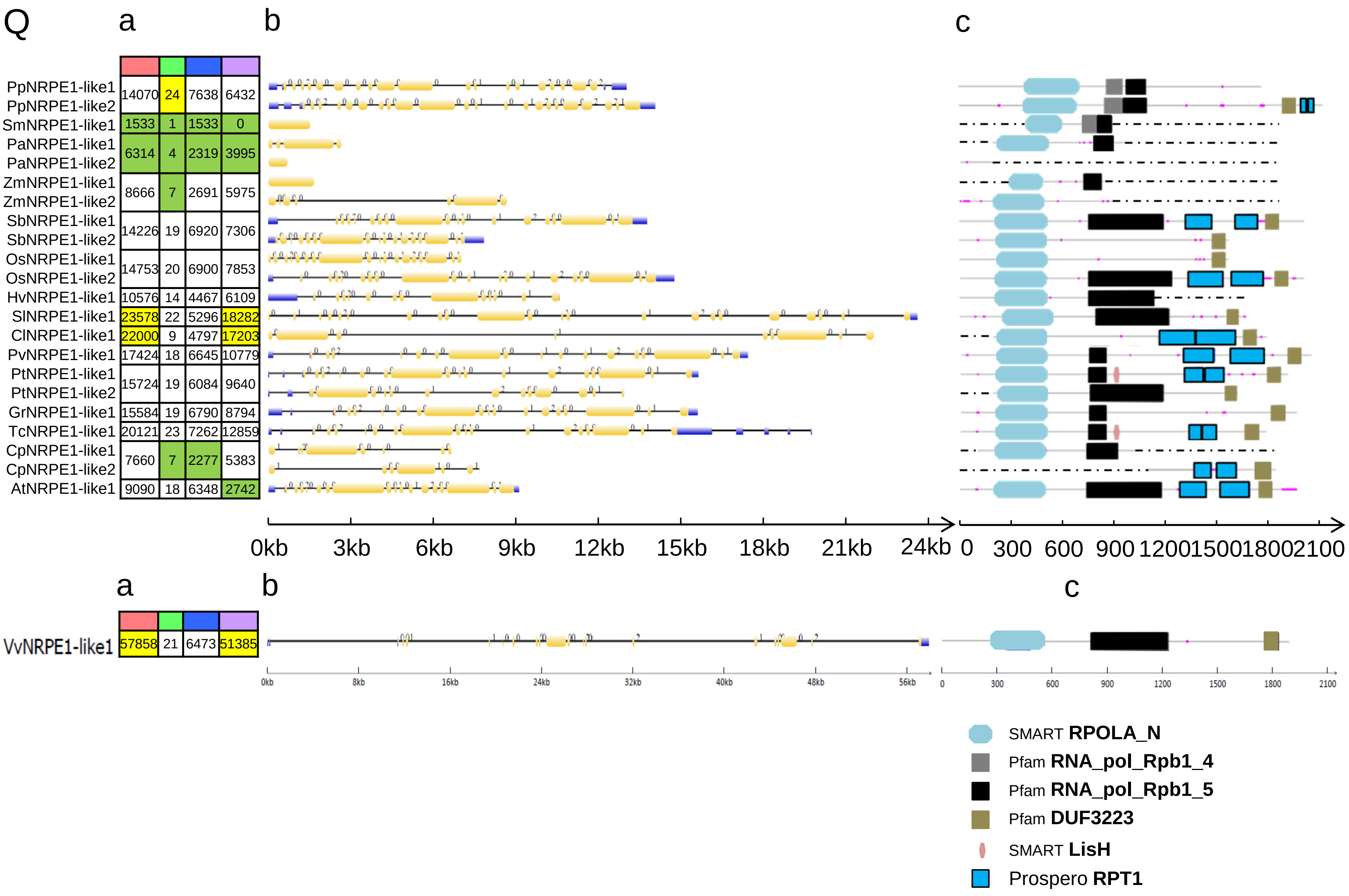

Q
a
b
c
PpNRPE1-like1
PpNRPE1-like2
14070 24 7638 6432
SmNRPE1-like1 1533 1 1533 0
PaNRPE1-like1
PaNRPE1-like2
6314 4 2319 3995
ZmNRPE1-like1
ZmNRPE1-like2
8666 7 2691 5975
SbNRPE1-like1
SbNRPE1-like2
14226 19 6920 7306
OsNRPE1-like1
OsNRPE1-like2
14753 20 6900 7853
HvNRPE1-like1 10576 14 4467 6109
SlNRPE1-like1 23578 22 5296 18282
ClNRPE1-like1 22000 9 4797 17203
PvNRPE1-like1 17424 18 6645 10779
PtNRPE1-like1
PtNRPE1-like2
15724 19 6084 9640
GrNRPE1-like1 15584 19 6790 8794
TcNRPE1-like1 20121 23 7262 12859
CpNRPE1-like1
CpNRPE1-like2
7660 7 2277 5383
AtNRPE1-like1 9090 18 6348 2742
0kb 3kb 6kb 9kb 12kb 15kb 18kb 21kb 24kb
0 300 600 900 1200 1500 1800 2100
a
b
c
VvNRPE1-like1 57858 21 6473 51385
0kb 8kb 16kb 24kb 32kb 40kb 48kb 56kb
0 300 600 900 1200 1500 1800 2100
SMART RPOLA_N
Pfam RNA_pol_Rpb1_4
Pfam RNA_pol_Rpb1_5
Pfam DUF3223
SMART LisH
Prospero RPT1

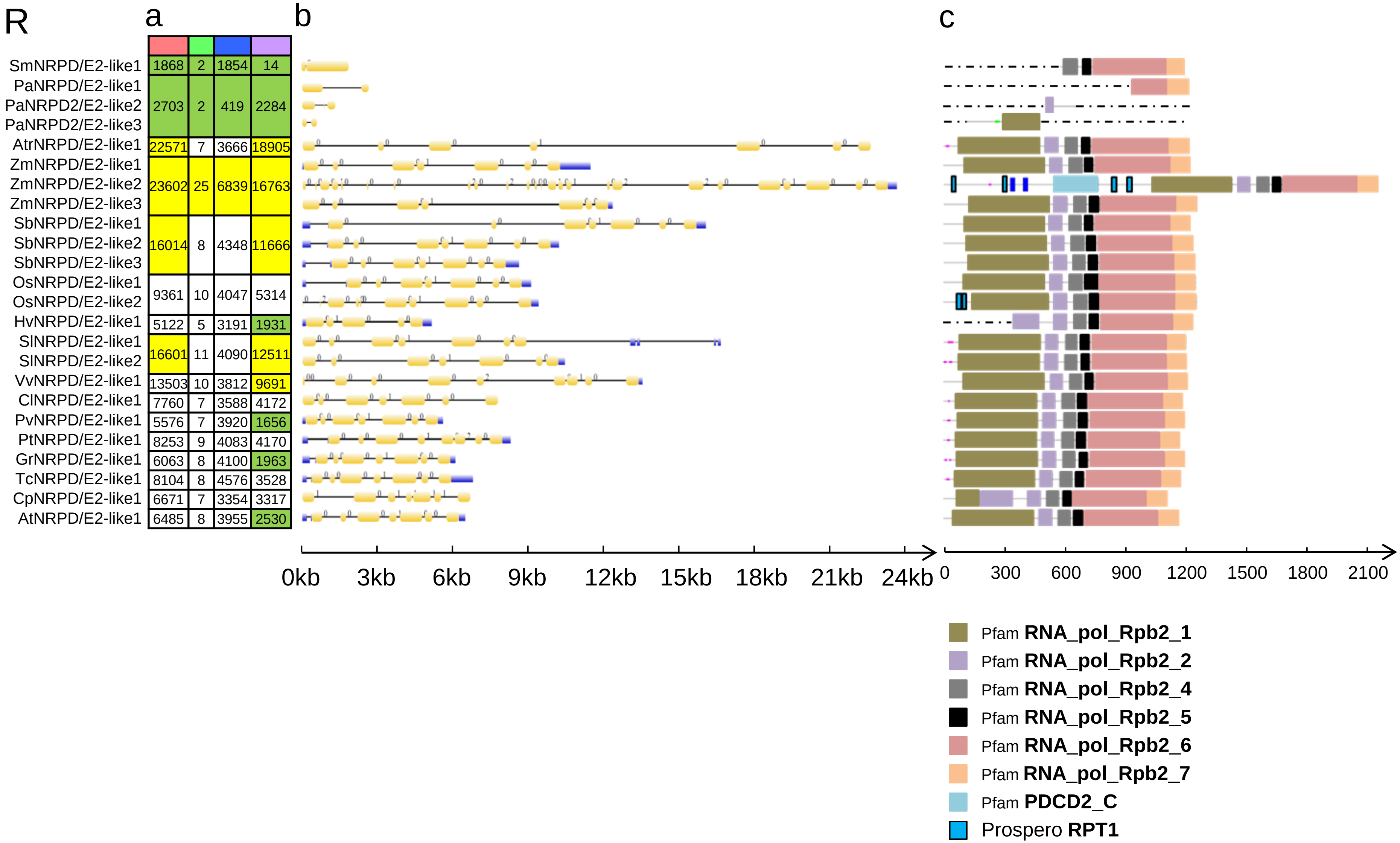

R
a
b
c
SmNRPD/E2-like1 1868 2 1854 14
PaNRPD/E2-like1
PaNRPD2/E2-like2 2703 2 419 2284
PaNRPD2/E2-like3
AtrNRPD/E2-like1 22571 7 3666 18905
ZmNRPD/E2-like1
ZmNRPD/E2-like2 23602 25 6839 16763
ZmNRPD/E2-like3
SbNRPD/E2-like1
SbNRPD/E2-like2 16014 8 4348 11666
SbNRPD/E2-like3
OsNRPD/E2-like1
OsNRPD/E2-like2 9361 10 4047 5314
HvNRPD/E2-like1 5122 5 3191 1931
SlNRPD/E2-like1
SlNRPD/E2-like2 16601 11 4090 12511
VvNRPD/E2-like1 13503 10 3812 9691
ClNRPD/E2-like1 7760 7 3588 4172
PvNRPD/E2-like1 5576 7 3920 1656
PtNRPD/E2-like1 8253 9 4083 4170
GrNRPD/E2-like1 6063 8 4100 1963
TcNRPD/E2-like1 8104 8 4576 3528
CpNRPD/E2-like1 6671 7 3354 3317
AtNRPD/E2-like1 6485 8 3955 2530
0kb 3kb 6kb 9kb 12kb 15kb 18kb 21kb 24kb
0 300 600 900 1200 1500 1800 2100
Pfam RNA_pol_Rpb2_1
Pfam RNA_pol_Rpb2_2
Pfam RNA_pol_Rpb2_4
Pfam RNA_pol_Rpb2_5
Pfam RNA_pol_Rpb2_6
Pfam RNA_pol_Rpb2_7
Pfam PDCD2_C
Prospero RPT1

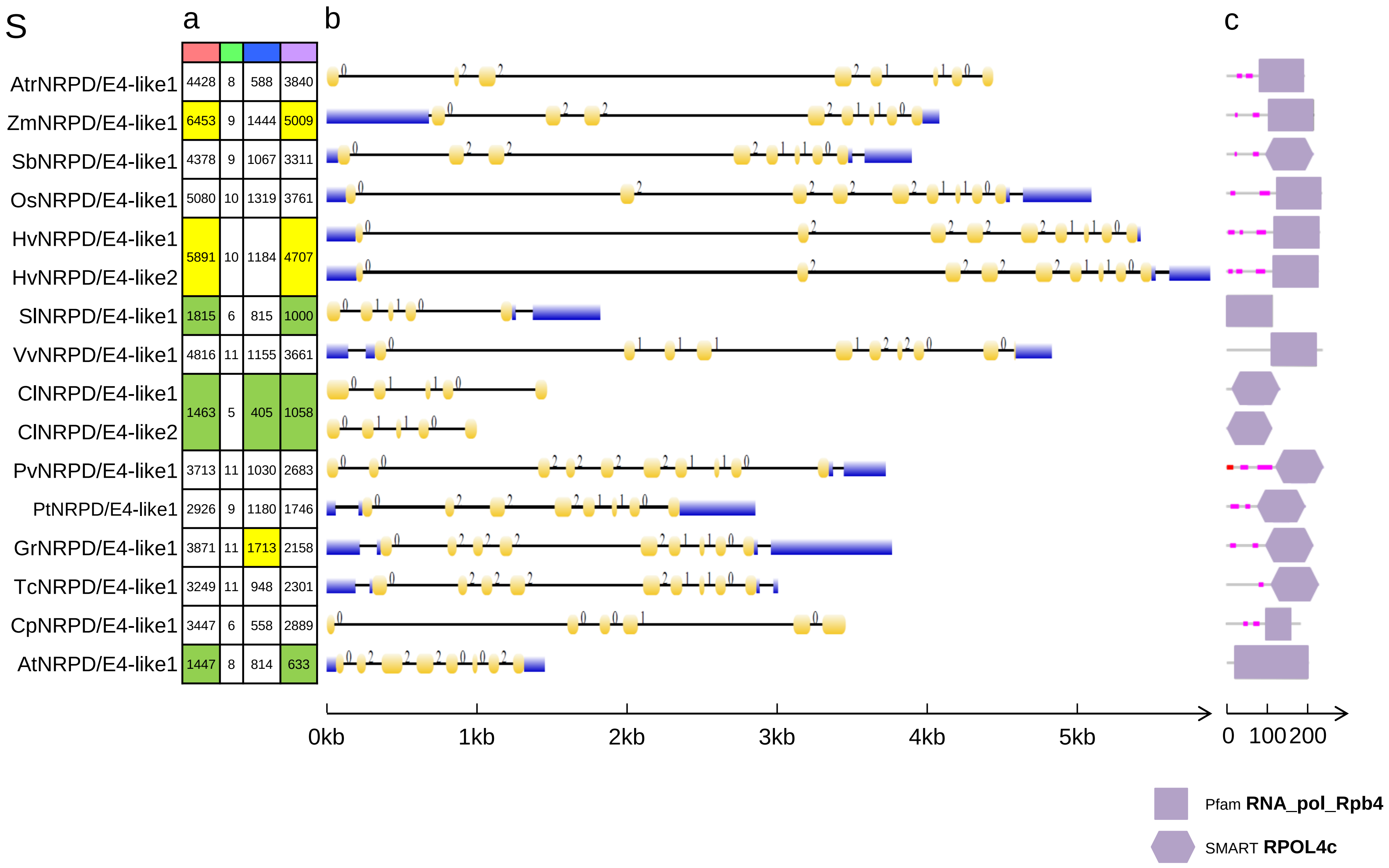
S
a
b
c
AtrNRPD/E4-like1 4428 8 588 3840
ZmNRPD/E4-like1 6453 9 1444 5009
SbNRPD/E4-like1 4378 9 1067 3311
OsNRPD/E4-like1 5080 10 1319 3761
HvNRPD/E4-like1
HvNRPD/E4-like2
5891 10 1184 4707
SlNRPD/E4-like1 1815 6 815 1000
VvNRPD/E4-like1 4816 11 1155 3661
ClNRPD/E4-like1
ClNRPD/E4-like2
1463 5 405 1058
PvNRPD/E4-like1 3713 11 1030 2683
PtNRPD/E4-like1 2926 9 1180 1746
GrNRPD/E4-like1 3871 11 1713 2158
TcNRPD/E4-like1 3249 11 948 2301
CpNRPD/E4-like1 3447 6 558 2889
AtNRPD/E4-like1 1447 8 814 633
0kb
1kb
2kb
3kb
4kb
5kb
0
100
200
Pfam RNA_pol_Rpb4
SMART RPOL4c

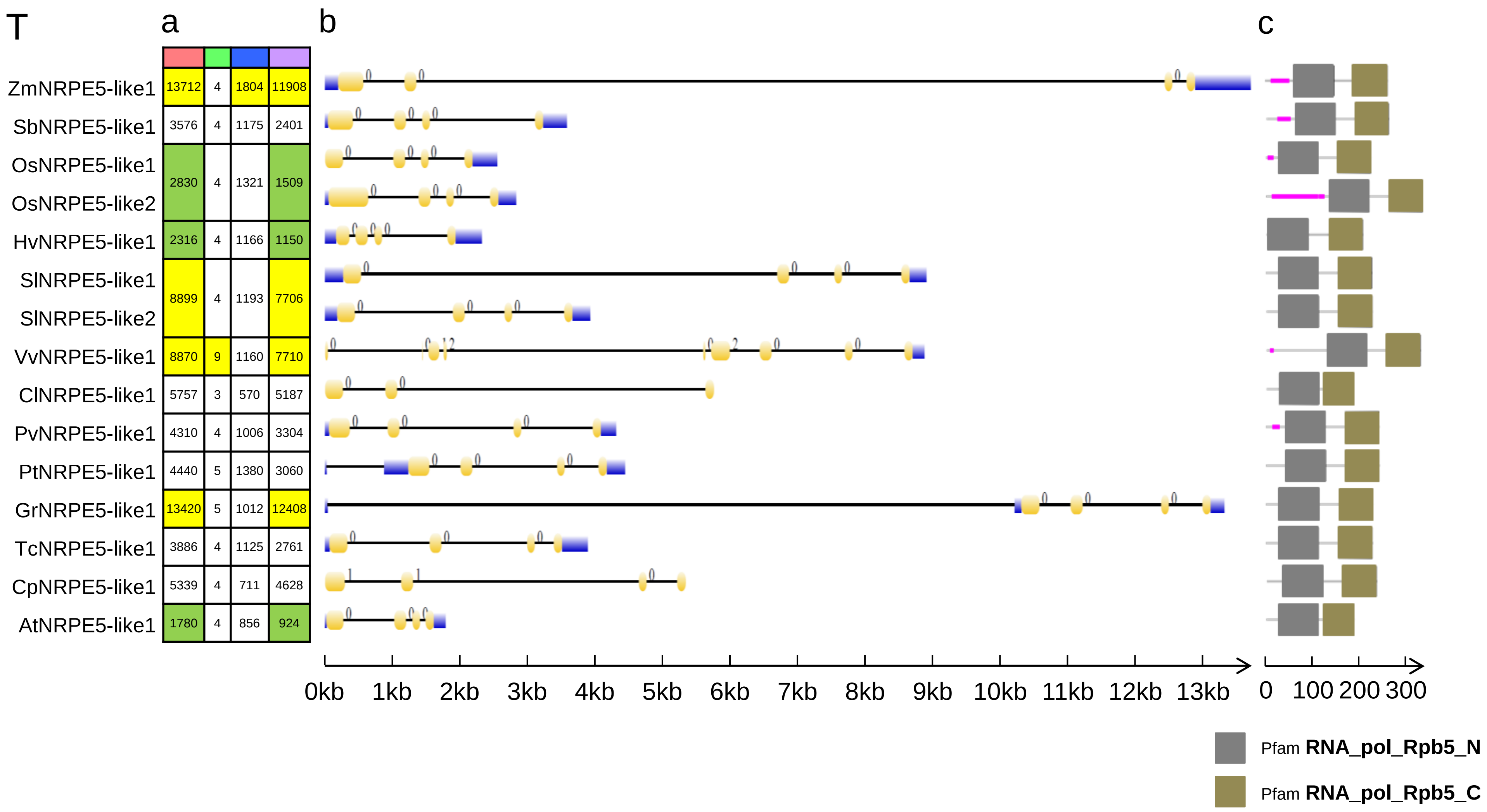

T
a
b
c
ZmNRPE5-like1
SbNRPE5-like1
OsNRPE5-like1
OsNRPE5-like2
HvNRPE5-like1
SlNRPE5-like1
SlNRPE5-like2
VvNRPE5-like1
ClNRPE5-like1
PvNRPE5-like1
PtNRPE5-like1
GrNRPE5-like1
TcNRPE5-like1
CpNRPE5-like1
AtNRPE5-like1
13712 4 1804 11908
3576 4 1175 2401
2830 4 1321 1509
2316 4 1166 1150
8899 4 1193 7706
8870 9 1160 7710
5757 3 570 5187
4310 4 1006 3304
4440 5 1380 3060
13420 5 1012 12408
3886 4 1125 2761
5339 4 711 4628
1780 4 856 924
0kb 1kb 2kb 3kb 4kb 5kb 6kb 7kb 8kb 9kb 10kb 11kb 12kb 13kb
0 100 200 300
Pfam RNA_pol_Rpb5_N
Pfam RNA_pol_Rpb5_C

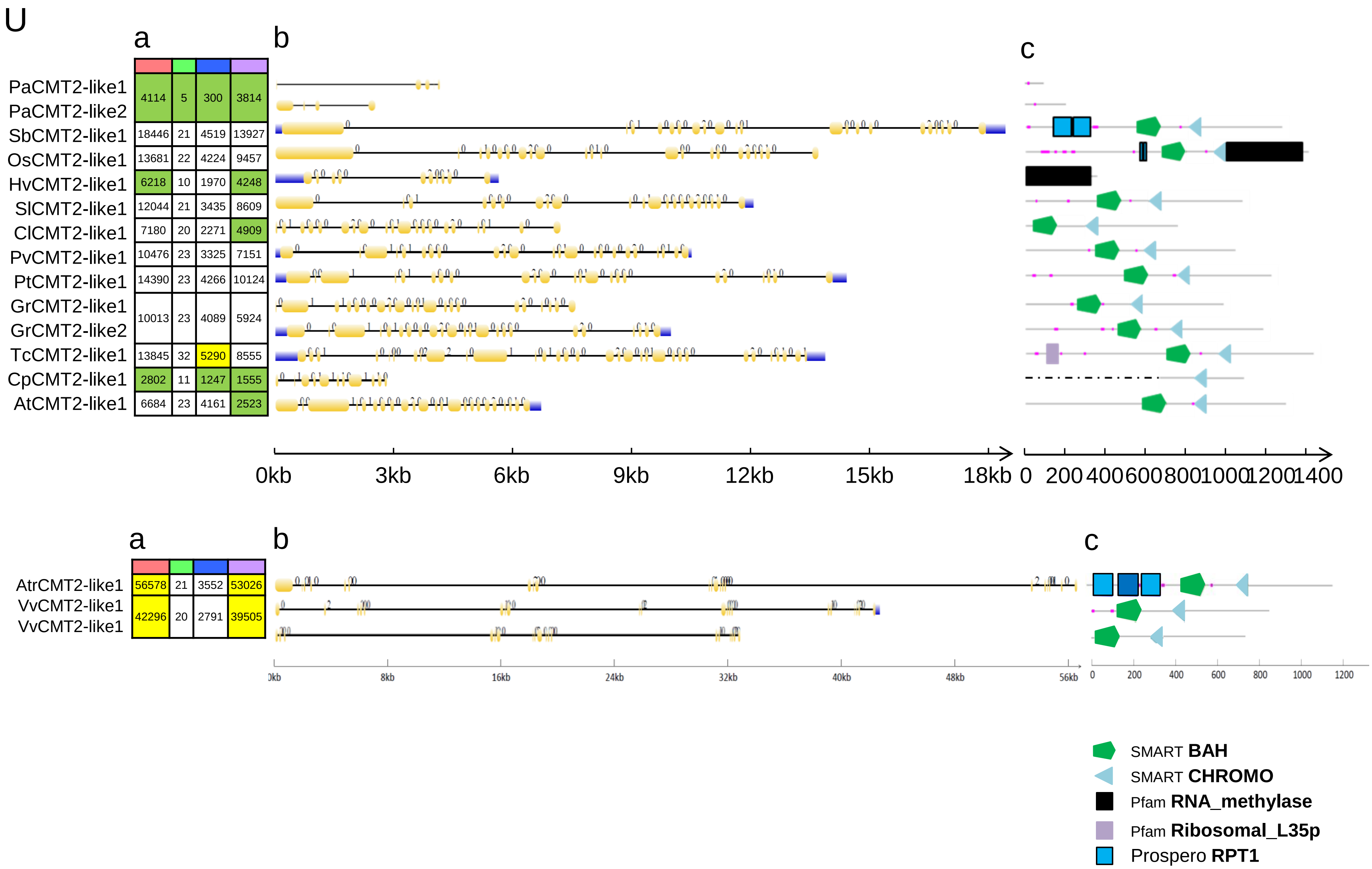
U
a
b
c
PaCMT2-like1
PaCMT2-like2
SbCMT2-like1
OsCMT2-like1
HvCMT2-like1
SlCMT2-like1
ClCMT2-like1
PvCMT2-like1
PtCMT2-like1
GrCMT2-like1
GrCMT2-like2
TcCMT2-like1
CpCMT2-like1
AtCMT2-like1
4114 5 300 3814
18446 21 4519 13927
13681 22 4224 9457
6218 10 1970 4248
12044 21 3435 8609
7180 20 2271 4909
10476 23 3325 7151
14390 23 4266 10124
10013 23 4089 5924
13845 32 5290 8555
2802 11 1247 1555
6684 23 4161 2523
0kb 3kb 6kb 9kb 12kb 15kb 18kb
0 200 400 600 800 1000 1200 1400
a
b
c
AtrCMT2-like1
VvCMT2-like1
VvCMT2-like1
56578 21 3552 53026
42296 20 2791 39505
0kb 8kb 16kb 24kb 32kb 40kb 48kb 56kb
0 200 400 600 800 1000 1200
SMART BAH
SMART CHROMO
Pfam RNA_methylase
Pfam Ribosomal_L35p
Prospero RPT1

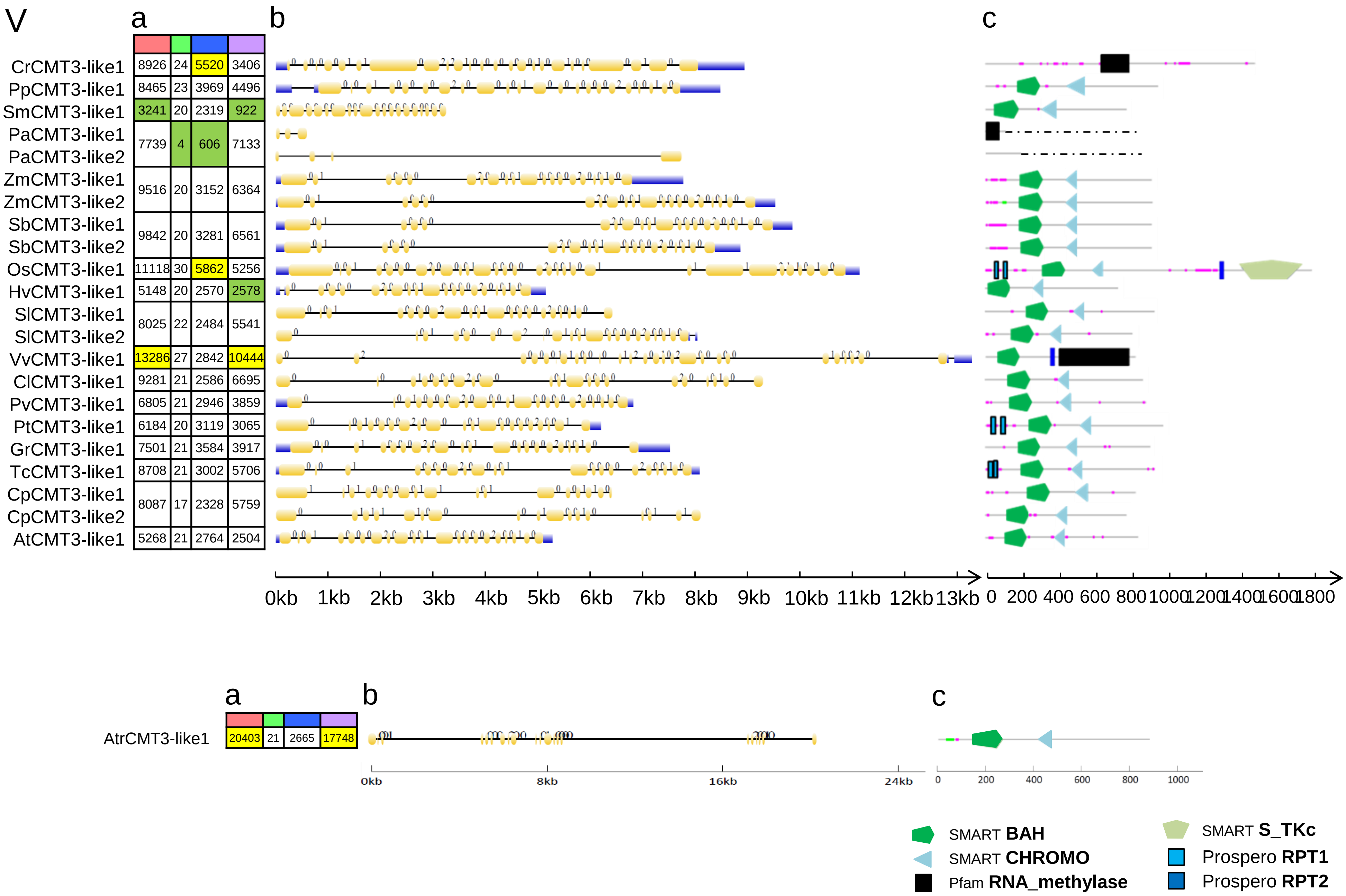

V
a
b
c
CrCMT3-like1 8926 24 5520 3406
PpCMT3-like1 8465 23 3969 4496
SmCMT3-like1 3241 20 2319 922
PaCMT3-like1
PaCMT3-like2 7739 4 606 7133
ZmCMT3-like1
ZmCMT3-like2 9516 20 3152 6364
SbCMT3-like1
SbCMT3-like2 9842 20 3281 6561
OsCMT3-like1 11118 30 5862 5256
HvCMT3-like1 5148 20 2570 2578
SlCMT3-like1
SlCMT3-like2 8025 22 2484 5541
VvCMT3-like1 13286 27 2842 10444
ClCMT3-like1 9281 21 2586 6695
PvCMT3-like1 6805 21 2946 3859
PtCMT3-like1 6184 20 3119 3065
GrCMT3-like1 7501 21 3584 3917
TcCMT3-like1 8708 21 3002 5706
CpCMT3-like1
CpCMT3-like2 8087 17 2328 5759
AtCMT3-like1 5268 21 2764 2504
0kb 1kb 2kb 3kb 4kb 5kb 6kb 7kb 8kb 9kb 10kb 11kb 12kb 13kb
0 200 400 600 800 1000 1200 1400 1600 1800
AtrCMT3-like1 20403 21 2665 17748
0kb 8kb 16kb 24kb
0 200 400 600 800 1000
SMART BAH
SMART CHROMO
Pfam RNA_methylase
SMART S_TKc
Prospero RPT1
Prospero RPT2

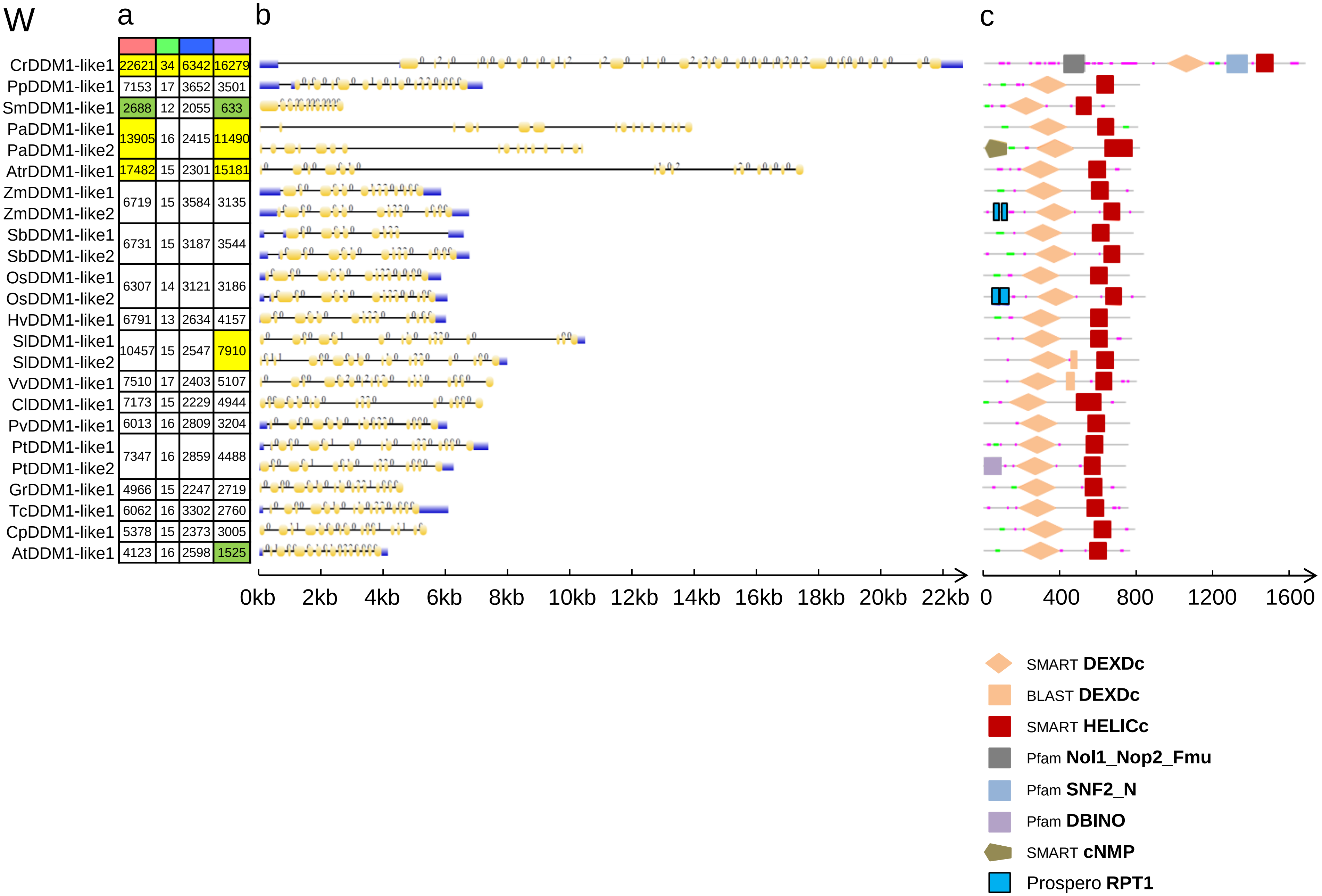
W
a
b
c
CrDDM1-like1 22621 34 6342 16279
PpDDM1-like1 7153 17 3652 3501
SmDDM1-like1 2688 12 2055 633
PaDDM1-like1
PaDDM1-like2
13905 16 2415 11490
AtrDDM1-like1 17482 15 2301 15181
ZmDDM1-like1
ZmDDM1-like2
6719 15 3584 3135
SbDDM1-like1
SbDDM1-like2
6731 15 3187 3544
OsDDM1-like1
OsDDM1-like2
6307 14 3121 3186
HvDDM1-like1 6791 13 2634 4157
SlDDM1-like1
SlDDM1-like2
10457 15 2547 7910
VvDDM1-like1 7510 17 2403 5107
ClDDM1-like1 7173 15 2229 4944
PvDDM1-like1 6013 16 2809 3204
PtDDM1-like1
PtDDM1-like2
7347 16 2859 4488
GrDDM1-like1 4966 15 2247 2719
TcDDM1-like1 6062 16 3302 2760
CpDDM1-like1 5378 15 2373 3005
AtDDM1-like1 4123 16 2598 1525
0kb 2kb 4kb 6kb 8kb 10kb 12kb 14kb 16kb 18kb 20kb 22kb
0 400 800 1200 1600
SMART DEXDc
BLAST DEXDc
SMART HELICc
Pfam Nol1_Nop2_Fmu
Pfam SNF2_N
Pfam DBINO
SMART cNMP
Prospero RPT1

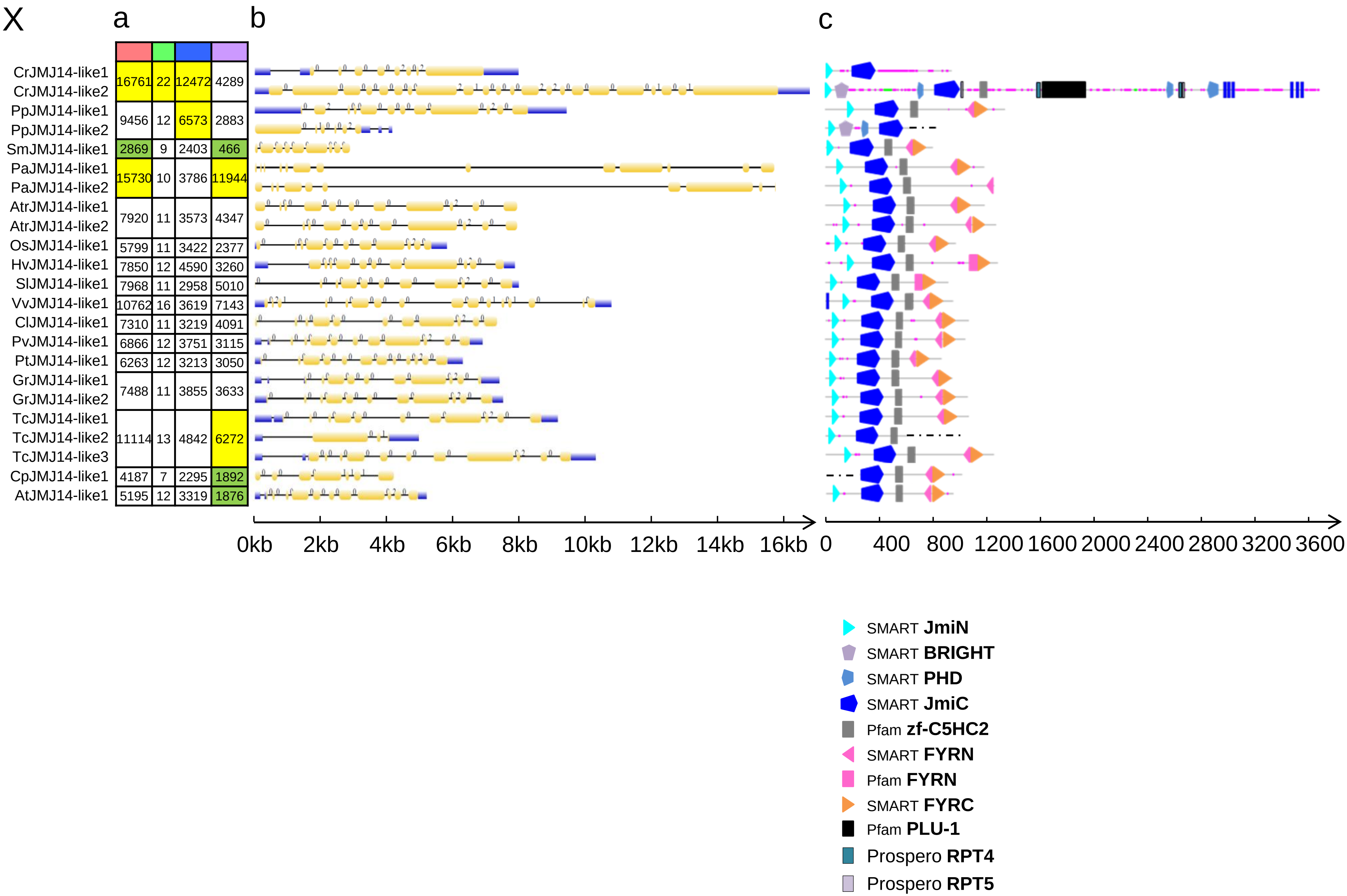
X
a
b
c
CrJMJ14-like1
CrJMJ14-like2
PpJMJ14-like1
PpJMJ14-like2
SmJMJ14-like1
PaJMJ14-like1
PaJMJ14-like2
AtrJMJ14-like1
AtrJMJ14-like2
OsJMJ14-like1
HvJMJ14-like1
SlJMJ14-like1
VvJMJ14-like1
ClJMJ14-like1
PvJMJ14-like1
PtJMJ14-like1
GrJMJ14-like1
GrJMJ14-like2
TcJMJ14-like1
TcJMJ14-like2
TcJMJ14-like3
CpJMJ14-like1
AtJMJ14-like1
16761 22 12472 4289
9456 12 6573 2883
2869 9 2403 466
15730 10 3786 11944
7920 11 3573 4347
5799 11 3422 2377
7850 12 4590 3260
7968 11 2958 5010
10762 16 3619 7143
7310 11 3219 4091
6866 12 3751 3115
6263 12 3213 3050
7488 11 3855 3633
11114 13 4842 6272
4187 7 2295 1892
5195 12 3319 1876
0kb 2kb 4kb 6kb 8kb 10kb 12kb 14kb 16kb
0 400 800 1200 1600 2000 2400 2800 3200 3600
SMART JmiN
SMART BRIGHT
SMART PHD
SMART JmiC
Pfam zf-C5HC2
SMART FYRN
Pfam FYRN
SMART FYRC
Pfam PLU-1
Prospero RPT4
Prospero RPT5

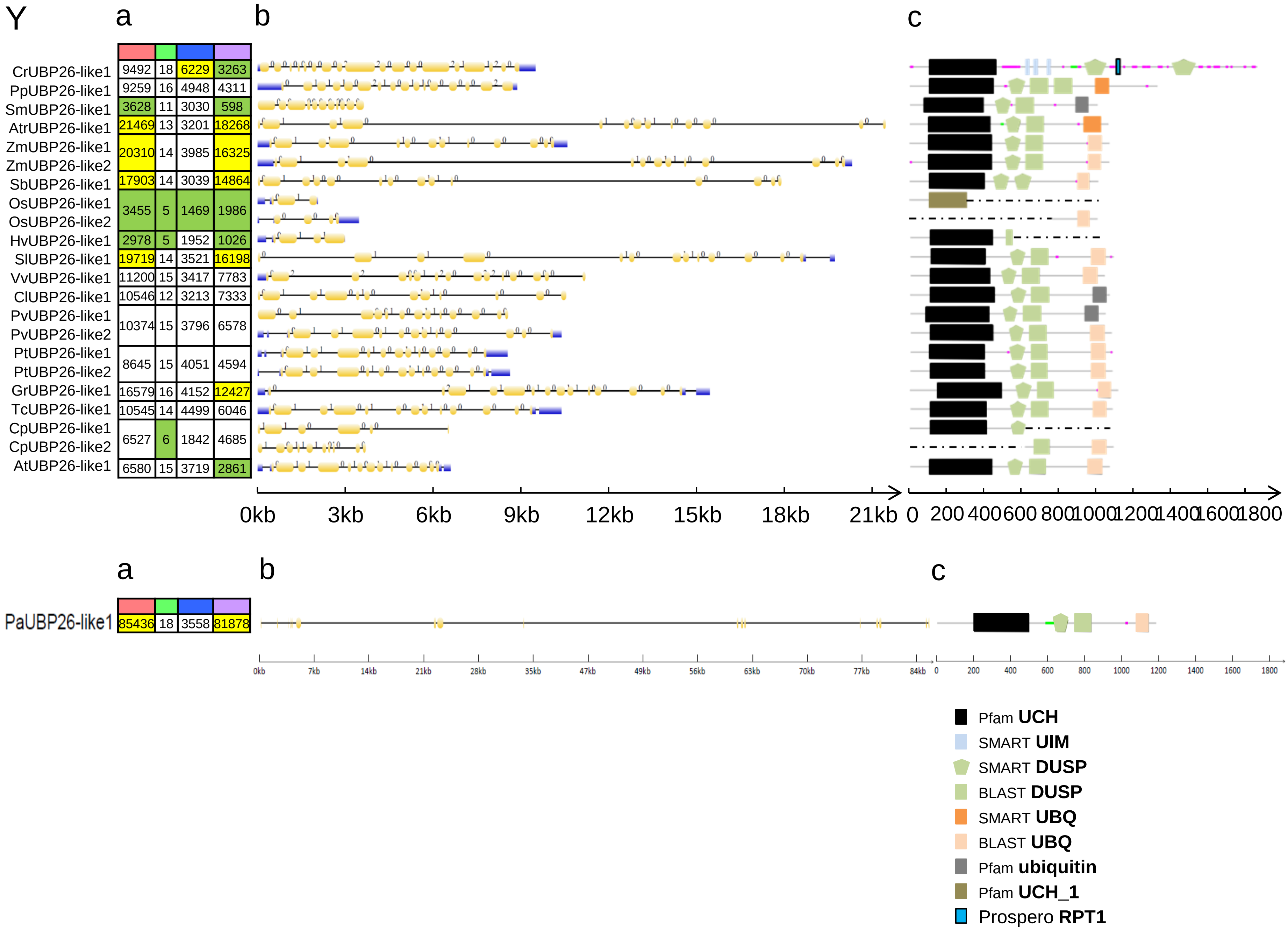

Y
a
b
c
CrUBP26-like1 9492 18 6229 3263
PpUBP26-like1 9259 16 4948 4311
SmUBP26-like1 3628 11 3030 598
AtrUBP26-like1 21469 13 3201 18268
ZmUBP26-like1
ZmUBP26-like2 20310 14 3985 16325
SbUBP26-like1 17903 14 3039 14864
OsUBP26-like1
OsUBP26-like2 3455 5 1469 1986
HvUBP26-like1 2978 5 1952 1026
SlUBP26-like1 19719 14 3521 16198
VvUBP26-like1 11200 15 3417 7783
ClUBP26-like1 10546 12 3213 7333
PvUBP26-like1
PvUBP26-like2 10374 15 3796 6578
PtUBP26-like1
PtUBP26-like2 8645 15 4051 4594
GrUBP26-like1 16579 16 4152 12427
TcUBP26-like1 10545 14 4499 6046
CpUBP26-like1
CpUBP26-like2 6527 6 1842 4685
AtUBP26-like1 6580 15 3719 2861
0kb 3kb 6kb 9kb 12kb 15kb 18kb 21kb
0 200 400 600 800 1000 1200 1400 1600 1800
a
b
c
PaUBP26-like1 85436 18 3558 81878
0kb 7kb 14kb 21kb 28kb 35kb 47kb 49kb 56kb 63kb 70kb 77kb 84kb
0 200 400 600 800 1000 1200 1400 1600 1800
Pfam UCH
SMART UIM
SMART DUSP
BLAST DUSP
SMART UBQ
BLAST UBQ
Pfam ubiquitin
Pfam UCH_1
Prospero RPT1

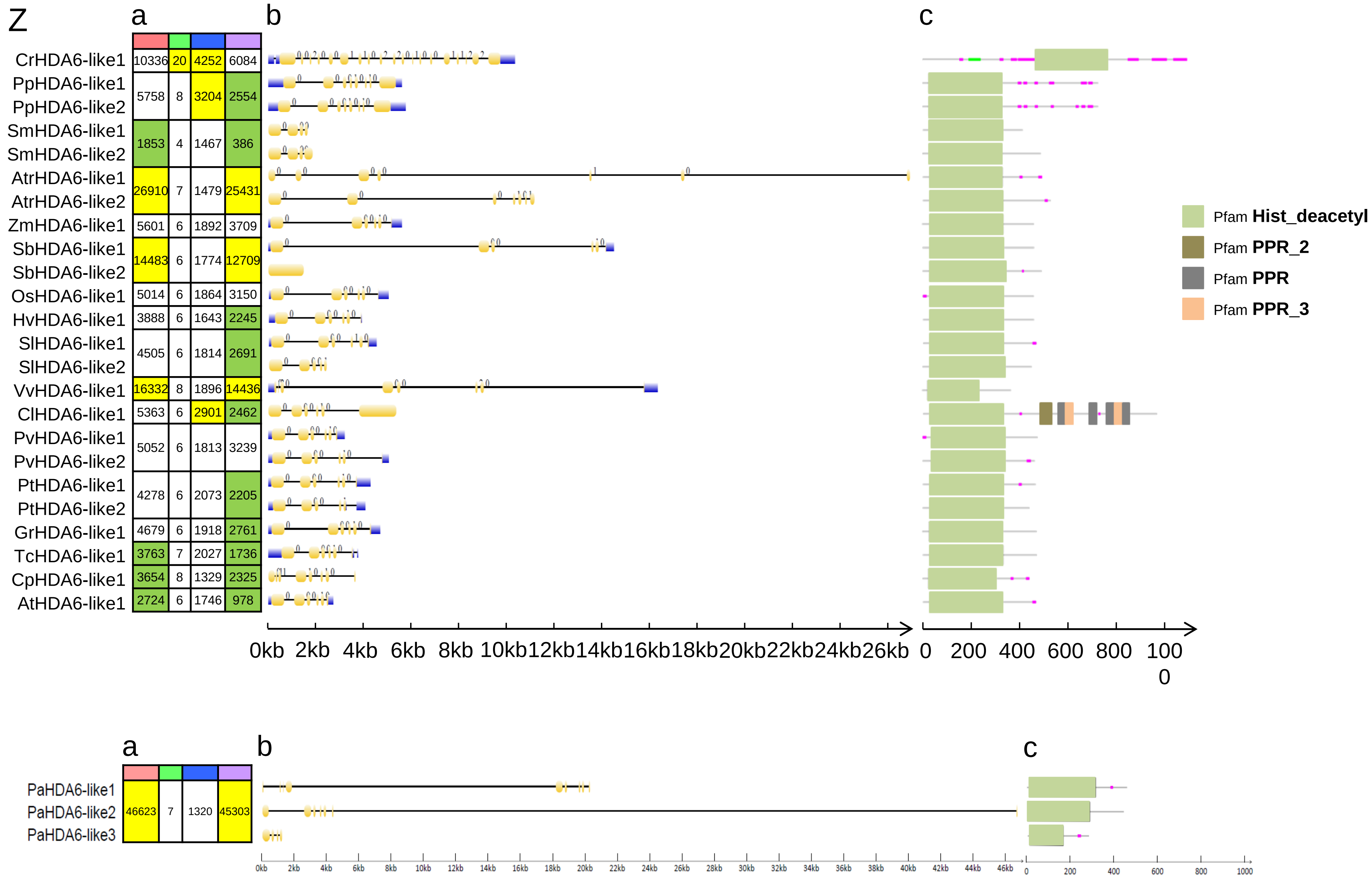
Z
a
b
c
CrHDA6-like1 10336 20 4252 6084
PpHDA6-like1
PpHDA6-like2
5758 8 3204 2554
SmHDA6-like1
SmHDA6-like2
1853 4 1467 386
AtrHDA6-like1
AtrHDA6-like2
26910 7 1479 25431
ZmHDA6-like1 5601 6 1892 3709
SbHDA6-like1
SbHDA6-like2
14483 6 1774 12709
OsHDA6-like1 5014 6 1864 3150
HvHDA6-like1 3888 6 1643 2245
SlHDA6-like1
SlHDA6-like2
4505 6 1814 2691
VvHDA6-like1 16332 8 1896 14436
ClHDA6-like1 5363 6 2901 2462
PvHDA6-like1
PvHDA6-like2
5052 6 1813 3239
PtHDA6-like1
PtHDA6-like2
4278 6 2073 2205
GrHDA6-like1 4679 6 1918 2761
TcHDA6-like1 3763 7 2027 1736
CpHDA6-like1 3654 8 1329 2325
AtHDA6-like1 2724 6 1746 978
0kb 2kb 4kb 6kb 8kb 10kb 12kb 14kb 16kb 18kb 20kb 22kb 24kb 26kb
0 200 400 600 800 100 0
Pfam Hist_deacetyl
Pfam PPR_2
Pfam PPR
Pfam PPR_3
a
b
c
PaHDA6-like1
PaHDA6-like2
PaHDA6-like3
46623 7 1320 45303
0kb 2kb 4kb 6kb 8kb 10kb 12kb 14kb 16kb 18kb 20kb 22kb 24kb 26kb 28kb 30kb 32kb 34kb 36kb 38kb 40kb 42kb 44kb 46kb
0 200 400 600 800 1000

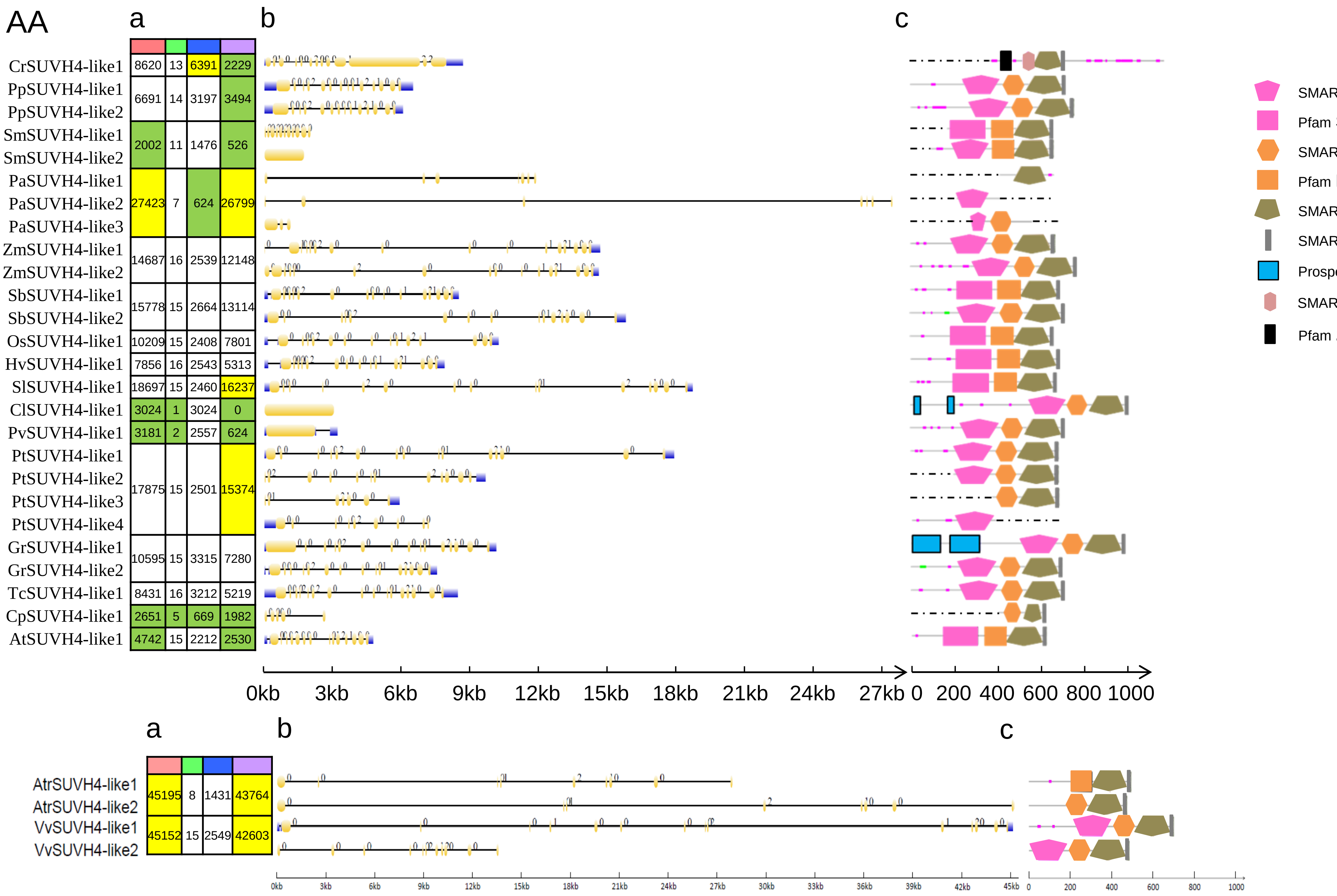

AA
a
b
c
CrSUVH4-like1 8620 13 6391 2229
PpSUVH4-like1
PpSUVH4-like2
6691 14 3197 3494
SmSUVH4-like1
SmSUVH4-like2
2002 11 1476 526
PaSUVH4-like1
PaSUVH4-like2
PaSUVH4-like3
27423 7 624 26799
ZmSUVH4-like1
ZmSUVH4-like2
14687 16 2539 12148
SbSUVH4-like1
SbSUVH4-like2
15778 15 2664 13114
OsSUVH4-like1 10209 15 2408 7801
HvSUVH4-like1 7856 16 2543 5313
SlSUVH4-like1 18697 15 2460 16237
ClSUVH4-like1 3024 1 3024 0
PvSUVH4-like1 3181 2 2557 624
PtSUVH4-like1
PtSUVH4-like2
PtSUVH4-like3
PtSUVH4-like4
17875 15 2501 15374
GrSUVH4-like1
GrSUVH4-like2
10595 15 3315 7280
TcSUVH4-like1 8431 16 3212 5219
CpSUVH4-like1 2651 5 669 1982
AtSUVH4-like1 4742 15 2212 2530
0kb 3kb 6kb 9kb 12kb 15kb 18kb 21kb 24kb 27kb
0 200 400 600 800 1000
SMAR
Pfam
SMAR
Pfam
SMAR
SMAR
Prosp
SMAR
Pfam
a
b
c
AtrSUVH4-like1
AtrSUVH4-like2
45195 8 1431 43764
VvSUVH4-like1
VvSUVH4-like2
45152 15 2549 42603
0kb 3kb 6kb 9kb 12kb 15kb 18kb 21kb 24kb 27kb 30kb 33kb 36kb 39kb 42kb 45kb
0 200 400 600 800 1000

BB

a

CrDME-like1	14782	15	10176	4606
PpDME-like1<br>PpDME-like2	11472	18	7774	3698
PaDME-like1	10500	4	413	10087
AtrDME-like1	15493	18	6399	9094
SbDME-like1<br>SbDME-like2	15972	18	6585	9387
OsDME-like2<br>OsDME-like3	11758	17	6437	5284
HvDME-like1	11550	16	6195	5355
SlDME-like1	9021	20	5610	3411
VvDME-like1	11943	24	6448	5495
ClDME-like1	9937	17	5145	4792
PtDME-like1<br>PtDME-like2	13269	18	5603	7666
GrDME-like1	11022	20	6473	4549
TcDME-like1	15111	20	6786	8325
CpDME-like1	16917	20	5670	11247
AtDME-like1	9209	18	6963	2246

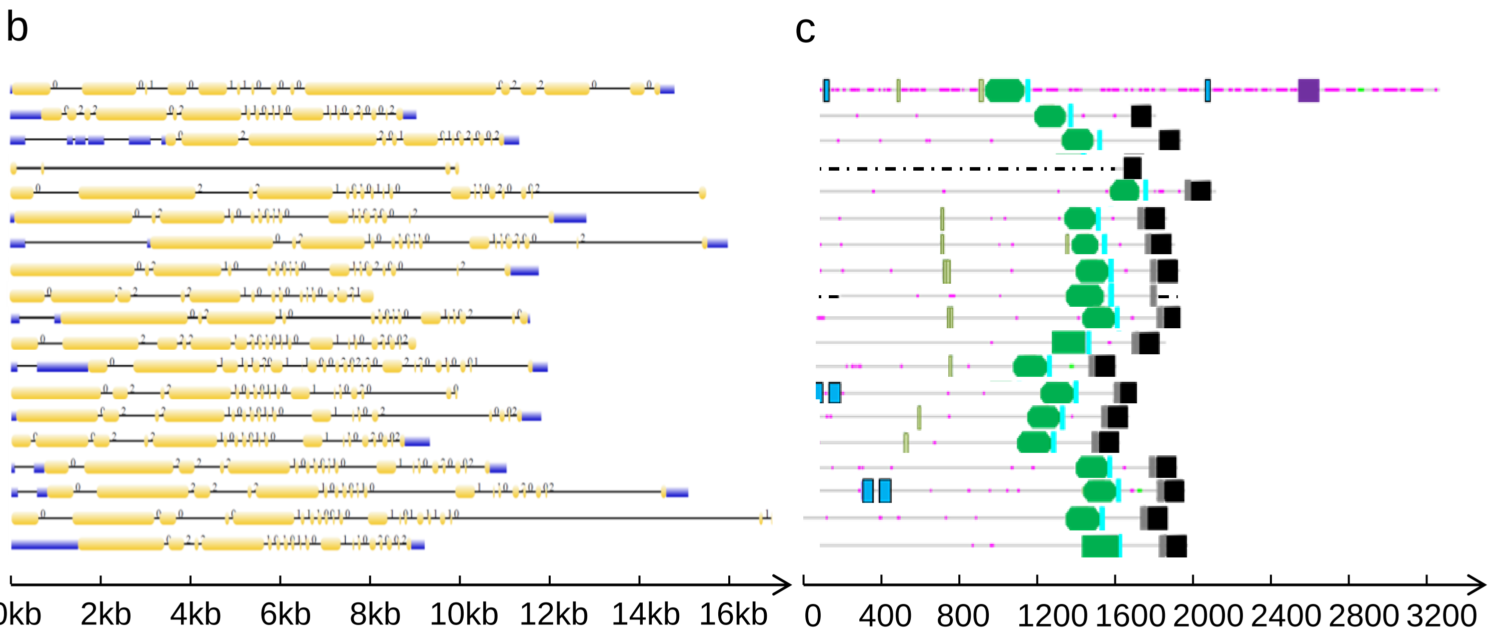

a

ZmDME-like1<br>ZmDME-like2	46430	16	5721	40709

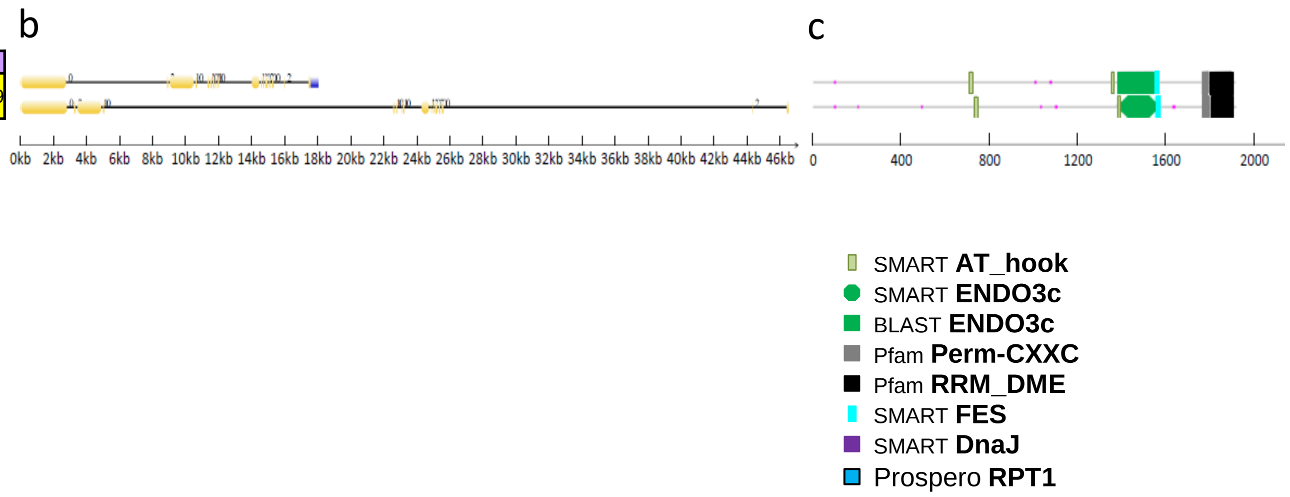

CC

a

PpROS1-like1	11057	23	6852	4205
SmROS1-like1	3451	17	2516	935
PaROS1-like1	8923	7	3501	5422
AtrROS1-like1	11995	18	5931	6064
ZmROS1-like1	10957	17	3270	7687
SbROS1-like1	10862	20	6220	4642
OsROS1-like2<br>OsROS1-like3	12675	20	5544	7131
HvROS1-like1	5768	16	2898	2870
SlROS1-like1<br>SlROS1-like2	9557	19	5475	4082
VvROS1-like2<br>VvROS1-like1	10752	27	5486	5266
ClROS1-like1<br>ClROS1-like2	15091	19	5601	9490
PvROS1-like1<br>PvROS1-like2	11357	20	7718	3639
PtROS1-like1	10138	18	5701	4437
GrROS1-like1<br>GrROS1-like2	16316	19	6094	10222
TcROS1-like1	11824	21	6893	4931
CpROS1-like1<br>CpROS1-like2	3545	6	2829	716
AtROS1-like1	6787	21	4713	2074

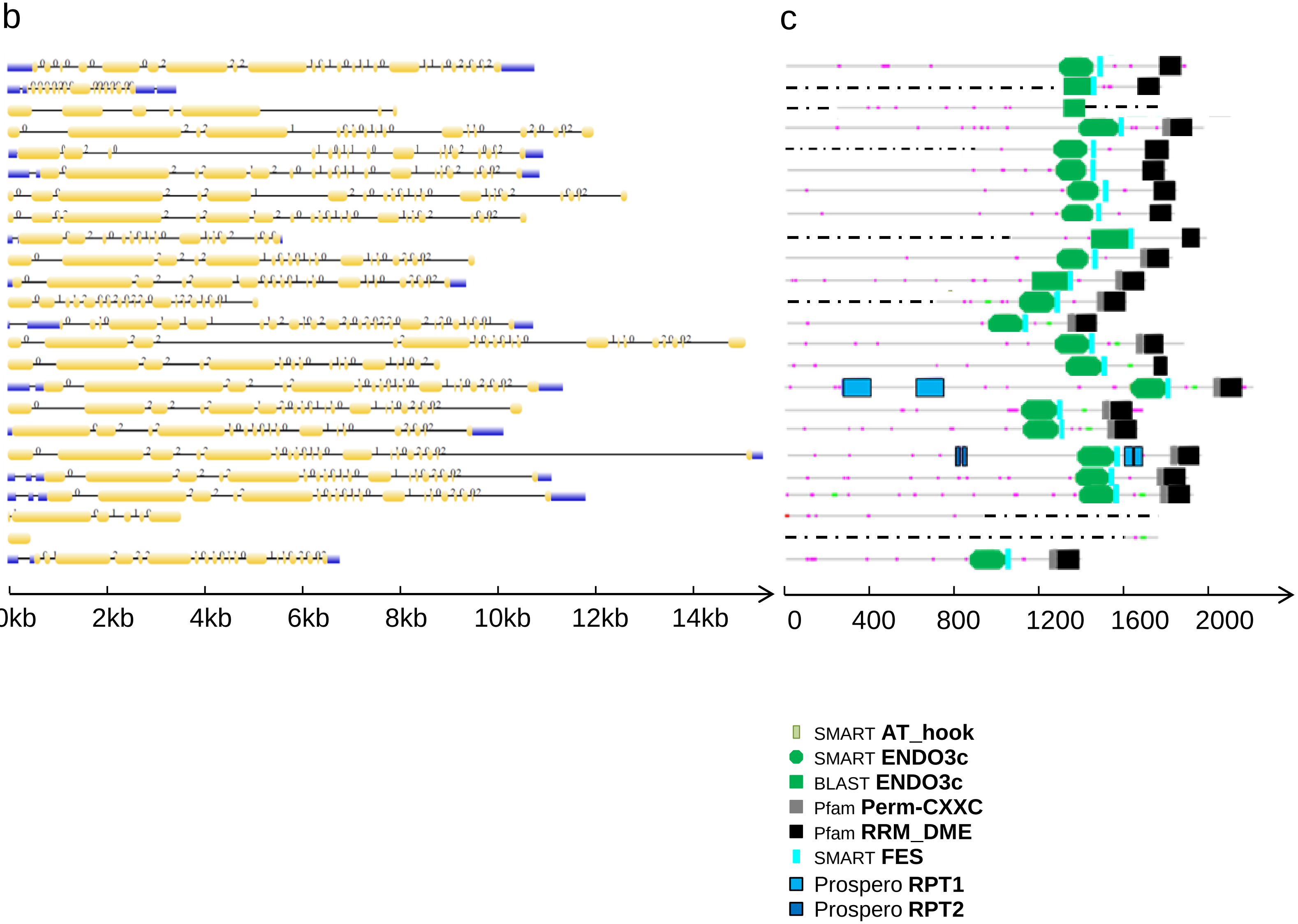

DD

a

SbDML3-like3<br>SbDML3-like2	9303	17	3761	5542
OsDML3-like1<br>OsDML3-like1	8806	17	3992	4814
HvDML3-like2	5776	13	1994	3782
SlDML3-like2	10224	22	4778	5446
PvDML3-like1	8180	17	2646	35534
GrDML3-like2	7861	19	5286	2575
TcDML3-like2	3795	11	2197	1598
AtDML3-like1	5424	20	3308	2116

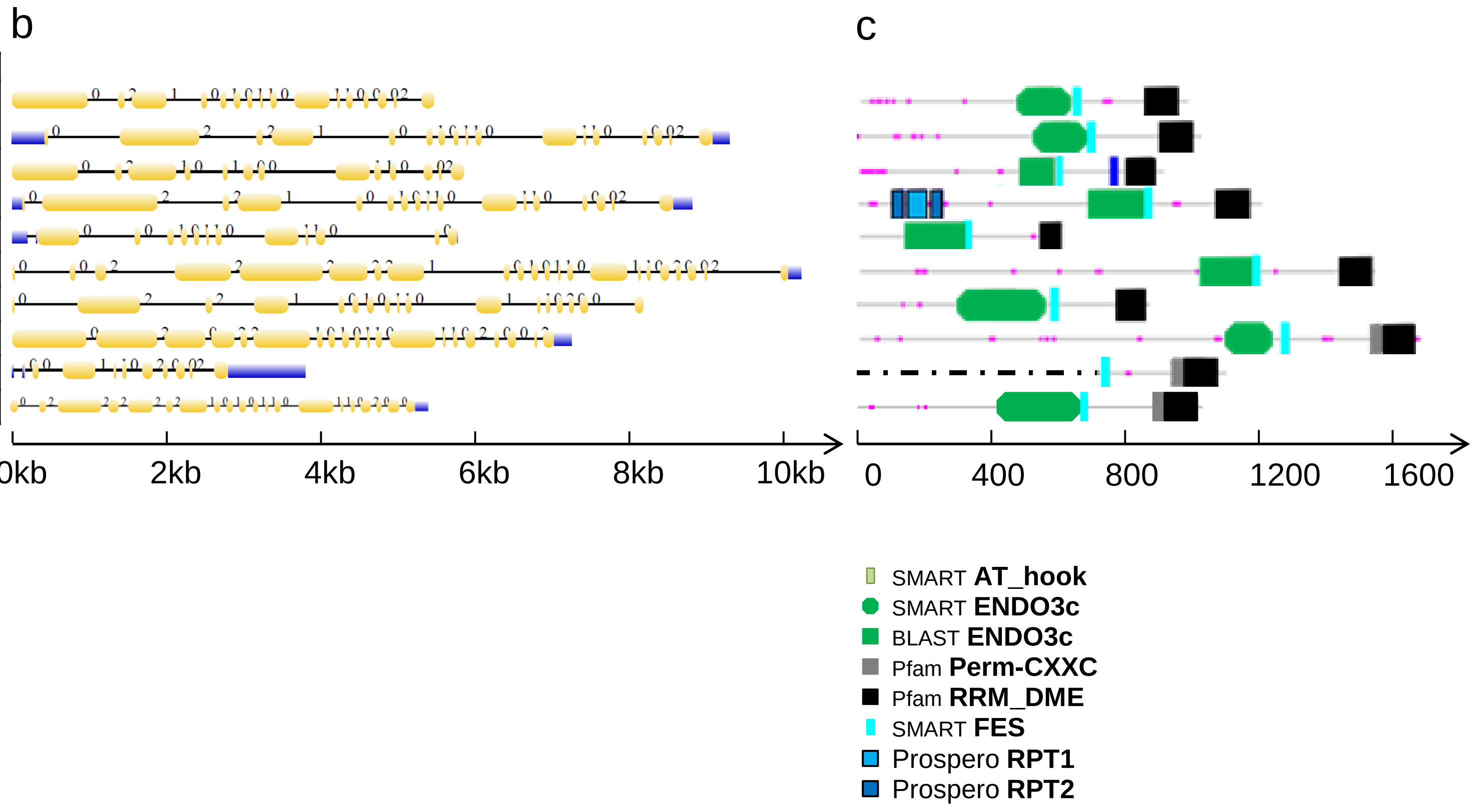

Supplement: Supplementary file 2 — Figure S2. Exon-intron structures and domain structures. (A-DD) Exon-intron structures and domain structures of RDR2, CLSY1, DCL3, HEN1, AGO4, RDM1, DRD1, MORC6, SUVH2, SUVH9, KTF1, DRM1, DRM2, JMJ14, UBP26, HDA6, SUVH4, IDN2, IDP1, NRPD1, NRPE1, NRPD/E2, NRPD/E4, NRPE5, CMT2, CMT3, DDM, DME, ROS1 and DML3. a: A summary of gene length, exon number, exon length and intron length of gene. The green and yellow in the table represent genes with extreme values. b: The gene structure was shown by the online tool GSDS. c: The domain structures were shown by the online tool SMART. The species name are Chlamydomonas reinhardtii (Cr), Physcomitrella patens (Pp), Selaginella moellendorffii (Sm), Amborella trichopoda (Atr), Zea mays (Zm), Sorghum bicolor (Sb), Oryza sativa (Os), Hordeum vulgare (Hv), Solanum lycopersicum (Sl), Vitis vinifera (Vv), Citrullus lanatus (Cl), Phaseolus vulgaris (Pv), Populus trichocarpa (Pt), Gossypium raimondii (Gr), Theobroma cacao (Tc), Carica papaya (Cp), Arabidopsis thaliana (At). (PDF 4727 kb) [file 12870_2019_1923_MOESM2_ESM.pdf]
